# Supplementary material for: Health status of individuals referred to first-line intervention for hip and knee osteoarthritis compared with the general population: an observational register-based study
Source: BMJ Open. 2021 Sep 12;11(9):e049476. doi: 10.1136/bmjopen-2021-049476 (PMC8438840; doi:10.1136/bmjopen-2021-049476)
Supplement: Supplementary data [file bmjopen-2021-049476supp001.pdf]

Supplementary file 1. ATC codes used to identify comorbidities with the RxRisk Index[1]

Comorbidities	ATC codes
Alcohol dependency	N07BB01–N07BB99
Allergies	R01AC01–R01AD60, R06AD02–R06AX27, R06AB04
Anticoagulants	B01AA03–B01AB06, B01AE07, B01AF01, B01AF02, B01AX05
Antiplatelets	B01AC04–B01AC30
Anxiety	N05BA01–N05BA12, N05BE01
Arrhythmia	C01AA05, C01BA01–C01BD01, C07AA07
Benign prostatic hyperplasia	G04CA01–G04CA99, G04CB01, G04CB02*
Bipolar disorder	N05AN01
Chronic airways disease	R03AC02–R03DC03, R03DX05
Congestive heart failure	C03DA02–C03DA99, C07AB02, C07AB07, C07AG02, C07AB12, C03DA04 (C03CA01– C03CC01 and C09AA01–C09AX99, C09CA01– C09CX99)†
Dementia	N06DA02–N06DA04, N06DX01
Depression	N06AA01–N06AG02, N06AX03–N06AX11, N06AX13–N06AX18, N06AX21–N06AX26
Diabetes	A10AA01-A10BX99
Epilepsy	N03AA01–N03AX99
Glaucoma	S01EA01–S01EB03, S01EC03–S01EX99
Gastroesophageal reflux disease	A02BA01–A02BX05
Gout	M04AA01–M04AC01
HIV	J05AE01–J05AE10, J05AF12–J05AG05, J05AR01–J05AR99, J05AX07–J05AX09, J05AX12, J05AF01–J05AF07, J05AF09
Hyperkalaemia	V03AE01
Hyperlipidaemia	C10AA01–C10BX09
Hypertension	C03AA01–C03BA11, C03DB01, C03DB99, C03EA01, C09BA02–C09BA09, C09DA02– C09DA08, C02AB01–C02AC05, C02DB02– C02DB99 (C03CA01–C03CCO1 or C09CA01– C09CX99)§
Hyperthyroidism	H03BA02, H03BB01
Hypothyroidism	H03AA01–H03AA02
Irritable bowel syndrome	A07EC01–A07EC04, A07EA01–A07EA02, A07EA06, L04AA33
Ischaemic heart disease: angina	C01DA02–C01DA14, C01DX16, C08EX02
Ischaemic heart disease: hypertension	C07AA01–C07AA06, C07AA08–C07AB01, C07AB02, C07AG01, C08CA01–C08DB01, C09DB01–C09DB04, C09DX01, C09BB02– C09BB10, C07AB03, C09DX03
Incontinence	G04BD01–G04BD99
Inflammation/pain	M01AB01–M01AH06
Liver failure	A06AD11, A07AA11
Malignancies	L01AA01–L01XX41
Malnutrition	B05BA01–B05BA10
Migraine	N02CA01–N02CX01
Osteoporosis/Paget's	M05BA01–M05BB05, M05BX03, M05BX04, G03XC01, H05AA02
Pain	N02AA01–N02AX02, N02AX06, N02AX52, N02BE51

Pancreatic insufficiency	A09AA02
Parkinson's disease	N04AA01–N04BX02
Psoriasis	D05AA01–D05AA99, D05BB01, D05BB02, D05AX02, D05AC01–D05AC51, D05AX52
Psychotic illness	N05AA01–N05AB02, N05AB06–N05AL07, N05AX07–N05AX13
Pulmonary hypertension	C02KX01–C02KX05
Renal disease	B03XA01–B03XA03, A11CC01–A11CC04, V03AE02, V03AE03, V03AE05
Smoking cessation	N07BA01–N07BA03, N06AX12
Steroid-responsive disease	H02AB01–H02AB10
Transplant	L04AA06, L04AA10, L04AA18, L04AD01, L04AD02

1. Pratt NL, Kerr M, Barratt JD, et al. The validity of the Rx-Risk Comorbidity Index using medicines mapped to the Anatomical Therapeutic Chemical (ATC) Classification System. *BMJ Open* 2018;8(4):e021122.

ATC code: Anatomical Therapeutic Chemical code
*Benign prostatic hyperplasia medicines – must be male.
†Must have at least two medicines prescribed with one of those medicines having an ATC code from C03CA01–C03CC01 and the other having an ATC code from either C09AA01–C09AX99 or C09CA01–C09CX99, at some point within the period of three years prior to baseline
§Can have medicine dispensed with an ATC code C03CA01–C03CC01 or C09AA01–C09AX99, but not both, as this would indicate chronic heart failure.
