# Supplementary material for: Health status of individuals referred to first-line intervention for hip and knee osteoarthritis compared with the general population: an observational register-based study
Source: BMJ Open. 2021 Sep 12;11(9):e049476. doi: 10.1136/bmjopen-2021-049476 (PMC8438840; doi:10.1136/bmjopen-2021-049476)
Supplement: Supplementary data [file bmjopen-2021-049476supp002.pdf]

Supplementary file 2. ICD-10 codes used to identify comorbidities with the Charlson Comorbidity Index and the Elixhauser Comorbidity Index[1]

Comorbidities	ICD-10	
	**Charlson Comorbidity Index**	**Elixhauser Comorbidity Index**
AIDS/HIV	B20.x–B22.x, B24.x	B20.x–B22.x, B24.x
Alcohol abuse	-	F10, E52, G62.1, I42.6, K29.2, K70.0, K70.3, K70.9, T51.x, Z50.2, Z71.4, Z72.1
Cardiac arrhythmia	-	I44.1–I44.3, I45.6, I45.9, I47.x–I49.x, R00.0, R00.1, R00.8, T82.1, Z45.0, Z95.0
Blood loss anaemia	-	D50.0
Cerebrovascular disease	G45.x, G46.x, H34.0, I60.x-I69.x	-
Chronic pulmonary disease	I27.8, I27.9, J40.x–J47.x, J60.x–J67.x, J68.4, J70.1, J70.3	I27.8, I27.9, J40.x–J47.x, J60.x–J67.x, J68.4, J70.1, J70.3
Coagulopathy	-	D65–D68.x, D69.1, D69.3–D69.6
Congestive heart failure	I09.9, I11.0, I13.0, I13.2, I25.5, I42.0, I42.5–I42.9, I43.x, I50.x, P29.0	I09.9, I11.0, I13.0, I13.2, I25.5, I42.0, I42.5–I42.9, I43.x, I50.x, P29.0
Deficiency anaemia	-	D50.8, D50.9, D51.x–D53.x
Dementia	F00.x–F03.x, F05.1, G30.x, G31.1	-
Depression	-	F20.4, F31.3–F31.5, F32.x, F33.x, F34.1, F41.2, F43.2
Diabetes (uncomplicated)	E10.0, E10.1, E10.6, E10.8, E10.9, E11.0, E11.1, E11.6, E11.8, E11.9, E12.0, E12.1, E12.6, E12.8, E12.9, E13.0, E13.1, E13.6, E13.8, E13.9, E14.0, E14.1, E14.6, E14.8, E14.9	E10.0, E10.1, E10.9, E11.0, E11.1, E11.9, E12.0, E12.1, E12.9, E13.0, E13.1, E13.9, E14.0, E14.1, E14.9
Diabetes (complicated)	E10.2–E10.5, E10.7, E11.2–E11.5, E11.7, E12.2–E12.5, E12.7, E13.2–E13.5, E13.7, E14.2–E14.5, E14.7	E10.2–E10.8, E11.2–E11.8, E12.2–E12.8, E13.2–E13.8, E14.2–E14.8
Drug abuse	-	F11.x–F16.x, F18.x, F19.x, Z71.5, Z72.2
Fluid and electrolyte disorders	-	E22.2, E86.x, E87.x
Hemiplegia or paraplegia	G04.1, G11.4, G80.1, G80.2, G81.x, G82.x, G83.0–G83.4, G83.9	-
Hypertension[a]	-	I10.x, I11.x–I13.x, I15.x
Hypothyroidism	-	E00.x–E03.x, E89.0
Liver disease	B18.x, K70.0–K70.3, K70.9, K71.3–K71.5, K71.7, K73.x, K74.x, K76.0, K76.2–K76.4, K76.8, K76.9, Z94.4	B18.x, I85.x, I86.4, I98.2, K 70.x, K71.1, K71.3–K71.5, K71.7, K72.x–K74.x, K76.0, K76.2–K76.9, Z94.4
Liver failure	I85.0, I85.9, I86.4, I98.2, K70.4, K71.1, K72.1, K72.9, K76.5, K76.6, K76.7	-
Lymphoma	-	C81.x–C85.x, C88.x, C96.x, C90.0, C90.2
Malignancies	C00.x–C26.x, C30.x–C34.x, C37.x–C41.x, C43.x, C45.x–C58.x, C60.x–C76.x, C81.x–C85.x, C88.x, C90.x–C97.x	-
Metastatic cancer	C77.x–C80.x	C77.x–C80.x
Myocardial infarction	I21.x, I22.x, I25.2	-
Obesity	-	E66.x
Other neurologic disorders	-	G10.x–G13.x, G20.x–G22.x, G25.4, G25.5, G31.2, G31.8, G31.9, G32.x, G35.x–G37.x, G40.x, G41.x, G93.1, G93.4, R47.0, R56.x
Paralysis	-	G04.1, G11.4, G80.1, G80.2, G81.x, G82.x, G83.0–G83.4, G83.9
Peptic ulcer disease	K25.x–K28.x	K25.7, K25.9, K26.7, K26.9, K27.7, K27.9, K28.7, K28.9
Peripheral vascular disease	I70.x, I71.x, I73.1, I73.8, I73.9, I77.1, I79.0, I79.2, K55.1, K55.8, K55.9, Z95.8, Z95.9	I70.x, I71.x, I73.1, I73.8, I73.9, I77.1, I79.0, I79.2, K55.1, K55.8, K55.9, Z95.8, Z95.9
Pulmonary circulation disorders	-	I26.x, I27.x, I28.0, I28.8, I28.9
Psychoses	-	F20.x, F22.x–F25.x, F28.x, F29.x, F30.2, F31.2, F31.5

Renal disease	I12.0, I13.1, N03.2–N03.7, N05.2–N05.7, N18.x, N19.x, N25.0, Z49.0–Z49.2, Z94.0, Z99.2	I12.0, I13.1, N18.x, N19.x, N25.0, Z49.0–Z49.2, Z94.0, Z99.2
Rheumatoid arthritis/collage vascular diseases		L94.0, L94.1, L94.3, M05.x, M06.x, M08.x, M12.0, M12.3, M30.x, M31.0–M31.3, M32.x–M35.x, M45.x, M46.1, M46.8, M46.9
Rheumatic disease	M05.x, M06.x, M31.5, M32.x–M34.x, M35.1, M35.3, M36.0	
Solid tumour without metastasis		C00.x–C26.x, C30.x–C34.x, C37.x–C41.x, C43.x, C45.x–C58.x, C60.x–C76.x, C97.x
Valvular disease		A52.0, I05.x–I08.x, I09.1, I09.8, I34.x–I39.x, Q23.0–Q23.3, Z95.2–Z95.4
Weight loss	-	E40.x–E46.x, R63.4, R64

1. Quan H, Sundararajan V, Halfon P, et al. Coding algorithms for defining comorbidities in ICD-9-CM and ICD-10 administrative data. *Med Care* 2005;43(11):1130-9.

[a]Complicated and uncomplicated combined
