# Supplementary material for: Health status of individuals referred to first-line intervention for hip and knee osteoarthritis compared with the general population: an observational register-based study
Source: BMJ Open. 2021 Sep 12;11(9):e049476. doi: 10.1136/bmjopen-2021-049476 (PMC8438840; doi:10.1136/bmjopen-2021-049476)
Supplement: Supplementary data [file bmjopen-2021-049476supp003.pdf]

Supplementary file 3. Secondary analyses, presenting odds ratio (OR) of having comorbidities for the OA population in the BOA Register with index date between 2008 and 2016, compared with the reference cohort.

	**Total study population**			**Hip OA**			**Knee OA**		
	**Crude OR** (95% CI) **≤2015**	**Adjusted OR** (95% CI)[a] **≤2015**	**Adjusted OR** (95% CI)[b] **≤2015**	**Crude OR** (95% CI) **≤2015**	**Adjusted OR** (95% CI)[a] **≤2015**	**Adjusted OR** (95% CI)[b] **≤2015**	**Crude OR** (95% CI) **≤2015**	**Adjusted OR** (95% CI)[a] **≤2015**	**Adjusted OR** (95% CI)[b] **≤2015**
**Individual comorbidities**[b]									
**≥1**	1.63 (1.58, 1.67)*	1.68 (1.63, 1.72)*	1.70 (1.66, 1.75)*	1.47 (1.40, 1.53)*	1.52 (1.45, 1.59)*	1.54 (1.47, 1.61)*	1.71 (1.65, 1.76)*	1.75 (1.70, 1.81)*	1.78 (1.73, 1.84)*
**≥2**	1.36 (1.33, 1.39)*	1.41 (1.39, 1.45)*	1.44 (1.41, 1.47)*	1.24 (1.19, 1.29)*	1.29 (1.25, 1.34)*	1.32 (1.27, 1.37)*	1.42 (1.39, 1.46)*	1.47 (1.44, 1.51)*	1.50 (1.46, 1.54)*
**≥3**	1.23 (1.21, 1.26)*	1.28 (1.26, 1.31)*	1.31 (1.28, 1.33)*	1.12 (1.08, 1.16)*	1.17 (1.13, 1.21)*	1.19 (1.14, 1.23)*	1.28 (1.26, 1.32)*	1.34 (1.31, 1.37)*	1.36 (1.33, 1.40)*
**Disease categories**									
**Cancer**	0.88 (0.84, 0.92)*	0.87 (0.84, 0.91)*	0.87 (0.84, 0.91)*	0.88 (0.82, 0.95)*	0.87 (0.81, 0.94)*	0.87 (0.81, 0.94)*	0.88 (0.83, 0.92)*	0.88 (0.83, 0.92)*	0.87 (0.83, 0.92)*
**Cardiovascular/ blood**	1.23 (1.20, 1.25)*	1.27 (1.25, 1.30)*	1.29 (1.26, 1.32)*	1.14 (1.10, 1.18)*	1.19 (1.14, 1.23)*	1.20 (1.16, 1.25)*	1.27 (1.24, 1.30)*	1.31 (1.28, 1.34)*	1.33 (1.30, 1.37)*
**Endocrine**	1.04 (1.02, 1.07)*	1.09 (1.06, 1.12)*	1.09 (1.07, 1.12)*	0.96 (0.92, 1.01)	1.02 (0.97, 1.07)	1.02 (0.98, 1.07)	1.08 (1.05, 1.11)*	1.13 (1.10, 1.16)*	1.13 (1.10, 1.16)*
**Gastrointestinal**	1.39 (1.36, 1.42)*	1.45 (1.42, 1.48)*	1.46 (1.43, 1.49)*	1.29 (1.24, 1.34)*	1.35 (1.30, 1.41)*	1.36 (1.31, 1.41)*	1.43 (1.40, 1.47)*	1.49 (1.45, 1.53)*	1.50 (1.46, 1.54)*
**Musculoskeletal/ pain-related**[c]	1.03 (1.00, 1.07)*	1.04 (1.01, 1.08)*	1.04 (1.01, 1.08)*	1.04 (0.98, 1.10)	1.05 (0.99, 1.11)	1.05 (0.99, 1.11)	1.03 (0.99, 1.07)	1.04 (1.00, 1.08)	1.04 (1.00, 1.08)
**Neurologic**	0.78 (0.75, 0.81)*	0.81 (0.77, 0.84)*	0.81 (0.78, 0.84)*	0.71 (0.66, 0.76)*	0.73 (0.68, 0.79)*	0.73 (0.68, 0.79)*	0.81 (0.78, 0.85)*	0.84 (0.80, 0.88)*	0.84 (0.80, 0.88)*

**Nutritional/obesity**	0.94 (0.89, 1.00)*	0.99 (0.94, 1.05)	1.00 (0.94, 1.06)	0.75 (0.67, 0.84)*	0.79 (0.71, 0.88)*	0.80 (0.71, 0.89)*	1.03 (0.97, 1.10)	1.09 (1.02, 1.16)*	1.10 (1.02, 1.17)*
**Psychological/ behavioural**	0.96 (0.94, 0.98)*	0.99 (0.97, 1.02)	0.99 (0.97, 1.02)	0.92 (0.89, 0.96)*	0.95 (0.92, 0.99)*	0.96 (0.92, 1.00)*	0.97 (0.95, 1.00)*	1.01 (0.98, 1.04)	1.01 (0.98, 1.04)
**Renal/urologic**	1.32 (1.28, 1.37)*	1.34 (1.30, 1.39)*	1.35 (1.30, 1.40)*	1.23 (1.16, 1.31)*	1.26 (1.18, 1.34)*	1.27 (1.19, 1.35)*	1.37 (1.31, 1.43)*	1.39 (1.33, 1.44)*	1.39 (1.33, 1.45)*
**Respiratory**	1.16 (1.12, 1.19)*	1.18 (1.14, 1.21)*	1.18 (1.14, 1.21)*	1.09 (1.03, 1.15)*	1.11 (1.05, 1.18)*	1.12 (1.06, 1.18)*	1.19 (1.15, 1.23)*	1.20 (1.16, 1.25)*	1.20 (1.16, 1.25)*
**Miscellaneous**	1.30 (1.28, 1.33)*	1.30 (1.28, 1.33)*	1.30 (1.28, 1.33)*	1.23 (1.19, 1.28)*	1.23 (1.19, 1.28)*	1.23 (1.19, 1.28)*	1.34 (1.31, 1.37)*	1.34 (1.30, 1.37)*	1.34 (1.31, 1.37)*

[a]Adjusted for socioeconomic status (educational level, country of birth and marital status).

[b]Adjusted for socioeconomic status (educational level, country of birth, marital status, income and employment, and family type).

*Statistically significant results.
