# Supplementary material for: Health status of individuals referred to first-line intervention for hip and knee osteoarthritis compared with the general population: an observational register-based study
Source: BMJ Open. 2021 Sep 12;11(9):e049476. doi: 10.1136/bmjopen-2021-049476 (PMC8438840; doi:10.1136/bmjopen-2021-049476)
Supplement: Author's manuscript [file bmjopen-2021-049476.draft_revisions.pdf]

BMJ Open is committed to open peer review. As part of this commitment we make the peer review history of every article we publish publicly available.

When an article is published we post the peer reviewers' comments and the authors' responses online. We also post the versions of the paper that were used during peer review. These are the versions that the peer review comments apply to.

The versions of the paper that follow are the versions that were submitted during the peer review process. They are not the versions of record or the final published versions. They should not be cited or distributed as the published version of this manuscript.

BMJ Open is an open access journal and the full, final, typeset and author-corrected version of record of the manuscript is available on our site with no access controls, subscription charges or pay-per-view fees (http://bmjopen.bmj.com).

If you have any questions on BMJ Open's open peer review process please email info.bmjopen@bmj.com

BMJ Open

Health status of individuals referred to first-line intervention for hip and knee osteoarthritis compared with the general population: an observational register-based study

Journal:	*BMJ Open*
Manuscript ID	bmjopen-2021-049476
Article Type:	Original research
Date Submitted by the Author:	25-Jan-2021

Complete List of Authors:	Gustafsson , Kristin; Linköping University, Unit of Physiotherapy, Department of Health, Medicine and Caring Sciences,; Ryhov County Hospital, Department of Physiotherapy, Rehabilitation Centre,<br>Kvist, Joanna; Linköping University, Unit of Physiotherapy, Department of Health, Medicine and Caring Sciences,<br>Eriksson , Marit ; Linköping University and Futurum - Academy for Health and Care, Department of Medical and Health Sciences<br>Dell'Isola, Andrea; Lund University, Department of Clinical Sciences Lund<br>Zhou, Caddie; Centre of Registries, Västra Götaland,<br>Dahlberg, L; Lund University, Department of Clinical Sciences Lund<br>Rolfson, O; University of Gothenburg, Department of Orthopaedics

Keywords:	Hip < ORTHOPAEDIC & TRAUMA SURGERY, Knee < ORTHOPAEDIC & TRAUMA SURGERY, EPIDEMIOLOGY, Musculoskeletal disorders < ORTHOPAEDIC & TRAUMA SURGERY

SCHOLARONE™
Manuscripts

I, the Submitting Author has the right to grant and does grant on behalf of all authors of the Work (as defined in the below author licence), an exclusive licence and/or a non-exclusive licence for contributions from authors who are: i) UK Crown employees; ii) where BMJ has agreed a CC-BY licence shall apply, and/or iii) in accordance with the terms applicable for US Federal Government officers or employees acting as part of their official duties; on a worldwide, perpetual, irrevocable, royalty-free basis to BMJ Publishing Group Ltd ("BMJ") its licensees and where the relevant Journal is co-owned by BMJ to the co-owners of the Journal, to publish the Work in this journal and any other BMJ products and to exploit all rights, as set out in our licence.

The Submitting Author accepts and understands that any supply made under these terms is made by BMJ to the Submitting Author unless you are acting as an employee on behalf of your employer or a postgraduate student of an affiliated institution which is paying any applicable article publishing charge ("APC") for Open Access articles. Where the Submitting Author wishes to make the Work available on an Open Access basis (and intends to pay the relevant APC), the terms of reuse of such Open Access shall be governed by a Creative Commons licence – details of these licences and which Creative Commons licence will apply to this Work are set out in our licence referred to above.

Other than as permitted in any relevant BMJ Author's Self Archiving Policies, I confirm this Work has not been accepted for publication elsewhere, is not being considered for publication elsewhere and does not duplicate material already published. I confirm all authors consent to publication of this Work and authorise the granting of this licence.

For peer review only

Health status of individuals referred to first-line intervention for hip and knee osteoarthritis compared with the general population: an observational register-based study

Kristin Gustafsson,[1,2] Joanna Kvist,[1] Marit Eriksson,[3] Andrea Dell'Isola,[4] Caddie Zhou,[5] Leif E. Dahlberg,[4] Ola Rolfson[6]

[1]Unit of Physiotherapy, Department of Health, Medicine and Caring Sciences, Linköping University, Linköping, Sweden

[2]Department of Physiotherapy, Rehabilitation Centre, Ryhov County Hospital, Jönköping, Sweden

[3]Department of Medical and Health Sciences, Linköping University and Futurum - Academy for Health and Care, Region Jönköping County, Jönköping, Sweden

[4]Department of Clinical Sciences Lund, Orthopaedics, Faculty of Medicine, Lund University, Lund, Sweden

[5]Centre of RegistriesVästra Götaland, Gothenburg, Sweden

[6]Department of Orthopaedics, Sahlgrenska Academy, University of Gothenburg, Gothenburg, Sweden

E-mail:

Kristin Gustafsson – kristin.gustafsson@liu.se, kristin.gustafsson@rjl.se

Joanna Kvist – joanna.kvist@liu.se

Marit Eriksson – marit.eriksson@rjl.se

Andrea Dell'Isola - andrea.dellisola@med.lu.se

For peer review only - http://bmjopen.bmj.com/site/about/guidelines.xhtml

Caddie Zhou –_caddie.zhou@vgregion.se

Leif E Dahlberg – leif.dahlberg@ med.lu.se

Ola Rolfson – ola.rolfson@vgregion.se

Correspondence to Kristin Gustafsson, Unit of Physiotherapy, Department of Health, Medicine and Caring Sciences, Linköping University, Linköping, Sweden. E-mail: kristin.gustafsson@liu.se; kristin.gustafsson@rjl.se

ORCID:

Kristin Gustafsson: 0000-0003-3403-229X

Joanna Kvist: 0000-0003-3527-5488

Marit Eriksson: 0000-0002-1766-5899

Andrea Dell'Isola: 0000-0002-0319-458X

Caddie Zhou: 0000-0002-1669-2318

Leif Dahlberg: 0000-0001-7838-9464

Ola Rolfson: 0000-0001-6534-1242

For peer review only

WORD COUNT
3 694 words

NUMBER OF FIGURES AND TABLES
Two figures and three tables.

For peer review only - http://bmjopen.bmj.com/site/about/guidelines.xhtml

ABSTRACT

Objectives To describe the prevalence of comorbidities in a population referred to standardized first-line intervention for hip and knee osteoarthritis (OA), with patient education and exercise, in comparison with the general population. Furthermore, we aimed to evaluate if eventual differences were associated with socioeconomic inequalities.

Design Register-based study.

Setting Primary health care, Sweden.

Participants Individuals with hip and/or knee OA included in the Better Management for Patients with Osteoarthritis Register between 2008 and 2016, and an age-, sex- and residence-matched reference cohort (1:3) from the general Swedish population.

Outcome measures Comorbidities were identified with the RxRisk Index, the Elixhauser Comorbidity Index and the Charlson Comorbidity Index, and presented with descriptive statistics as (1) individual diseases, (2) disease categories and (3) scores for each index. The prevalence of comorbidities in the two populations was tested using logistic regression, with separate analyses for age groups and the most affected joint. We then adjusted the analyses for socioeconomic status.

Results In this OA population, 85% had at least one comorbidity compared with 78% of the reference cohort, with an odds ratio (OR) of 1.62 (95% confidence interval [CI] 1.59 to 1.66). Cardiovascular/blood diseases were the most common comorbidities in both populations (OA, 59%; reference, 54%), with an OR of 1.22 (95% CI 1.20 to 1.24) for the OA population. Younger individuals with OA were more comorbid than their matched references overall, and population differences decreased with age. Individuals with knee OA were more comorbid than those with hip OA overall. Adjustment for socioeconomic status did not change the estimates.

Conclusion Comorbidities were more common among individuals with hip and knee OA than among matched references from the general population. The differences could not be explained by socioeconomic status.

Trial registration NCT03438630, posted 20 February 2018.

Keywords Hip, Knee, Osteoarthritis, Patient education, Exercise, Comorbidity, Registries

For peer review only - http://bmjopen.bmj.com/site/about/guidelines.xhtml

Study protocol doi:10.1136/bmjopen-2018-022812

ARTICLE SUMMERY

Strengths and limitations of this study

- This study describes comorbidities in a nationwide OA population in the 3 years before participation in a standardized first-line intervention for hip and knee OA, compared with matched individuals from the general population.
- The study captures data on comorbidities from diagnostic codes (ICD-10 codes) and prescription of drugs (ATC codes) using three comorbidity indices: the RxRisk Index, the Elixhauser Comorbidity Index and the Charlson Comorbidity Index.
- To determine if socioeconomic inequalities could explain differences in comorbidity between the populations, the results were adjusted for highest educational level, country of birth and marital status.
- In this study we were only able to capture ICD-10 codes from health care delivered through inpatient care and specialized outpatient visits; the ATC codes included data on drugs prescribed in primary health care, in hospitals, by private care givers and occupational health care.

For peer review only

For peer review only - http://bmjopen.bmj.com/site/about/guidelines.xhtml

INTRODUCTION

Osteoarthritis (OA) is the most common musculoskeletal disorder in older adults.[1] Approximately 25% of the population more than 45 years old have OA, without accounting for the large numbers who never seek health care for their problem.[2] OA develops slowly over time, leading to pain, joint stiffness and functional limitations.[3, 4]

Co-existence of comorbidities is common among individuals with OA. More than 65% have at least one other chronic condition, which increases with age.[5, 6] The most frequent pattern of OA comorbidities includes cardiovascular and/or metabolic diseases[7] as well as psychological comorbidities such as depressive symptoms.[8, 9] The higher frequency of specific comorbidities in people with OA can be partially explained by an increased vulnerability to develop other diseases because several risk factors, such as sex, physical inactivity, obesity and lower socioeconomic status (SES), are common to many conditions.[5-7] Comorbidities increase the risk of hospitalization, polypharmacy, premature death and substantially extend the burden on the health care system.[10] In patients with OA, the presence of comorbidities also has impacts on disease severity with increased pain, inferior physical functioning,[11] and worsening of health-related quality of life[12] which may affect the treatment outcome.

First-line intervention for hip and knee OA comprises patient education and exercise combined with weight loss when necessary,[13-15] and is recommended regardless of the severity of the disease.[16, 17] This treatment, when delivered in a real-world setting, has shown to alleviate OA symptoms, give patients a satisfactory quality of life, decrease willingness to undergo surgery, reduce use of OA medication, and reduce sick leave.[18] However, those results include all individuals, regardless of the prevalence of comorbidities that may affect their outcome. To evaluate and improve treatment outcomes from first-line

For peer review only - http://bmjopen.bmj.com/site/about/guidelines.xhtml

intervention for hip and knee OA, the magnitude of comorbidities should be described. The aim of the present study was to determine comorbidities, including distribution depending on age, of a population with hip and/or knee OA who were referred to first-line intervention in comparison with the general population. A secondary aim was to evaluate if differences in comorbidities were associated with socioeconomic inequalities.

METHODS

Study design and participants

This observational register-based study included all patients with hip and/or knee OA (n=72 069) in the Better Management of Patients with Osteoarthritis (BOA) national quality register between 2008 and 2016 (table 1). The BOA Register, register and evaluate patients in a standardized national first-line intervention programme for hip and knee OA according to international guidelines,[14, 15] and provided by health professionals in primary care in Sweden.[17] Patients included in the present study were registered before participation (index date) and included regardless of compliance with treatment. They had sought help for hip and/or knee pain in primary health care and had been referred to the first-line intervention programme after a confirmed clinical or radiographic diagnosis of OA following the Swedish National Board of Health and Welfare recommendations.[19-21] Patients with joint problems caused by another condition (e.g., sequelae of hip fractures, chronic widespread pain, inflammatory joint diseases or cancer), patients who have undergone total joint replacement within the previous 12 months or other surgery of the knee or hip joint within the previous 3 months do not meet the criteria for inclusion in the BOA Register.[17] The standardized first-line intervention for OA, the development of the BOA Register and the inclusion and exclusion criteria for registration of patients in the register have been described in detail previously.[17, 22]

For peer review only - http://bmjopen.bmj.com/site/about/guidelines.xhtml

Without involvement from the researchers, Statistics Sweden (a government agency) randomly selected a reference cohort (n=216 207) of individuals from the general Swedish population for comparison using the Swedish Total Population Register (TPR)[23] (table 1). Those individuals had never been included in the BOA Register. To minimize confounding due to differences in access to care, the populations were matched for year of birth, sex and place of residence (counties in Sweden, n=21), at the index date in the BOA Register. Three reference individuals were identified for each patient in the OA population. The study followed the recommendations according to Strengthening the Reporting of Observational Studies in Epidemiology (STROBE).[24]

For peer review only

For peer review only - http://bmjopen.bmj.com/site/about/guidelines.xhtml

Table 1 Characteristics of the OA population from the BOA Register and the reference cohort and differences in comorbidities between the populations identified by the comorbidity indices

	**OA population** (n=72 069)	**Reference cohort** (n=216 207)
**Age** (years) mean (SD)	66.4 (9.6)	66.4 (9.6)
**Women**, % (n)	69 (49 494)	69 (148 482)
**Most affect joint in the OA population**, % (n)		
Hip	32 (22 703)	
Knee	68 (49 366)	
**Born outside Sweden**, % (n)	9 (6474)	13 (28 554)
Missing	*n=0*	*n=12*
**Married**, % (n)	59 (42 359)	54 (117 616)
Missing	*n=9*	*n=162*
**Educational level**, % (n)		
Low (≤9 years)	22.5 (16 276)	28.5 (61 212)
Medium (10-14 years)	60.5 (43 492)	56 (119 416)
High (≥ 15 years)	17 (12 111)	15.5 (33 260)
Missing	*n=190*	*n=2 319*
**Comorbidities indices**		
Missing	*n=0*	*n=0*
**RxRisk Index**,[a] median (IQR)	3 (4)	2 (4)
Min-max score	0-18	0-18
0 (no comorbidities), % (CI)[b]	16 (15.7, 16.3)	23 (22.8, 23.2)
1-3	44 (43.6, 44.4)	40.5 (40.3, 40.7)
≥4	40 (39.6, 40.4)	36.5 (36.3, 36.7)
**Elixhauser Comorbidity Index**, median (IQR)	0 (1)	0 (1)
Min-max score	0-11	0-13
0 (no comorbidities), % (CI)[b]	69.5 (69.2, 69.8)	69 (68.8, 69.2)
1	16.5 (16.2, 16.8)	16 (15.8, 16.2)
≥2	14 (13.7, 14.3)	15 (14.8, 15.2)
**Charlson Comorbidity Index**, median (IQR)	0 (0)	0 (0)
Min-max score	0-11	0-15
0 (no comorbidities), % (CI)[b]	81 (80.7, 81.3)	78 (77.8, 78.2)
1	9.5 (9.3, 9.7)	10 (9.9, 10.1)
≥2	9.5 (9.3, 9.7)	12 (11.9, 12.1)
**Comorbidities (≥1)** grouped by age, % (CI)[b]		
<45 years (OA n=1583; ref n=4749)	65 (62.7, 67.3)	51 (49.6, 52.4)
46–65 years (OA n=29 225; ref n=87 675)	79 (78.5, 79.5)	69 (68.7, 69.3)
66–80 years (OA n=36 993; ref n=110 979)	90 (89.7, 90.3)	84.5 (84.3, 84.7)
>81 years (OA n=4268; ref n=12 804)	96 (95.4, 96.6)	94 (93.6, 94.4)

SD, standard deviation; n, numbers; IQR, interquartile range; CI, confidence interval.

[a]The conditions "inflammation/pain" and "pain" are excluded from the score count.

[b]Percentage with 95% confidence interval (CI) for group comparison of proportions.

For peer review only - http://bmjopen.bmj.com/site/about/guidelines.xhtml

Data source

By using the 10-digit personal identity number (PIN), a unique number assigned to all Swedish residents at birth or immigration,[25] data on comorbidities from the index date and 3 years before that date, were obtained from two nationwide individual-based registers at the National Board of Health and Welfare: (1) the Swedish National Inpatient Register, which has an overall positive predictive value for diagnoses of 85%–95%; and (2) the Swedish Prescribed Drug Register, with data on expenditure of prescribed drugs in the entire Swedish population.[26, 27] Socioeconomic individual-level data for the same year as the index date were obtained from the TPR and the Longitudinal Integration Database for Health Insurance and Labour Market Studies (LISA) at Statistics Sweden.[23, 28] Only the PIN and data on the most affected joint (hip or knee) were extracted from the BOA Register. Details of the different registers and the linkage of data between them, together with the creation and matching of a reference cohort from the general Swedish population, as well as a flowchart of the study participants from the BOA Register and their matched references have been described previously.[22, 29]

Identification of comorbidities

Comorbidities were identified using three different comorbidity indices: (1) the RxRisk Index,[30] (2) the Charlson Comorbidity Index[31] and the Elixhauser Comorbidity Index,[32] and are presented as (1) individual diseases, (2) disease categories and (3) scores for each of the indices. Together, the three indices identified 66 individual diseases, which were grouped into 11 different disease categories (table 2).[33, 34] When the scores for the indices were calculated, the unweighted versions of all three indices were used; for example, a score of 2 indicates that the patient had two comorbidities.[30, 35, 36]

For peer review only - http://bmjopen.bmj.com/site/about/guidelines.xhtml

The RxRisk Index uses data on prescriptions of drugs to identify the presence of 43 individual diseases (table 2) based on the Anatomical Therapeutic Chemical (ATC) classification system of the drugs, defined according to the World Health Organization.[30] In this study, data from the Swedish Prescribed Drug Register were used, and the specific ATC codes for each of the 43 comorbidities are presented in Supplementary file 1. Scores for the index were categorized as 0, 1–3, or ≥4 comorbidities.

To calculate the Charlson Comorbidity Index[31] and the Elixhauser Comorbidity Index,[32] we used data on International Classification of Diseases, Tenth Revision (ICD-10) codes from the Swedish National Inpatient Register. The Charlson Comorbidity Index identifies 17 comorbidities and the Elixhauser Comorbidity Index identifies 30 comorbidities (table 2, supplementary file 2). Scores for both indices were categorized as 0, 1 or ≥2 comorbidities.

For peer review only

Table 2 Comorbidities of patients with osteoarthritis (OA) from the BOA Register, compared with the general population

			**RxRisk Index**		**Elixhauser Comorbidity Index**		**Charlson Comorbidity Index**	
	OA	Ref	OA	Ref	OA	Ref	OA	Ref
**Cancer**	**6%**	**6%**						
Lymphoma					0.5%	0.5%		
Malignancies			0.5%	0.5%			5.5%	6%
Metastatic cancer					0.5%	1%	0.5%	1%
Solid tumour without metastasis					5%	5.5%		
**Cardiovascular/blood**	**59%**	**54%**						
Anticoagulation agents/coagulopathy			10%	9%	0.5%	0.5%		
Antiplatelet agents			19%	19%				
Arrhythmias			2%	2%	6%	6%		
Cerebrovascular disease							2.5%	3.5%
Congestive heart failure			21.5%	21%	1.5%	2.5%	1.5%	2.5%
Hyperlipidaemia			28%	26.5%				
Hypertension			31%	27%	15%	14.5%		
Ischaemic heart disease: angina			7%	6.5%				
Ischaemic heart disease: hypertension			34%	32%				
Myocardial infarction							2.5%	3%
Peripheral vascular disease					1%	1.5%	1%	1.5%
Pulmonary circulation disorders					0.5%	0.5%		
Pulmonary hypertension			0%	0%				
Valvular disease					1.5%	1.5%		
**Endocrine**	**19%**	**19%**						
Diabetes (uncomplicated)			9%	10%	4.5%	5%	4.5%	5%
Diabetes (complicated)					1.5%	2%	1.5%	2%
Hyperthyroidism			0%	0%				
Hypothyroidism			10.5%	9.5%	2%	2%		
Pancreatic insufficiency			0.5%	0.5%				
**Gastrointestinal**	**35%**	**28%**						
Gastroesophageal reflux disease			32.5%	25%				
Irritable bowel syndrome			1.5%	1%				
Liver disease (mild)					0.5%	0.5%	0.5%	0.5%
Liver disease (severe) or failure			4%	4.5%			0%	0%
Peptic ulcer disease					0.5%	0.5%	0.5%	0.5%
**Musculoskeletal/pain related**	***70%***	***45%***						
**- Excl. *pain* and *inflammation/pain***	**11%**	**10%**						
Gout			2.5%	2.5%				
Migraine			2.5%	2%				
Osteoporosis/Paget's			4.5%	4.5%				
Pain			*31.5%*	*23%*				
Inflammation/pain			*58.5%*	*31.5%*				
Rheumatic disease							1.5%	2%
Rheumatoid arthritis /collage vascular diseases					2%	2.5%		

For peer review only - http://bmjopen.bmj.com/site/about/guidelines.xhtml

			RxRisk Index		Elixhauser Comorbidity Index		Charlson Comorbidity Index	
	OA	Ref	OA	Ref	OA	Ref	OA	Ref
**Neurologic**	**7%**	**8%**						
Dementia			0%	1%			0%	1%
Epilepsy			4%	4.5%				
Paralysis					0%	0.5%		
Hemiplegia or paraplegia							0%	0.5%
Parkinson's disease			2.5%	2%				
Other neurologic disorders					1%	2%		
**Nutritional/obesity**	**3%**	**3%**						
Blood loss anaemia					0%	0%		
Deficiency anaemia					0.5%	0.5%		
Fluid and electrolyte disorders					0.5%	1%		
Hyperkalaemia			0%	0%				
Malnutrition			0%	0%				
Obesity					2%	1%		
Weight loss					0%	0%		
**Psychological/behavioural**	**23%**	**24%**						
Alcohol abuse/dependency			0.5%	0.5%	0.5%	1%		
Anxiety			8.5%	10%				
Bipolar disorder			0.5%	0.5%				
Depression			17.5%	17.5%	2%	2.5%		
Drug abuse					0%	0.5%		
Psychotic illness/psychoses			0.5%	1.5%	0%	0.5%		
Smoking cessation			1.5%	1.5%				
**Renal/urologic**	**9%**	**7%**						
Benign prostatic hyperplasia			4.5%	3.5%				
Incontinence			4.5%	3%				
Renal disease/failure			0.5%	0.5%	0.5%	1%	0.5%	1%
**Respiratory**	**12%**	**11%**						
Chronic pulmonary disease					3%	3.5%	3%	3.5%
Chronic airways disease			11%	9.5%				
**Miscellaneous**	**37%**	**31%**						
Allergies			24%	19.5%				
Glaucoma			4%	4%				
HIV/AIDS			0%	0%	0%	0%	0%	0%
Psoriasis			1.5%	1.5%				
Steroid-responsive conditions			16.5%	13.5%				
Transplant			0%	0%				

Comorbidities presented as disease categories and as individual comorbidities, identified by the RxRisk Index, the Elixhauser Comorbidity Index and the Charlson Comorbidity Index, for the OA population (n= 72 069) compared with their references from the general population (n=216 207). All numbers are reported as percentages (%). The numbers marked in bold are the percentage of each population that has been identified with at least one of the individual diseases included in that disease

For peer review only - http://bmjopen.bmj.com/site/about/guidelines.xhtml

category. The other numbers are the percentage of each population that has been identified with each individual disease by each of the indices.

For peer review only

For peer review only - http://bmjopen.bmj.com/site/about/guidelines.xhtml

Indicators of socioeconomic status

Highest educational level achieved, country of birth and marital status were used as proxies for SES. Education was divided into three categories: low (≤primary school, 0–9 years), medium (secondary school plus up to <3 years postsecondary education, 10–14 years) and high (postsecondary education ≥3 years, ≥15 years). Country of birth was categorized as Sweden, the Nordic countries, Europe or others, and marital status as married (including registered partner) or not married.[29]]

Statistical analysis

Comorbidities in the OA population compared with the reference cohort are described using frequencies, proportions) or medians and interquartile ranges (IQRs) depending on the nature of the data. The OA population was also analysed separately grouped by age (<45, 46–65, 66–80 and >81 years) and by most affected joint (hip or knee) and compared with their respective matched references.

Two conditions that were identified by the RxRisk Index, inflammation/pain (treated with anti-inflammatory medication) and pain (treated with narcotics), were excluded in all calculations of the RxRisk Index, and from the analyses of the disease category, musculoskeletal and pain-related diseases, because they were considered as index diseases for this OA population.

Logistic regression models were used to evaluate the eventual effect of SES on the prevalence of comorbidities. Both crude and adjusted analyses were performed for socioeconomic indicators (educational level, country of birth and marital status), calculating the odds ratio (OR) with the 95% confidence interval (CI). For those who were included in the BOA Register up to 2015 and their matched references, we also had access to data on

For peer review only - http://bmjopen.bmj.com/site/about/guidelines.xhtml

other socioeconomic indicators (income, employment and cohabitation). Secondary analyses were therefore performed to evaluate if those indicators affected the results, but because they did not, we chose only to use those indicators for SES that we had access to for the total study population.

Some anti-inflammatory medications, such as non-steroidal anti-inflammatory drugs (NSAIDs), increase the risk of developing gastrointestinal comorbidities.[37] To prevent this, they are often prescribed in combination with gastro-protective drugs (ATC codes A02BA01–A02BX05). Because nearly two-thirds of the OA population but only one-third of their references had prescriptions for anti-inflammatory medications (ATC codes M01A–M01H), we also chose to do separate analyses of those who were not using anti-inflammatory drugs to try to establish the real prevalence of gastroesophageal diseases.

The merging of data from the National Board of Health and Welfare and the creation of the database were performed using SAS 9.4 TS Level 1MS. All statistical analyses were performed with IBM SPSS Statistics for Windows, v25.0 (IBM Statistics, Armonk, NY).

Patient and public involvement

Patients were not involved in the development of the research question or the design of this study. However, patients were involved and contributed with key content when the national first-line intervention programme for hip and knee OA was created.

Ethical approval and trial registration

The study was approved by the Regional Ethical Review Board in Gothenburg, Sweden (entry number 1059-16), and the study was registered at clinicaltrials.gov, with trial registration number NCT03438630. All patients in the BOA Register received oral and written information about their registration. Regarding the reference cohort, informed consent

For peer review only - http://bmjopen.bmj.com/site/about/guidelines.xhtml

was waived according to standard practice in Sweden, since no individuals in this population-based cohort could be identified. Statistics Sweden replaces each PIN with a serial number to anonymize data before returning it to the researchers.

RESULTS

Study participants

The characteristics of the participants in the study are described in table 1. The mean age was 66.4 years (SD, 9.6 years) and 69% were women. In the OA population, 32% were registered with OA most affecting the hip joint and 68% most affecting the knee joint.

Prevalence of comorbidities

A higher proportion of individuals in the OA population (85%) had comorbidities than in the reference cohort; 78% of the references were identified with at least one comorbidity. Of the 66 comorbidities that could be traced, 23 had a low prevalence with ≤1% in each of the populations (table 2). Cardiovascular/blood diseases were the most common comorbidities in the disease category, with 59% of this OA population and 54% of their references identified with at least one of those, followed by gastrointestinal diseases (35% of OA and 28% of the references).

In the OA population, 32.5% were identified (by the RxRisk Index) as having medication prescribed for gastroesophageal reflux disease compared with 25% of the reference cohort. Separate analyses excluding those without concomitant treatments with anti-inflammatory medications showed that the prevalence of gastroesophageal reflux disease evened out between the two populations, but was still higher in the OA population (24%) than among the references (19%).

For peer review only - http://bmjopen.bmj.com/site/about/guidelines.xhtml

A fourth of both populations had psychological/behavioural comorbidities; depression (17.5% in both populations) and anxiety (8.5% in the OA population and 10% in the reference cohort) were the most common with a similar representation in both populations (table 2). Also the prevalence of endocrine diseases such as diabetes was equally distributed in the two populations (19%).

When scores were calculated for each of the three indices, the OA population was identified with more comorbidities by the RxRisk Index, compared with their references; with the Elixhauser Comorbidity Index, the prevalence of comorbidities was similar between the two populations; and with the Charlson Comorbidity Index, the OA population showed a slightly lower prevalence of comorbidities compared with the reference cohort (table 1).

Associations between the populations and comorbidities

The OA population had a higher risk of having at least one other comorbidity (OR, 1.62; 95% CI 1.59 to 1.66), but the odds then decreased for additional comorbidities, with an OR of 1.36 (95% CI 1.34 to 1.39) for having ≥2 and 1.23 (95% CI 1.21 to 1.26) for having ≥3 comorbidities. Adjustment for SES led to an slightly higher OR overall than the crude value (table 3).

Being part of this OA population was also associated with a higher odds of having comorbidities in several of the disease categories (cardiovascular/blood, endocrine, gastrointestinal, renal/urologic, respiratory and miscellaneous), but also statistically significant lower ORs for cancer, neurologic, nutritional/obesity and psychological/behavioural diseases. The results when adjusted for SES were similar to the crude ORs (figure 1, table 3).

For peer review only

For peer review only - http://bmjopen.bmj.com/site/about/guidelines.xhtml

Table 3 Odds ratio (OR) of having comorbidities for the OA population in the BOA Register compared with the reference cohort

	**Total study population**		**Hip OA**		**Knee OA**	
	**Crude OR** (95% CI)	**Adjusted OR** (95% CI)[a]	**Crude OR** (95% CI)	**Adjusted OR** (95% CI)[a]	**Crude** (95% CI)	**Adjusted OR** (95% CI)[a]
**Individual comorbidities[b]**						
≥1	1.62 (1.59, 1.66)*	1.67 (1.63, 1.71)*	1.48 (1.42, 1.54)*	1.53 (1.46, 1.59)*	1.69 (1.65, 1.74)*	1.73 (1.69, 1.78)*
≥2	1.36 (1.34, 1.39)*	1.41 (1.38, 1.44)*	1.26 (1.22, 1.30)*	1.31 (1.27, 1.35)*	1.41 (1.38, 1.45)*	1.46 (1.43, 1.49)*
≥3	1.23 (1.21, 1.26)*	1.28 (1.26, 1.31)*	1.14 (1.11, 1.17)*	1.19 (1.16, 1.23)*	1.28 (1.25, 1.31)*	1.33 (1.30, 1.36)*
**Disease categories**						
**Cancer**	0.89 (0.86, 0.92)*	0.89 (0.85, 0.92)*	0.90 (0.85, 0.96)*	0.89 (0.84, 0.95)*	0.88 (0.84, 0.92)*	0.88 (0.84, 0.92)*
**Cardiovascular/ blood**	1.22 (1.20, 1.24)*	1.26 (1.24, 1.28)*	1.15 (1.11, 1.18)*	1.19 (1.15, 1.23)*	1.26 (1.23, 1.29)*	1.29 (1.27, 1.32)*
**Endocrine**	1.04 (1.02, 1.06)*	1.09 (1.06, 1.11)*	0.97 (0.93, 1.01)	1.02 (0.98, 1.06)	1.08 (1.05, 1.10)*	1.12 (1.09, 1.15)*
**Gastrointestinal**	1.38 (1.35, 1.40)*	1.43 (1.41, 1.46)*	1.31 (1.27, 1.35)*	1.37 (1.33, 1.42)*	1.41 (1.38, 1.44)*	1.46 (1.43, 1.50)*
**Musculoskeletal/ pain-related[c]**	1.03 (1.00, 1.06)	1.04 (1.01, 1.07)*	1.02 (0.97, 1.07)	1.02 (0.97, 1.08)	1.04 (1.00, 1.07)	1.04 (1.01, 1.08)
**Neurologic**	0.79 (0.76, 0.82)*	0.82 (0.79, 0.84)*	0.74 (0.69, 0.78)*	0.76 (0.71, 0.80)*	0.82 (0.78, 0.85)*	0.84 (0.81, 0.88)*
**Nutritional/obesity**	0.92 (0.88, 0.97)*	0.97 (0.92, 1.02)	0.75 (0.68, 0.83)*	0.79 (0.72, 0.87)*	1.01 (0.95, 1.07)	1.06 (0.99, 1.12)
**Psychological/ behavioural**	0.96 (0.94, 0.98)*	0.99 (0.97, 1.01)	0.94 (0.90, 0.97)*	0.97 (0.93, 1.00)	0.96 (0.94, 0.99)*	1.00 (0.97, 1.02)
**Renal/urologic**	1.30 (1.26, 1.34)*	1.32 (1.28, 1.36)*	1.24 (1.18, 1.31)*	1.27 (1.20, 1.34)*	1.33 (1.29, 1.38)*	1.34 (1.30, 1.40)*
**Respiratory**	1.16 (1.13, 1.19)*	1.18 (1.14, 1.21)*	1.11 (1.06, 1.16)*	1.13 (1.08, 1.18)*	1.18 (1.15, 1.22)*	1.20 (1.16, 1.24)*
**Miscellaneous**	1.29 (1.27, 1.31)*	1.29 (1.27, 1.31)*	1.23 (1.19, 1.27)*	1.22 (1.19, 1.26)*	1.32 (1.29, 1.35)*	1.32 (1.29, 1.35)*

OA, osteoarthritis; OR, odds ratio; CI, confidence interval.

[a]Adjusted for socioeconomic status (educational level, country of birth and marital status).

[b]Individual comorbidities identified by summing the RxRisk Index, the Elixhauser Comorbidity Index and the Charlson Comorbidity Index.

For peer review only

[c]The conditions inflammation/pain and pain identified by the Rx Risk Index were excluded from the analyses.

*Statistically significant results.

Separate analyses of subgroups

Grouping the populations by age, the OA population had a higher presence of comorbidities (≥1) in the younger age groups compared with their matched references, whereas in the oldest age group, the prevalence were similar in the two populations (table 1). The OA population also showed an overall higher odds of having one or more comorbidities for all age groups, the younger, the higher ORs. There were no differences in OR for ≥1, ≥2 or ≥3 comorbidities in the youngest age group (aged <45 years). In contrast, the ORs for ≥2 or ≥3 comorbidities were continuously lower than having only ≥1 comorbidity in the older age groups (aged 46–>81 years) (figure 2).

When grouping the OA population by most affected joint (hip/knee) and then comparing them with their respective matched references, those registered with knee OA had more comorbidities than those registered with hip OA (table 3). Also in the separate analyses of the populations in subgroups, adjustment for SES had only a minor effect on the estimates (figure 2, table 3).

DISCUSSION

This study shows that individuals referred to a standardized national first-line intervention for OA in Sweden had an overall higher prevalence of comorbidities than their matched references from the general population. The study also shows that there was a higher prevalence of comorbidities among younger individuals in the OA population compared with their references, whereas the prevalence was similar for older individuals, which could indicate that it is probably important to initiate first-line intervention early to reduce the risk

For peer review only - http://bmjopen.bmj.com/site/about/guidelines.xhtml

of developing related disease.[17] Patients in the OA population registered with knee OA had more comorbidities than those registered with hip OA. This may be partly explained by the fact that obesity is more common among those registered with knee OA compared with hip OA in the BOA Register,[18] which is also a risk factor for the development of several other diseases.[6, 7]

Previously, we have shown an overall higher SES in this OA population from the BOA Register compared with that of the general Swedish population.[29] From the results in the present study, we can now report that the inequalities in SES were not associated with differences in comorbidities, but must be related to other factors. It is well known that there is a connection between SES and several common chronic diseases, with poorer health among more socioeconomically disadvantaged individuals,[38, 39] and that OA is in general more common among individuals with lower SES.[40, 41] The reasons why this OA population had a higher SES, but had more comorbidities than the general population, are probably highly dependent on the association that exists between OA and other diseases.[5] In addition, it could also be the effect of individuals' lifestyle and health care seeking behaviours; individuals with lower SES tends to seek health care less often, which could result in both late diagnosis and delayed treatment.[42] It has also been shown previously that more disadvantaged individuals with OA have poorer access to both nonsurgical health care services, such as self-management education, physiotherapy and medication, and to joint replacement surgery.[43, 44] In the present study, the OA population showed a higher prevalence of comorbidities compared with the reference cohort, and most comorbidities for both populations were detected from data on prescriptions of drugs and by the RxRisk Index, whereas data from ICD-10 codes for inpatient care showed slightly more comorbidities among the reference cohort. It could be argued that the BOA population probably to a higher extent includes individuals who take responsibility for their health, who seek help for their

For peer review only - http://bmjopen.bmj.com/site/about/guidelines.xhtml

OA problems and at the right level of care, and who take the medications that are prescribed for them, and that they are thus also more represented and appear in registers that include prescription of drugs.

The differences in comorbidities between the populations in the present study were not of the same magnitude as in a recent review by Swain et al.[5] They reported that 67% of individuals with OA were identified with ≥1 other chronic condition, which was 20% higher than among non-OA individuals. Pihl et al.[45] identified self-reported comorbidities among approximate 50% of a similar Danish OA population, before participation in a first-line intervention programme for hip and knee OA, and that the presence of comorbidities were not associated with outcomes after the intervention. However, differences in prevalence of comorbidities between studies may be due to several factors, making comparison challenging, such as different study designs, the sample characteristics, methods of identifying comorbidities and the number of comorbidities analysed. Instead of comparing the overall prevalence of comorbidities in this OA population with results from other studies, it may be more interesting to compare prevalence divided into different groups of diseases. In the present study, we identified that cardiovascular/blood diseases was the most common disease category in both populations, with a higher prevalence in the OA population compared with the reference cohort. These results are supported in a previous review by Hall et al.,[46] who established that individuals with OA had a significantly higher prevalence of cardiovascular diseases, especially heart failure and ischaemic heart disease, compared with matched controls. The prevalence of diabetes[47] and depression[8] has also been shown to be higher in OA populations compared with general populations. Those results were not supported by this study; we instead identified similar prevalence in both populations. Our separate analyses excluding individuals with an anti-inflammatory prescription showed that the difference in the prevalence of gastroesophageal reflux disease was reduced but remained higher in this OA

For peer review only - http://bmjopen.bmj.com/site/about/guidelines.xhtml

population. This may be due to greater use of over-the-counter anti-inflammatory medication, which can be assumed to be higher among people with OA. The odds of having cancer or neurologic diseases was lower in the OA population than the reference cohort, but those results are probably the effect of the criteria for registration in the BOA Register, which excludes individuals with OA who also have other severe diseases.[17]

In the present study, we used three comorbidity indices to make it possible to identify more diseases, and from several sources. The Charlson Comorbidity Index and the Elixhauser Comorbidity Index were developed to capture comorbidities in inpatient care using diagnostic ICD-10 codes,[35] and are commonly used in orthopaedics research,[33] whereas the RxRisk Index identifies comorbidities from prescription of drugs by ATC codes and can be used in predominately outpatient settings,[30] such as this OA population. All three indices have used previously to study comorbidities in OA populations.[33, 34] The possibility to use comorbidity indices and summarize them into scores is necessary when handling large administrative datasets, but there is no gold standard with regard to which index should be used primarily. In the present study, we chose to combine the three indices to capture the overall burden of the comorbidities, instead of focusing on separate scores for each index, because some comorbidities are better detected using medication-based data and others with diagnosis-based data.[33, 34]

Limitations and strengths

There are some limitations in the present study that should be highlighted. We were only able to capture ICD-10 codes to calculate the Charlson Comorbidity Index and the Elixhauser Comorbidity Index from health care delivered through inpatient care and specialized outpatient visits. Data on primary health care and information on health care provided by professionals other than physicians are not available on a national level in Sweden. However,

For peer review only - http://bmjopen.bmj.com/site/about/guidelines.xhtml

both the Charlson Comorbidity Index and the Elixhauser Comorbidity Index were developed primarily to capture comorbidities in inpatient care.[35]

Another limitation to bear in mind when interpreting the results from the present study is that the criteria for registration in the BOA Register excludes individuals who have other severe diseases.[17] This may contribute to underestimating comorbidities, because we can assume that this OA population probably has less comorbidities than general OA populations who are at the same stage of their disease, when seeking health care and structured OA treatments, such as the OA population in the present study. In addition, there could be individuals with OA in the reference cohort; OA is common in those age groups, it is difficult to define the onset of the disease,[1] many individuals with OA symptoms do not seek health care[48] or are not referred to first-line intervention for OA. For example, it has been estimated that during 2014, the BOA Register reached 17% of all individuals who sought outpatient care with the diagnosis of hip or knee OA.[49]

Despite these circumstances, we could identify a higher prevalence of several diseases in the OA population compared with the age-, sex- and residence-matched reference cohort. The results from the present study are in line with several previous studies,[5, 6, 46] describing comorbidities among individuals with OA compared with general populations. Even though more recent guidelines on OA treatment include recommendations for individuals with comorbidities,[15] they are often excluded from exercise treatment,[50] with the risk of being left without any treatment. However, OA is not a single-joint disease and to improve treatment outcomes from first-line intervention for hip and knee OA, it is important to not only focus on specific OA treatments but also on increased overall health.

For peer review only - http://bmjopen.bmj.com/site/about/guidelines.xhtml

CONCLUSION

Comorbidities were more common among individuals referred to first-line intervention for hip and knee OA than among a matched reference cohort from the general population, especially among younger individuals and those with OA most affecting their knees. Based on the results from this study, socioeconomic status does not seem to affect the differences in comorbidities detected between this OA population and the reference cohort.

ACKNOWLEDGEMENTS

The authors would like to acknowledge all participating patients, physiotherapists reporting data to the BOA Register, and others involved in BOA.

Competing interests

LED is the co-founder and Chief Medical Officer of Joint Academy, a company that provides digital first-line intervention for patients with hip and knee OA. The other authors declare that they have no conflicts of interest.

Author contributions

KG, JK, ME, and OR designed the study. KG and OR contributed to acquisition of data. CZ were responsible for merging of data from the BOA Register with data from the National Board of Health and Welfare and Statistics Sweden and for the creation of the research database. KG had full access to all data that was analysed in the study and takes, together with CZ, responsibility for the accuracy of the data analysis. All authors contributed in interpretation of data. KG wrote the first draft of the paper, which was critically revised by the other authors. All authors interpreted the findings and approved the final version of the manuscript before submission.

For peer review only - http://bmjopen.bmj.com/site/about/guidelines.xhtml

Funding statement

The study was financially supported by AFA Insurance, Sweden, Futurum–Academy for Health and Care, Region Jönköping County, Sweden and the Medical Research Council of Southeast Sweden. The funders had no influence on the design or the conduct of the study, the analysis or interpretation of the data or the writing of the manuscript.

Data sharing statement

The dataset supporting the conclusions of this article is governed by Västra Götalandsregionen. The authors are not allowed to share the data. The data are available from Registercentrum, Västra Götalandsregionen (contact; boa@registercentrum.se) for researchers who meet the criteria for access to confidential data according to Swedish law.

REFERENCES

1 Woolf AD, Pfleger B. Burden of major musculoskeletal conditions. *Bull World Health Organ* 2003;81(9):646-56.

2 Turkiewicz A, Petersson IF, Bjork J, et al. Current and future impact of osteoarthritis on health care: a population-based study with projections to year 2032. *Osteoarthritis Cartilage* 2014;22(11):1826-32.

3 Cross M, Smith E, Hoy D, et al. The global burden of hip and knee osteoarthritis: estimates from the global burden of disease 2010 study. *Ann Rheum Dis* 2014;73(7):1323-30.

4 Roos EM, Arden NK. Strategies for the prevention of knee osteoarthritis. *Nat Rev Rheumatol* 2016;12(2):92-101.

5 Swain S, Sarmanova A, Coupland C, et al. Comorbidities in Osteoarthritis: A Systematic Review and Meta‐Analysis of Observational Studies. *Arthritis Care & Research* 2020;72(7):991-1000.

For peer review only - http://bmjopen.bmj.com/site/about/guidelines.xhtml

6 Kadam UT, Jordan K, Croft PR. Clinical comorbidity in patients with osteoarthritis: a case-control study of general practice consulters in England and Wales. *Ann Rheum Dis* 2004;63(4):408-14.

7 Violan C, Foguet-Boreu Q, Flores-Mateo G, et al. Prevalence, determinants and patterns of multimorbidity in primary care: a systematic review of observational studies. *PLoS One* 2014;9(7):e102149.

8 Veronese N, Stubbs B, Solmi M, et al. Association between lower limb osteoarthritis and incidence of depressive symptoms: data from the osteoarthritis initiative. *Age Ageing* 2017;46(3):470-76.

9 Wilkie R, Kaur K, Hayward RA. The impact of socioeconomic status on the link between osteoarthritis and the onset of common comorbidities. *Clin Exp Rheumatol* 2019;37(1):44-48.

10 Smith SM, Soubhi H, Fortin M, et al. Managing patients with multimorbidity: systematic review of interventions in primary care and community settings. *Bmj* 2012;345:e5205.

11 Calders P, Van Ginckel A. Presence of comorbidities and prognosis of clinical symptoms in knee and/or hip osteoarthritis: A systematic review and meta-analysis. *Semin Arthritis Rheum* 2018;47(6):805-13.

12 Geryk LL, Carpenter DM, Blalock SJ, et al. The impact of co-morbidity on health-related quality of life in rheumatoid arthritis and osteoarthritis patients. *Clin Exp Rheumatol* 2015;33(3):366-74.

13 Verhagen AP, Ferreira M, Reijneveld-van de Vendel EAE, et al. Do we need another trial on exercise in patients with knee osteoarthritis?: No new trials on exercise in knee OA. *Osteoarthritis Cartilage* 2019;27(9):1266-69.

14 Kolasinski SL, Neogi T, Hochberg MC, et al. 2019 American College of Rheumatology/Arthritis Foundation Guideline for the Management of Osteoarthritis of the Hand, Hip, and Knee. *Arthritis Rheumatol* 2020;72(2):220-33.

For peer review only - http://bmjopen.bmj.com/site/about/guidelines.xhtml

15 Bannuru RR, Osani MC, Vaysbrot EE, et al. OARSI guidelines for the non-surgical management of knee, hip, and polyarticular osteoarthritis. *Osteoarthritis Cartilage* 2019;27(11):1578-89.

16 Zhang W, Nuki G, Moskowitz RW, et al. OARSI recommendations for the management of hip and knee osteoarthritis: part III: Changes in evidence following systematic cumulative update of research published through January 2009. *Osteoarthritis Cartilage* 2010;18(4):476-99.

17 Thorstensson CA, Garellick G, Rystedt H, et al. Better Management of Patients with Osteoarthritis: Development and Nationwide Implementation of an Evidence-Based Supported Osteoarthritis Self-Management Programme. *Musculoskeletal Care* 2015;13(2):67-75.

18 Jonsson T, Eek F, Dell'Isola A, et al. The Better Management of Patients with Osteoarthritis Program: Outcomes after evidence-based education and exercise delivered nationwide in Sweden. *PLoS One* 2019;14(9):e0222657.

19 Altman R, Alarcon G, Appelrouth D, et al. The American College of Rheumatology criteria for the classification and reporting of osteoarthritis of the hip. *Arthritis Rheum* 1991;34(5):505-14.

20 Altman R, Asch E, Bloch D, et al. Development of criteria for the classification and reporting of osteoarthritis. Classification of osteoarthritis of the knee. Diagnostic and Therapeutic Criteria Committee of the American Rheumatism Association. *Arthritis Rheum* 1986;29(8):1039-49.

21 Socialstyrelsen. Nationella riktlinjer för rörelseorganens sjukdomar 2012: Osteoporos, artros, inflammatoriskryggsjukdom och ankyloserande spondylit, psoriasisartrit och reumatoid artrit: Stöd för styrning och ledning [Swedish]. Stockholm; Socialstyrelsen; 2012.

22 Gustafsson K, Rolfson O, Eriksson M, et al. Study protocol for an observational register-based study on health and risk factors in patients with hip and knee osteoarthritis. *BMJ Open* 2018;8(10):e022812.

For peer review only

23 Ludvigsson JF, Almqvist C, Bonamy AK, et al. Registers of the Swedish total population and their use in medical research. *Eur J Epidemiol* 2016;31(2):125-36.

24 von Elm E, Altman DG, Egger M, et al. The Strengthening the Reporting of Observational Studies in Epidemiology (STROBE) Statement: guidelines for reporting observational studies. *Int J Surg* 2014;12(12):1495-9.

25 Ludvigsson JF, Otterblad-Olausson P, Pettersson BU, et al. The Swedish personal identity number: possibilities and pitfalls in healthcare and medical research. *Eur J Epidemiol* 2009;24(11):659-67.

26 Ludvigsson JF, Andersson E, Ekbom A, et al. External review and validation of the Swedish national inpatient register. *BMC Public Health* 2011;11:450.

27 Wallerstedt SM, Wettermark B, Hoffmann M. The First Decade with the Swedish Prescribed Drug Register - A Systematic Review of the Output in the Scientific Literature. *Basic Clin Pharmacol Toxicol* 2016;119(5):464-69.

28 Statistics Sweden. Longitudinal integration database for health insurance and labour market studies (LISA). 2018 [cited 2019 17 September]. Available from: https://www.scb.se/vara-tjanster/bestalla-mikrodata/vilka-mikrodata-finns/longitudinella-register/longitudinell-integrationsdatabas-for-sjukforsakrings--och-arbetsmarknadsstudier-lisa/ accessed 13 January 2021.

29 Gustafsson K, Kvist J, Eriksson M, et al. Socioeconomic status of patients in a Swedish national self-management program for osteoarthritis compared with the general population-a descriptive observational study. *BMC Musculoskelet Disord* 2020;21(1):10.

30 Pratt NL, Kerr M, Barratt JD, et al. The validity of the Rx-Risk Comorbidity Index using medicines mapped to the Anatomical Therapeutic Chemical (ATC) Classification System. *BMJ Open* 2018;8(4):e021122.

31 Charlson ME, Pompei P, Ales KL, et al. A new method of classifying prognostic comorbidity in longitudinal studies: development and validation. *J Chronic Dis* 1987;40(5):373-83.

For peer review only - http://bmjopen.bmj.com/site/about/guidelines.xhtml

32 Elixhauser A, Steiner C, Harris DR, et al. Comorbidity measures for use with administrative data. *Med Care* 1998;36(1):8-27.

33 Inacio MC, Pratt NL, Roughead EE, et al. Comparing co-morbidities in total joint arthroplasty patients using the RxRisk-V, Elixhauser, and Charlson Measures: a cross-sectional evaluation. *BMC Musculoskelet Disord* 2015;16:385.

34 Dominick KL, Dudley TK, Coffman CJ, et al. Comparison of three comorbidity measures for predicting health service use in patients with osteoarthritis. *Arthritis Rheum* 2005;53(5):666-72.

35 Quan H, Sundararajan V, Halfon P, et al. Coding algorithms for defining comorbidities in ICD-9-CM and ICD-10 administrative data. *Med Care* 2005;43(11):1130-9.

36 Molto A, Dougados M. Comorbidity indices. *Clin Exp Rheumatol* 2014;32(5 Suppl 85):S-131-4.

37 Lanza FL, Chan FKL, Quigley EMM, et al. Guidelines for Prevention of NSAID-Related Ulcer Complications. *Official journal of the American College of Gastroenterology | ACG* 2009;104(3):728-38.

38 Adler NE, Ostrove JM. Socioeconomic status and health: what we know and what we don't. *Ann N Y Acad Sci* 1999;896:3-15.

39 Braveman P, Gottlieb L. The social determinants of health: it's time to consider the causes of the causes. *Public Health Rep* 2014;129 (Suppl 2):19-31.

40 Reyes C, Garcia-Gil M, Elorza JM, et al. Socio-economic status and the risk of developing hand, hip or knee osteoarthritis: a region-wide ecological study. *Osteoarthritis Cartilage* 2015;23(8):1323-9.

41 Callahan LF, Shreffler J, Siaton BC, et al. Limited educational attainment and radiographic and symptomatic knee osteoarthritis: a cross-sectional analysis using data from the Johnston County (North Carolina) Osteoarthritis Project. *Arthritis Res Ther* 2010;12(2):R46.

For peer review only

For peer review only - http://bmjopen.bmj.com/site/about/guidelines.xhtml

42 Agerholm J, Bruce D, Ponce de Leon A, et al. Socioeconomic differences in healthcare utilization, with and without adjustment for need: an example from Stockholm, Sweden. *Scand J Public Health* 2013;41(3):318-25.

43 Ackerman IN, Busija L. Access to self-management education, conservative treatment and surgery for arthritis according to socioeconomic status. *Best Pract Res Clin Rheumatol* 2012;26(5):561-83.

44 Wetterholm M, Turkiewicz A, Stigmar K, et al. The rate of joint replacement in osteoarthritis depends on the patient's socioeconomic status. *Acta Orthop* 2016;87(3):245-51.

45 Pihl K, Roos EM, Taylor RS, et al. Associations between comorbidities and immediate and one-year outcomes following supervised exercise therapy and patient education - A cohort study of 24,513 individuals with knee or hip osteoarthritis. *Osteoarthritis Cartilage* 2021;29(1):39-49.

46 Hall AJ, Stubbs B, Mamas MA, et al. Association between osteoarthritis and cardiovascular disease: Systematic review and meta-analysis. *Eur J Prev Cardiol* 2016;23(9):938-46.

47 Louati K, Vidal C, Berenbaum F, et al. Association between diabetes mellitus and osteoarthritis: systematic literature review and meta-analysis. *RMD Open* 2015;1(1):e000077.

48 Thorstensson CA, Gooberman-Hill R, Adamson J, et al. Help-seeking behaviour among people living with chronic hip or knee pain in the community. *BMC Musculoskelet Disord* 2009;10:153.

49 Thorstensson CA, Dahlberg L, Garellick G. The BOA Register Annual Report 2014. Göteborg; 2015.

50 de Rooij M, van der Leeden M, Cheung J, et al. Efficacy of Tailored Exercise Therapy on Physical Functioning in Patients With Knee Osteoarthritis and Comorbidity: A Randomized Controlled Trial. *Arthritis Care Res (Hoboken)* 2017;69(6):807-16.

For peer review only - http://bmjopen.bmj.com/site/about/guidelines.xhtml

Figure 1 Forest plot with odds ratio (OR) and 95% confidence interval for having comorbidities for the OA population compared with the reference cohort, grouped in disease categories. Reported in crude OR, and in OR adjusted for socioeconomic status (educational level, country of birth and marital status). *The conditions inflammation/pain and pain, identified by the RxRisk Index, were excluded from the analyses.

Figure 2 Forest plot with odds ratio (OR) and 95% confidence interval for having ≥1 comorbidities for the OA population compared with the reference cohort, grouped by age. Reported in crude OR, and in OR adjusted for socioeconomic status (educational level, country of birth and marital status).

For peer review only

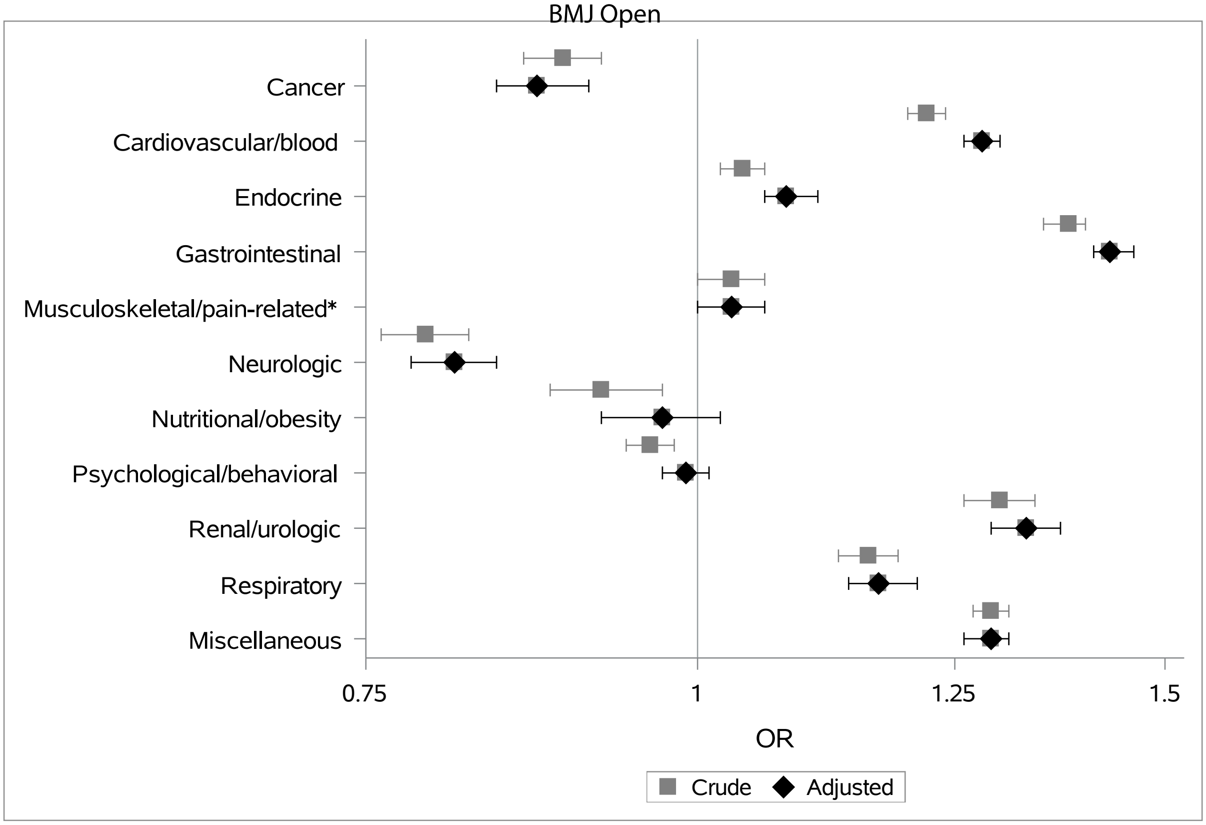
Cancer
Cardiovascular/blood
Endocrine
Gastrointestinal
Musculoskeletal/pain-related*
Neurologic
Nutritional/obesity
Psychological/behavioral
Renal/urologic
Respiratory
Miscellaneous
0.75
1
1.25
1.5
OR
Crude
Adjusted

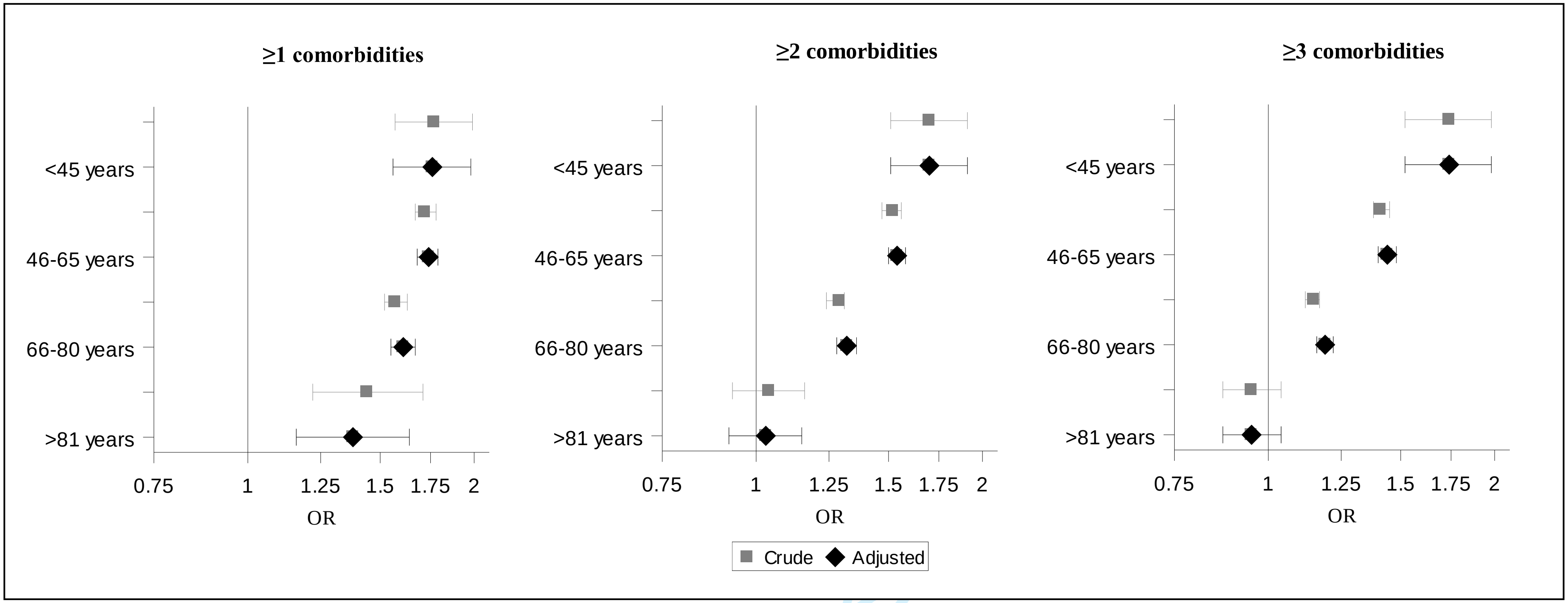

For peer review only - http://bmjopen.bmj.com/site/about/guidelines.xhtml

Supplementary file 1. ATC codes used to identify comorbidities with the RxRisk Index[1]

Comorbidities	ATC codes
Alcohol dependency	N07BB01–N07BB99
Allergies	R01AC01–R01AD60, R06AD02–R06AX27, R06AB04
Anticoagulants	B01AA03–B01AB06, B01AE07, B01AF01, B01AF02, B01AX05
Antiplatelets	B01AC04–B01AC30
Anxiety	N05BA01–N05BA12, N05BE01
Arrhythmia	C01AA05, C01BA01–C01BD01, C07AA07
Benign prostatic hyperplasia	G04CA01–G04CA99, G04CB01, G04CB02*
Bipolar disorder	N05AN01
Chronic airways disease	R03AC02–R03DC03, R03DX05
Congestive heart failure	C03DA02–C03DA99, C07AB02, C07AB07, C07AG02, C07AB12, C03DA04 (C03CA01– C03CC01 and C09AA01–C09AX99, C09CA01– C09CX99)†
Dementia	N06DA02–N06DA04, N06DX01
Depression	N06AA01–N06AG02, N06AX03–N06AX11, N06AX13–N06AX18, N06AX21–N06AX26
Diabetes	A10AA01-A10BX99
Epilepsy	N03AA01–N03AX99
Glaucoma	S01EA01–S01EB03, S01EC03–S01EX99
Gastroesophageal reflux disease	A02BA01–A02BX05
Gout	M04AA01–M04AC01
HIV	J05AE01–J05AE10, J05AF12–J05AG05, J05AR01–J05AR99, J05AX07–J05AX09, J05AX12, J05AF01–J05AF07, J05AF09
Hyperkalaemia	V03AE01
Hyperlipidaemia	C10AA01–C10BX09
Hypertension	C03AA01–C03BA11, C03DB01, C03DB99, C03EA01, C09BA02–C09BA09, C09DA02– C09DA08, C02AB01–C02AC05, C02DB02– C02DB99 (C03CA01–C03CCO1 or C09CA01– C09CX99)§
Hyperthyroidism	H03BA02, H03BB01
Hypothyroidism	H03AA01–H03AA02
Irritable bowel syndrome	A07EC01–A07EC04, A07EA01–A07EA02, A07EA06, L04AA33
Ischaemic heart disease: angina	C01DA02–C01DA14, C01DX16, C08EX02
Ischaemic heart disease: hypertension	C07AA01–C07AA06, C07AA08–C07AB01, C07AB02, C07AG01, C08CA01–C08DB01, C09DB01–C09DB04, C09DX01, C09BB02– C09BB10, C07AB03, C09DX03
Incontinence	G04BD01–G04BD99
Inflammation/pain	M01AB01–M01AH06
Liver failure	A06AD11, A07AA11
Malignancies	L01AA01–L01XX41
Malnutrition	B05BA01–B05BA10
Migraine	N02CA01–N02CX01
Osteoporosis/Paget's	M05BA01–M05BB05, M05BX03, M05BX04, G03XC01, H05AA02
Pain	N02AA01–N02AX02, N02AX06, N02AX52, N02BE51

For peer review only

Pancreatic insufficiency	A09AA02
Parkinson's disease	N04AA01–N04BX02
Psoriasis	D05AA01–D05AA99, D05BB01, D05BB02, D05AX02, D05AC01–D05AC51, D05AX52
Psychotic illness	N05AA01–N05AB02, N05AB06–N05AL07, N05AX07–N05AX13
Pulmonary hypertension	C02KX01–C02KX05
Renal disease	B03XA01–B03XA03, A11CC01–A11CC04, V03AE02, V03AE03, V03AE05
Smoking cessation	N07BA01–N07BA03, N06AX12
Steroid-responsive disease	H02AB01–H02AB10
Transplant	L04AA06, L04AA10, L04AA18, L04AD01, L04AD02

1. Pratt NL, Kerr M, Barratt JD, et al. The validity of the Rx-Risk Comorbidity Index using medicines mapped to the Anatomical Therapeutic Chemical (ATC) Classification System. *BMJ Open* 2018;8(4):e021122.

ATC code: Anatomical Therapeutic Chemical code
*Benign prostatic hyperplasia medicines – must be male.
†Must have at least two medicines prescribed with one of those medicines having an ATC code from C03CA01–C03CC01 and the other having an ATC code from either C09AA01–C09AX99 or C09CA01–C09CX99, at some point within the period of three years prior to baseline
§Can have medicine dispensed with an ATC code C03CA01–C03CC01 or C09AA01–C09AX99, but not both, as this would indicate chronic heart failure.

For peer review only

Supplementary file 2. ICD-10 codes used to identify comorbidities with the Charlson Comorbidity Index and the Elixhauser Comorbidity Index[1]

Comorbidities	ICD-10	
	Charlson Comorbidity Index	Elixhauser Comorbidity Index
AIDS/HIV	B20.x–B22.x, B24.x	B20.x–B22.x, B24.x
Alcohol abuse	-	F10, E52, G62.1, I42.6, K29.2, K70.0, K70.3, K70.9, T51.x, Z50.2, Z71.4, Z72.1
Cardiac arrhythmia	-	I44.1–I44.3, I45.6, I45.9, I47.x–I49.x, R00.0, R00.1, R00.8, T82.1, Z45.0, Z95.0
Blood loss anaemia	-	D50.0
Cerebrovascular disease	G45.x, G46.x, H34.0, I60.x-I69.x	-
Chronic pulmonary disease	I27.8, I27.9, J40.x–J47.x, J60.x–J67.x, J68.4, J70.1, J70.3	I27.8, I27.9, J40.x–J47.x, J60.x–J67.x, J68.4, J70.1, J70.3
Coagulopathy	-	D65–D68.x, D69.1, D69.3–D69.6
Congestive heart failure	I09.9, I11.0, I13.0, I13.2, I25.5, I42.0, I42.5–I42.9, I43.x, I50.x, P29.0	I09.9, I11.0, I13.0, I13.2, I25.5, I42.0, I42.5–I42.9, I43.x, I50.x, P29.0
Deficiency anaemia	-	D50.8, D50.9, D51.x–D53.x
Dementia	F00.x–F03.x, F05.1, G30.x, G31.1	-
Depression	-	F20.4, F31.3–F31.5, F32.x, F33.x, F34.1, F41.2, F43.2
Diabetes (uncomplicated)	E10.0, E10.1, E10.6, E10.8, E10.9, E11.0, E11.1, E11.6, E11.8, E11.9, E12.0, E12.1, E12.6, E12.8, E12.9, E13.0, E13.1, E13.6, E13.8, E13.9, E14.0, E14.1, E14.6, E14.8, E14.9	E10.0, E10.1, E10.9, E11.0, E11.1, E11.9, E12.0, E12.1, E12.9, E13.0, E13.1, E13.9, E14.0, E14.1, E14.9
Diabetes (complicated)	E10.2–E10.5, E10.7, E11.2–E11.5, E11.7, E12.2–E12.5, E12.7, E13.2–E13.5, E13.7, E14.2–E14.5, E14.7	E10.2–E10.8, E11.2–E11.8, E12.2–E12.8, E13.2–E13.8, E14.2–E14.8
Drug abuse	-	F11.x–F16.x, F18.x, F19.x, Z71.5, Z72.2
Fluid and electrolyte disorders	-	E22.2, E86.x, E87.x
Hemiplegia or paraplegia	G04.1, G11.4, G80.1, G80.2, G81.x, G82.x, G83.0–G83.4, G83.9	-
Hypertension[a]	-	I10.x, I11.x–I13.x, I15.x
Hypothyroidism	-	E00.x–E03.x, E89.0
Liver disease	B18.x, K70.0–K70.3, K70.9, K71.3–K71.5, K71.7, K73.x, K74.x, K76.0, K76.2–K76.4, K76.8, K76.9, Z94.4	B18.x, I85.x, I86.4, I98.2, K 70.x, K71.1, K71.3–K71.5, K71.7, K72.x–K74.x, K76.0, K76.2–K76.9, Z94.4
Liver failure	I85.0, I85.9, I86.4, I98.2, K70.4, K71.1, K72.1, K72.9, K76.5, K76.6, K76.7	-
Lymphoma	-	C81.x–C85.x, C88.x, C96.x, C90.0, C90.2
Malignancies	C00.x–C26.x, C30.x–C34.x, C37.x–C41.x, C43.x, C45.x–C58.x, C60.x–C76.x, C81.x–C85.x, C88.x, C90.x–C97.x	-
Metastatic cancer	C77.x–C80.x	C77.x–C80.x
Myocardial infarction	I21.x, I22.x, I25.2	-
Obesity	-	E66.x
Other neurologic disorders	-	G10.x–G13.x, G20.x–G22.x, G25.4, G25.5, G31.2, G31.8, G31.9, G32.x, G35.x–G37.x, G40.x, G41.x, G93.1, G93.4, R47.0, R56.x
Paralysis	-	G04.1, G11.4, G80.1, G80.2, G81.x, G82.x, G83.0–G83.4, G83.9
Peptic ulcer disease	K25.x–K28.x	K25.7, K25.9, K26.7, K26.9, K27.7, K27.9, K28.7, K28.9
Peripheral vascular disease	I70.x, I71.x, I73.1, I73.8, I73.9, I77.1, I79.0, I79.2, K55.1, K55.8, K55.9, Z95.8, Z95.9	I70.x, I71.x, I73.1, I73.8, I73.9, I77.1, I79.0, I79.2, K55.1, K55.8, K55.9, Z95.8, Z95.9
Pulmonary circulation disorders	-	I26.x, I27.x, I28.0, I28.8, I28.9
Psychoses	-	F20.x, F22.x–F25.x, F28.x, F29.x, F30.2, F31.2, F31.5

Renal disease	I12.0, I13.1, N03.2–N03.7, N05.2–N05.7, N18.x, N19.x, N25.0, Z49.0–Z49.2, Z94.0, Z99.2	I12.0, I13.1, N18.x, N19.x, N25.0, Z49.0–Z49.2, Z94.0, Z99.2
Rheumatoid arthritis/collage vascular diseases		L94.0, L94.1, L94.3, M05.x, M06.x, M08.x, M12.0, M12.3, M30.x, M31.0–M31.3, M32.x–M35.x, M45.x, M46.1, M46.8, M46.9
Rheumatic disease	M05.x, M06.x, M31.5, M32.x–M34.x, M35.1, M35.3, M36.0	
Solid tumour without metastasis		C00.x–C26.x, C30.x–C34.x, C37.x–C41.x, C43.x, C45.x–C58.x, C60.x–C76.x, C97.x
Valvular disease		A52.0, I05.x–I08.x, I09.1, I09.8, I34.x–I39.x, Q23.0–Q23.3, Z95.2–Z95.4
Weight loss	-	E40.x–E46.x, R63.4, R64

1. Quan H, Sundararajan V, Halfon P, et al. Coding algorithms for defining comorbidities in ICD-9-CM and ICD-10 administrative data. *Med Care* 2005;43(11):1130-9.

[a]Complicated and uncomplicated combined

For peer review only

Open access Protocol

BMJ Open

Study protocol for an observational register-based study on health and risk factors in patients with hip and knee osteoarthritis

Kristin Gustafsson,[1,2] Ola Rolfson,[3] Marit Eriksson,[4] Leif Dahlberg,[5] Joanna Kvist[2,6]

To cite: Gustafsson K, Rolfson O, Eriksson M, *et al*. Study protocol for an observational register-based study on health and risk factors in patients with hip and knee osteoarthritis. *BMJ Open* 2018;**8**:e022812. doi:10.1136/bmjopen-2018-022812

► Prepublication history for this paper is available online. To view these files, please visit the journal online (http://dx.doi.org/10.1136/bmjopen-2018-022812).

Received 11 March 2018
Revised 28 June 2018
Accepted 31 July 2018

Check for updates

© Author(s) (or their employer(s)) 2018. Re-use permitted under CC BY-NC. No commercial re-use. See rights and permissions. Published by BMJ.

For numbered affiliations see end of article.

Correspondence to
Kristin Gustafsson;
kristin.gustafsson@rjl.se

ABSTRACT

Introduction Hip and knee osteoarthritis is a leading cause of disability worldwide. Currently, the course of deterioration in pain and physical functioning in individuals with osteoarthritis is difficult to predict. Factors such as socioeconomic status and comorbidity contribute to progression of osteoarthritis, but clear associations have not been established. There is a need for early identification of individuals with slow disease development and a good prognosis, and those that should be recommended for future joint replacement surgery.

Methods and analysis This nationwide register-based study will use data for approximately 75 000 patients who sought and received core treatment for osteoarthritis in primary healthcare, and were registered in the Swedish population-based National Quality Register for Better Management of Patients with Osteoarthritis. These data will be merged with data for replacement surgery, socioeconomic factors, healthcare consumption and comorbidity from the Swedish Hip Arthroplasty Register, the Swedish Knee Arthroplasty Register, Statistics Sweden and the National Board of Health and Welfare, Sweden. The linkage will be performed using personal identity numbers that are unique to all citizens in Sweden.

Ethics and dissemination The study was approved by the Regional Ethical Review Board in Gothenburg, Sweden (dnr 1059–16). The results from this study will be submitted to peer-reviewed journals and reported at suitable national and international meetings.

Trial registration number NCT03438630.

Strengths and limitations of this study

- This study will use a nationwide population-based cohort to capture data for socioeconomic factors, comorbidity and patient-reported measures that affect individuals with osteoarthritis.
- This study will include a reference cohort from the general Swedish population, matched by year of birth, sex and residence.
- The study design will allow investigation of changes over time in the same individual and the progression of osteoarthritis to be followed.
- A limitation of register-based studies is that the data to be collected are predetermined, and it is difficult to add other individual data for study purposes.

INTRODUCTION

Osteoarthritis (OA) is the most common joint disease and has a major public health impact.[1] In Sweden, one in four people aged over 45 years suffers from OA in some joint.[2] The disease develops slowly over time and leads to joint stiffness, gradually increased pain and difficulty performing activities of daily living.[2–4] Diagnosis is based on clinical criteria, with radiographs only needed in uncertain diagnostic cases or when surgical interventions are planned.[5–7] Radiographic findings often correlate poorly with patients' symptoms, and awaiting radiography may also delay treatment early in the disease.[8 9] Today, OA is considered a whole-organ disease in which joint structures such as the ligaments, tendons, synovium, meniscus and subchondral bone are affected as well as the cartilage.[4] The causes of OA are not fully established; however, the strongest predictor for an OA diagnosis is age,[3] and disease development is associated with an interplay between different risk factors in combination with the common biomechanic denominator.[10 11] Physical inactivity due to the symptoms of OA is in itself a risk factor for physical and mental illness and premature death.[12] Individuals with OA have a higher risk for comorbidity-related mortality than the general population, especially in cardiovascular diseases.[13] With the increased prevalence of obesity and population ageing, OA is likely to be a major burden for health systems and a challenge for the future.[2 3]

Research and guidelines show that individually adapted physical exercises, information about OA, self-management and weight control help prevent disability and impairment in health,[14–16] and may have potential to delay replacement surgery[16 17] in individuals with OA. In Sweden, core treatment

For peer review only

BMJ Open: first published as 10.1136/bmjopen-2018-022812 on 3 October 2018. Downloaded from http://bmjopen.bmj.com/ on January 25, 2021 at i Jonkopings lan EIRA consortia. Protected by copyright.

For peer review only - http://bmjopen.bmj.com/site/about/guidelines.xhtml

for OA comprising education and supervised exercises in a supported self-management programme has been standardised since 2008. Evaluation and follow-ups are registered in the National Quality Register for Better Management of Patients with Osteoarthritis (BOA).[15]

Currently, the course of pain and physical functioning in individuals with hip or knee OA is difficult to predict and can vary over time.[4 18 19] Factors that may predict deterioration include high comorbidity, low level of education, presence of OA in several joints and a low physical activity level.[18 19] At this stage, early identification of those who will have a good prognosis of slow disease progression and those who will need more interventions or replacement surgery is not possible. Replacement surgery is an effective treatment method to decrease pain and improve function and health-related quality of life (HRQoL) in patients with OA. However, it is only suitable for a minority of patients,[20] those with severe symptoms in combination with radiographic-verified OA and where other non-surgical treatments have failed.[15 21] Treatment guidelines and systematic reviews describe three main indicators for replacement surgery: a) pain that does not respond to non-surgical treatment and b) impairment in HRQoL and function, in combination with c) radiographic changes. To optimise outcomes after surgery and reduce the risk of revision surgery, it is important that patients are carefully selected according to indicators and timing of surgery.[21 22] As the evidence base is weak and indicators for replacement surgery are mostly subjective, the need for surgery is often determined by the individual surgeon in consultation with the patient.[22] More knowledge is needed to facilitate early identification of factors that can predict deterioration of OA. This knowledge can be used to design treatment according to prognosis, improve criteria for surgery[18 19] and identify factors predicting better or worse postoperative outcomes.[22]

Merging data from the BOA Register with data from other health and socioeconomic data sources in Sweden will allow us to follow patients with OA through the progress of their disease. The overriding aim of this study is to increase knowledge about those who have received education and supervised exercises as a core treatment for hip and knee OA and are registered in the BOA Register, and identify factors that can predict long-term outcomes for this population.

METHODS AND ANALYSIS

Study design and research questions

This nationwide observational cohort study will use prospectively obtained individual-level data from five main sources: the BOA Register, the Swedish Hip Arthroplasty Register (SHAR), the Swedish Knee Arthroplasty Register (SKAR), Statistics Sweden and the National Board of Health and Welfare. Data will be merged using the personal identity number (PIN) that is unique to all citizens in Sweden. This will create a research database that will be used to answer three research questions:

1. What are the differences between the population included in the BOA Register and the general Swedish population regarding health and socioeconomic factors?
2. What factors that predict replacement surgery can be identified among patients with hip or knee OA at their first registration in the BOA Register?
3. What factors that predict outcomes after replacement surgery can be identified among patients with hip or knee OA at their first registration in the BOA Register?

Study population

The study cohort will comprise all patients with a first registration (baseline) in the BOA Register between 2008 and 2016 (approximately n=75 000). These patients sought treatment for knee and/or hip pain in primary healthcare in Sweden, and were referred for standardised core treatment (education and supervised exercises) after a confirmed clinical/radiographic OA diagnosis. Recommendations for OA diagnosis from the Swedish National Board of Health and Welfare are used to determine inclusion in the standardised core treatment and registration in the BOA Register; namely, an overall assessment of medical history, symptoms and clinical findings.[5] These guidelines are in line with internationally accepted diagnostic criteria,[6 7] and according to the guidelines, radiographic examination should only be used in uncertain cases, if the patient is not responding to treatment or when a surgical intervention is planned.

A reference cohort (approximately n=225 000) drawn from the general Swedish population who had never been included in the BOA Register will be identified from the Statistics Sweden Swedish Population Register, and matched (1:3) to each patient in the study cohort (as at baseline) by year of birth, sex and residence (geographical county areas in Sweden).

Data sources

The BOA Register

The BOA Register was initiated in 2008. Since 2010, it has been a national Swedish quality register that includes patients with hip/knee OA and evaluates the standardised core treatment (education and supervised exercises). Exclusion criteria for participation and registration in the BOA Register are: suspicion of or confirmed tumour, rheumatoid arthritis, sequel hip fracture, chronic pain or fibromyalgia, total joint replacement within the past 12 months, other surgery of the knee or hip joint within the past 3 months and patients not able to read or understand Swedish.[15] Data from patients and physiotherapists are reported to the BOA Register at baseline, and outcomes of the standardised core treatment are evaluated and reported through patient-reported outcome measures at 3 and 12 months and physiotherapist-reported outcome measures at 3 months[15] (see table 1 for description of single variables). This supported self-management programme is today an implemented activity that is available in all geographical county areas in Sweden,

For peer review only

For peer review only - http://bmjopen.bmj.com/site/about/guidelines.xhtml

BMJ Open: first published as 10.1136/bmjopen-2018-022812 on 3 October 2018. Downloaded from http://bmjopen.bmj.com/ on January 25, 2021 at i Jonkopings lan EiRA consortia. Protected by copyright.

Table 1 Description of the single variables collected from the Better Management of Patients with Osteoarthritis Register

BOA Register		First registration (baseline)	Evaluation 3 months	Evaluation 12 months
**Variable category**	**Variable**			
Date	Date of visits	x	x	x
Patient-reported measures	Age, sex, weight, height	x		
	Smoking	x		
	Most affected joint (hip, knee or hand)	x	x	x
	Other affected joints	x	x	x
	Fear avoidance	x	x	x
	Request for surgery	x	x	x
Physical activity level	Duration of physical training *	x	x	x
	Duration of physical activity †	x	x	x
Satisfaction	Satisfaction with treatment		x	x
Musculoskeletal comorbidity	Charnley class‡	x	x	x
Pain	Pain severity§ NRS	x	x	x
	Pain frequency	x	x	x
Health-related quality of life	EuroQol-5 dimensions	x	x	x
	EuroQol VAS	x	x	x
Self-efficacy	Arthritis self-efficacy scale	x	x	x
Physiotherapist-reported measures	Earlier radiography/MRI/surgery in the most affected or the contralateral joint	x		
	Earlier treatments (including physiotherapy/adapted training/ information on weight reduction/ pharmaceuticals)	x		
	Waiting list for surgery	x	x	
Follow-up	Radiography/MRI/surgery in the most affected or the contralateral joint since last evaluation		x	
	Compliance with intervention		x	

*Answering to the question: "During a regular week, how much time do you spend exercising on a level that makes you short winded, for example, running, fitness class or ball games?" graded on categorical scale from '0' to '>120 min'.
†Answering to the question: "During a regular week, how much time are you physically active in ways that are not exercise, for example, walks, bicycling or gardening?" graded on categorical scale from '0' to '>300 min'.[34] These questions have been developed by the National Board of Health and Welfare in Sweden for population-based studies on physical activity and have been validated against an accelerometer.[34]
‡Charnley class: classifications of musculoskeletal impairment. Class A corresponds with unilateral hip or knee OA; class B bilateral hip or knee OA and class C indicates multiple joint OA or some other condition that inhibits the patient's ability to walk.[35]
§Answering to: "Select the box that corresponds to your average pain from your most affected joint the last week".
NRS, numeric rating scale; OA, osteoarthritis; VAS, visual analogue scale; MRI, magnetic resonance imaging.

with approximate 450 units conducting the therapy and reports to the BOA Register. At the end of 2016, the BOA Register comprised approximately 75 000 patients, with a mean age of 66±9.6 years.[23] The level of coverage (how many of the units that are running the intervention of standardised core treatment, that also register in the BOA Register) calculated by the end of 2014 was 80% and the level of completeness 69% (how many of the patients that have participated in the intervention of standardised core treatment that have been reported to the register). The response rate for each reported question at baseline and 3 mounts was 97% and at 1 year 84%.[24]

The SHAR and SKAR

The SHAR and SKAR collect patient-related and procedure-related data on all hip and knee replacement surgeries in Sweden (tables 2 and 3). In SHAR, patient-reported outcomes such as joint pain, HRQoL and satisfaction with treatment are registered preoperatively and 1, 6 and 10 years postoperatively. Since 2008, SKAR has

For peer review only - http://bmjopen.bmj.com/site/about/guidelines.xhtml
BMJ Open: first published as 10.1136/bmjopen-2018-022812 on 3 October 2018. Downloaded from http://bmjopen.bmj.com/ on January 25, 2021 at i Jonkopings lan EiRA consortia. Protected by copyright.

Table 2 Description of single variables collected from the Swedish Hip Arthroplasty Register

Swedish Hip Arthroplasty Register		Before surgery	Follow-up 1 year
**Variable category**	**Variable**		
Diagnosis (at hip)	ICD-10	x	
	Laterality	x	
Date	Date of surgery	x	
Type of surgery	Primary, revision, reoperation	x	
	Total, partial, resurfacing hip replacement	x	
Patient-reported measures	Smoking	x	
Musculoskeletal comorbidity	Charnley class*	x	x
Pain	Hip pain VAS	x	x
Health-related quality of life	EuroQol-5 dimensions	x	x
	EuroQol VAS	x	x
Treatment before hip replacement surgery	Physiotherapy	x	
	Standardised core treatment of education and supervised exercises	x	
Satisfaction	Satisfaction with treatment		x

*Charnley class: classifications of musculoskeletal impairment. Class A corresponds to unilateral hip disease; class B bilateral hip disease and class C indicates multiple joint disease or some other condition that inhibits the patient's ability to walk.
ICD-10, International Classification of Diseases, tenth revision; VAS, visual analogue scale.

Table 3 Description of single variables collected from Swedish Knee Arthroplasty Register

Swedish Knee Arthroplasty Register		Before surgery	Follow-up 1 year
**Variable category**	**Variable**		
Diagnosis (at knee)	ICD-10	x	
	Laterality	x	
Date	Date of surgery	x	
Type of surgery	Primary, revision, reoperation	x	
	Total, partial, resurfacing knee replacement	x	
Patient-reported measures			
Musculoskeletal comorbidity	Charnley class*	x	x
Pain	Knee pain VAS	x	x
Health-related quality of life	EuroQol-5 dimensions	x	x
	EuroQol VAS	x	x
Satisfaction	Satisfaction with treatment		x
Patient-reported knee function	Knee injury and Osteoarthritis Outcome Score	x	x

*Charnley class: classifications of musculoskeletal impairment. Class A corresponds to unilateral knee disease; class B to bilateral knee disease and class C to multiple joint disease or some other condition that inhibits the patient's ability to walk.
ICD-10, International Classification of Diseases, tenth revision; VAS, visual analogue scale.

successively registered preoperative and 1-year postoperative patient-reported outcomes (the same variables as in SHAR along with the knee-specific instrument Knee Injury and Osteoarthritis Outcome Score). In 2016, the SKAR included patient-reported outcomes for approximately 33% of all primary knee replacement surgeries. These registers are two of the oldest quality registers in Sweden; SHAR started in 1979 and SKAR in 1975. Both registers have a high completeness (97%–98%) (number of reported hip/knee replacements divided by the number of hip/knee replacements performed).[25 26] The response rate to the patient-reported outcomes is in the SHAR 85%, and in the SKAR 84% (calculated on the hospitals who register patient-reported measures). Each year, approximately 14 000 OA-related primary hip replacement surgeries and 12 000 primary knee replacement surgeries are performed in Sweden. The mean age of patients in both registers is approximately 69 years.[25 26]

Statistics Sweden

Statistics Sweden is an administrative governmental agency. The agency's main task is to supply users and consumers with statistics for decision-making and microdata for research purposes (after ethical approval). Statistics Sweden administers several different databases. In this study, socioeconomic microdata will be drawn from the Swedish Population Register and the Longitudinal Integration Database for Health Insurance and Labour Market Studies (LISA). The Swedish Population Register provides data for vital events such as births, deaths, residence and marital status, and LISA provides socioeconomic data (table 4).

The National Board of Health and Welfare

The National Board of Health and Welfare is a Swedish government agency that sits under the Ministry of Health and Social Affairs. Their main task is to contribute to

For peer review only - http://bmjopen.bmj.com/site/about/guidelines.xhtml

BMJ Open: first published as 10.1136/bmjopen-2018-022812 on 3 October 2018. Downloaded from http://bmjopen.bmj.com/ on January 25, 2021 at i Jonkopings lan EiRA consortia. Protected by copyright.

Table 4 Description of single variables collected from Statistics Sweden

Statistics Sweden		From 3 years before baseline	Baseline	End of 2016
**Variable category**	**Variable**			
The Swedish Population Register				
Demography	Year of birth, place of birth		x	
	Sex, residence		x	
	Relocation within Sweden, emigration		x	
	Marital status		x	
	Date of death			x
The Longitudinal Integration Database for Health Insurance and Labour Market Studies				
Income	Individual disposable income	x		
	Family disposable income	x		
Family circumstances	Family type		x	
	Children at home		x	
	Number of people in the household		x	
Education	Educational attainment		x	
	Year of graduation from highest education		x	
Sickness benefits	Days with sickness compensation	x		
	Total income due to sickness	x		
	Days with occupational injury compensation	x		
	Total income due to occupational injury	x		
	Days with rehabilitation compensation	x		
	Total income due to rehabilitation	x		
Unemployment benefits	Unemployment classification	x		
	Total income from unemployment compensation	x		
	Days unemployed	x		
	Days partly unemployed	x		
Welfare benefits	Family welfare benefits	x		
	Housing benefits	x		
Occupation	Occupational classification		x	

equal access to good health and social care by managing healthcare regulations and providing guidelines. All healthcare providers are mandated to continuously report to the authority's health data registers. These data are used to produce and develop statistics for follow-ups and evaluation of the healthcare system. In addition, after ethical approval, researchers can access information from the Board's different health data registers. The Swedish National Patient Register started in 1964, is nationwide since 1987 and covers all inpatient care and since 2001 all specialised outpatient visits (including day surgery) with diagnosis codes (International Classification of Diseases, tenth revision) and admission/discharge dates from both private and public caregivers. However, primary healthcare is not yet covered, and the register only contains information about healthcare provided by physicians.

For peer review only - http://bmjopen.bmj.com/site/about/guidelines.xhtml

BMJ Open: first published as 10.1136/bmjopen-2018-022812 on 3 October 2018. Downloaded from http://bmjopen.bmj.com/ on January 25, 2021 at i Jonkopings lan EiRA consortia. Protected by copyright.

Table 5 Description of single variables collected from the National Board of Health and Welfare

National Board of Health and Welfare		From 3 years before baseline	End of 2016
**Variable category**	**Variable**		
National Patient Register			
Diagnosis	ICD-10	x	
	Diagnosis-related grouping	x	
	Main diagnosis	x	
Intervention	Classification of intervention, including replacement surgery in hip/knee (x)	x	(x)
Date	Admission	x	
	Discharge	x	
	Elective or acute care	x	
	Hospital name	x	
Type of care	Outpatient/inpatient	x	
Swedish Prescribed Drug Register			
Date	Prescription date	x	
	Withdrawal date	x	
Drug	Name	x	
	Dosage	x	
	Anatomic Therapeutic Chemical Classification system	x	
Cost	Patient cost	x	
	Subsidised cost	x	

ICD-10, International Classification of Diseases, tenth revision.

The Swedish Prescribed Drug Register started in 2005 and contains information on expenditure for prescribed drugs. Over-the-counter drugs are not included.[27] In this study, we will use data for comorbidity and healthcare consumption from the National Patient Register and data for expenditure on prescribed drugs from the Swedish Prescribed Drug Register, to the end of 2016 (table 5).

PERSONAL IDENTITY NUMBER

In Sweden, all citizens are registered with a unique PIN that provides information on date of birth and sex. Swedish law requires all documentation regarding healthcare contacts to be registered using the patient's PIN.[28] The same system is used for registration of data for all types of statistics, such as national population-based registers and healthcare quality registers.[29 30] The PIN also allows linkage of data at an individual level between the different registers in Sweden with the possibility of creating merged research databases for epidemiological research on large populations, after the relevant ethical approval has been obtained.[27 28]

Collection of data

The merged database will be created using specific steps (figure 1):

1. Using the unique PIN, a search will be made to determine if any patients in the BOA Register were registered in the SHAR/SKAR before the end of 2016. If so, these data will be merged with BOA data.
2. The data set will then be submitted to Statistics Sweden, where requested variables will be added using the PIN as the unique identifier.
3. Statistics Sweden will replace each PIN with a serial number to anonymise data before the merged database will be returned to the researchers.
4. At the same time, Statistics Sweden will forward the list of PINs and serial numbers to the National Board of Health and Welfare to obtain requested variables.
5. The National Board of Health and Welfare will send data for these variables to the researchers using only the serial numbers, and the serial numbers will be used to merge all data to create the research database.
6. According to the matching criteria, Statistics Sweden will generate the reference cohort, and perform the procedure as described above to add requested variables, anonymise individuals by replacing the PIN with a serial number, send the data to the researchers and forward the list of PINs/serial numbers to the National Board of Health and Welfare.

The key for linking the serial number to the PIN will be saved at Statistics Sweden (without access for the

For peer review only - http://bmjopen.bmj.com/site/about/guidelines.xhtml

BMJ Open: first published as 10.1136/bmjopen-2018-022812 on 3 October 2018. Downloaded from http://bmjopen.bmj.com/ on January 25, 2021 at i Jonkopings lan EiRA consortia. Protected by copyright.

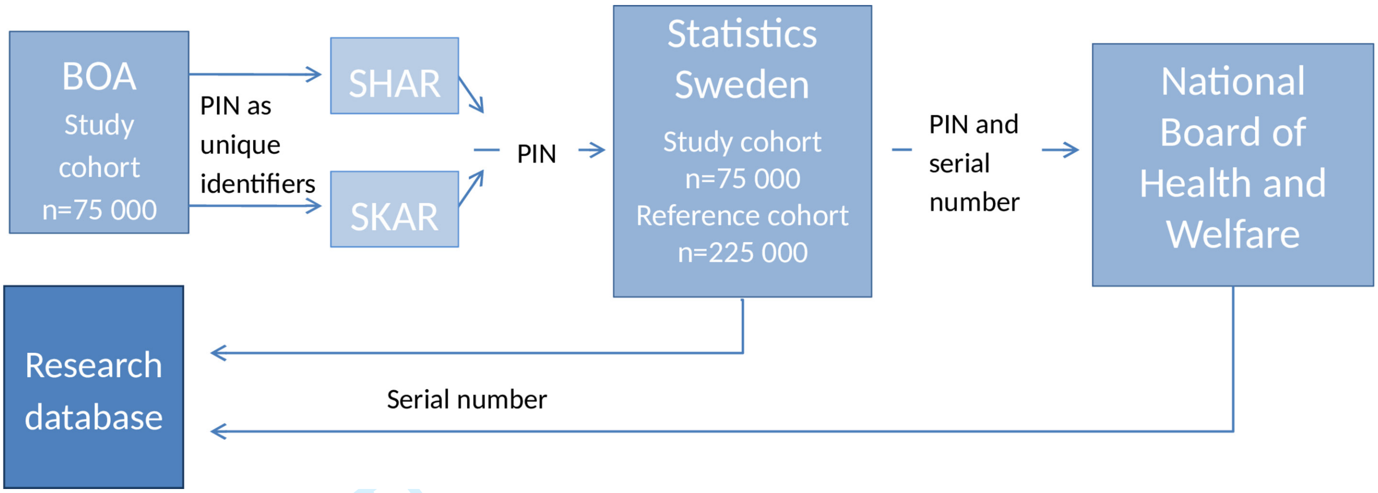

Figure 1 Description of data collection from the Better Management of Patients with Osteoarthritis Register (BOA), the Swedish Hip Arthroplasty Register (SHAR), the Swedish Knee Arthroplasty Register (SKAR), Statistics Sweden and the National Board of Health and Welfare to create a merged research database on patients with osteoarthritis. PIN, personal identity number.

researchers) for 3 years to allow the possibility of adding more year cohorts (eg, 2017, 2018) or new variables to the research database if new research questions arise (with new ethical approval).

Analysis plan

Since the population in the BOA Register has not been described before or used to study progress of OA over time or treatment effects in OA, the aim with this first part of the study is to contribute to the clarification of the registered patients, by validating the individuals in the BOA Register against the general Swedish population. This will evaluate if the two groups are equal or not based on socioeconomic status and health. We will however not be able to compare the cohorts based on lifestyle habits or other OA risk factors since these data not are available for the reference cohort. Depending on the results from the validation of the BOA population, we further on will be able to discuss the results from the coming research questions in relation to other individuals. It is likely that the study also will generate hypothesis that later can be tested in new studies with other designs.

First research question

The first research question has a descriptive design with both a cross-sectional and longitudinal approach, depending on the different variables that will be studied. Socioeconomic factors (marital status, education level, occupation, country of birth) will be described and compared at baseline. Income, comorbidity (from calculated comorbidity index), healthcare consumption (defined as the total number of healthcare contacts per capita), sick leave and expenditure on prescribed drugs (the amount of analgesic and joint-related) will be described and compared for 3 years before baseline. Finally, the two groups will be described and compared based on eventual replacement surgery or death, up to the end of 2016.

Second research question

The main outcome to answer the second research question is replacement surgery or not. Only the patients from the study cohort will be used in this evaluation. The study cohort will be divided into two groups depending on if they had hip/knee replacement surgery before the end of 2016 (replacement group and non-replacement group). Socioeconomic factors, comorbidity and patient-reported measures at baseline will be compared between the groups and evaluated as eventual risk or health factors for the outcome replacement surgery.

Third research question

To answer the third research question, only the patients from the study cohort who had a hip or knee replacement up to the end of 2015 will be evaluated. The outcome that will be studied is patient-reported outcome measures (joint pain, HRQoL and satisfaction with treatment) from SHAR and SKAR, respectively. The patients will be grouped depending on better or worse patient-reported outcomes 1 year after surgery. The groups (better or worse postoperative outcome) will then be compared to identify differences in socioeconomic factors, comorbidity or patient-reported measures at baseline in the BOA Register, as risk or health factors for outcome after replacement surgery.

The patient-reported outcome measures at 3 and 12 months and the physiotherapist-reported outcome measures at 3 months registered in the BOA Register will also be analysed as eventual predictors for future replacement surgery and for outcome after replacement surgery.

Calculations from the BOA Register, the SHAR Register and the SKAR Register has shown that, in the study population, approximately 10 400 individuals have had a hip replacement surgery and 4900 individuals have had a knee replacement surgery by the end of 2016.

For peer review only - http://bmjopen.bmj.com/site/about/guidelines.xhtml
BMJ Open: first published as 10.1136/bmjopen-2018-022812 on 3 October 2018. Downloaded from http://bmjopen.bmj.com/ on January 25, 2021 at i Jonkopings lan EiRA consortia. Protected by copyright.

Statistical analysis

Statistical analysis of the data will be performed descriptively and comparatively using parametric or non-parametric statistics depending on the nature of the data, with adjustment for potential confounders by multivariable regression analysis. Separate analyses will be performed for each research question. We will be able to account and adjust for OA in multiple joints and/or multiple joint replacements. Multivariable regression models will be used to analyse the factors (eg, socioeconomic factors, comorbidity and patient-reported measures) that can predict replacement surgery (second research question) and better or worse patient-reported outcomes 1 year after replacement surgery (third research question).

Patients can take different paths after entry into the BOA Register. Ideally, patients stay in their entry stage; however, a patient can proceed to the next stage of having their knee(s) or hip(s) replaced, and further to die during the follow-up period (up to the end of 2016). To identify risk factors that may be associated with transition from one stage to another, we will use competing risks and cumulative incidences to estimate the proportion of patients that moved to each stage. It is likely that we will need to extend this analysis and apply a modified 'illness-death' model. Hip and knee replacements are non-terminal events, and patients can stay in these stages throughout the entire follow-up period or move to the next stage. Death is a terminal stage. Analysis of this model will proceed with semi-competing risks or alternatively a multistate regression analysis.[31 32] The aim of this analysis is to identify risk factors that may be associated with transition from one stage to another.

Survival of the hip/knee joint for patients in the study cohort will be summarised using Kaplan-Meier curves to estimate the probability of replacement surgery within a certain time. Additionally, we will compare the survival of the joint for those patients with the reference cohort using relative survival.[33]

Patient and public involvement

Patients were not involved in the development of the research question or the design of this study. Patients were however involved in the creation of the supported self-management programme and contributed with the key content of the programme and the mode of delivery from the patient perspective. If possible, a trained OA communicator is also participating in the education-part during the standardised core treatments to to teach about the experience of living with OA, as well as his or her personal experience of non-surgical interventions.[15]

ETHICS AND DISSEMINATION

Storage and management of data

The anonymised research database with data for the study and reference cohorts that only uses serial numbers will be administered by the Center of Registers Västra Götaland, Gothenburg, Sweden. Data will be stored on encrypted servers at Gothenburg University, and will only be accessible by researchers involved in the study. A statistician at the Center of Registers Västra Götaland will structure the data. The structured data will then be uploaded to a virtual computer (Secure On-line Data Access) by the researchers involved. The researchers can obtain remote access to the research database after double identification, but exporting or downloading of individual data is not possible. The process of linkage, storage and management of data, the role of informed consent in register-based research and safeguarding the integrity of study participants follows the legal and ethical frameworks as described by Swedish law and ethical boards. This has previously been described by Ludvigsson *et al*.[27]

Dissemination of results

The results from this study will be published in peer-reviewed journals and at suitable national and international meetings. The results will also be disseminated through the relevant quality registers.

DISCUSSION

This study will provide unique data on patients with hip and/or knee OA throughout the progression of their disease. By merging data from the BOA Register with data from other Swedish health and socioeconomic data sources, we will be able to identify factors that influence the development of OA and factors that affect long-term outcomes, such as the need for replacement surgery and perceived function after surgery in patients with OA. To our knowledge, this study will represent the largest collection of data from a national OA population, and provide a unique opportunity to study a range of factors that may influence the progression of OA. In Sweden, the PIN and the various disorder-focused health data registers allow linking data from different sources, thereby allowing large populations to be followed over several years. All aspects that are affecting individuals with OA are however difficult to collect in the same research database. For example, we cannot capture OA-related items such as range of motion and eventual joint deformities, and we have no data from primary healthcare in Sweden, since this is not collected on a national basis. These are examples of weaknesses in the database and these limitations will be highlighted and discussed in relation to our results in the coming papers.

To optimise core treatment in OA and design treatment according to prognosis, it is important to identify patients that have a good prognosis and those who will need more interventions or future replacement surgery early in the course of disease. Knowledge about the progression of OA in different individuals and

BMJ Open: first published as 10.1136/bmjopen-2018-022812 on 3 October 2018. Downloaded from http://bmjopen.bmj.com/ on January 25, 2021 at i Jonkopings lan EiRA consortia. Protected by copyright.

For peer review only

their differing needs for healthcare interventions is important to manage this growing disease in future.

Author affiliations
[1]Department of Physiotherapy, Rehabilitation Centre, Ryhov County Hospital Jönköping, Jönköping, Sweden
[2]Division of Physiotherapy, Department of Medical and Health Sciences, Linköping University, Linköping, Sweden
[3]Department of Orthopaedics, Sahlgrenska Academy, Institute of Clinical Sciences, University of Gothenburg, Gothenburg, Sweden
[4]Department of Medical and Health Sciences, Linköping University, and Futurum, Region Jönköping County, Jönköping, Sweden
[5]Department of Clinical Sciences Lund, Orthopedics, Faculty of Medicine, Lund University and Clinical Epidemiology Unit, Skåne University Hospital, Lund, Sweden
[6]Division of Physiotherapy, Department of Neurobiology, Care Sciences and Society, Karolinska Institutet, Stockholm, Sweden

Contributors All authors have substantially contributed to the conception and design of the study. KG wrote the first draft of the paper, which was critically revised by the other authors. All authors approved the final version of the manuscript.

Funding The study is financially supported by AFA Insurance, Sweden (160176). The preparation of the study has been financially supported by Futurum—Academy for Health and Care, Region Jönköping County, Sweden (559951,557821).

Competing interests None declared.

Patient consent Not required.

Ethics approval Ethical approval for this study has been granted by the Regional Ethical Review Board in Gothenburg, Sweden (dnr 1059–16).

Provenance and peer review Not commissioned; externally peer reviewed.

Open access This is an open access article distributed in accordance with the Creative Commons Attribution Non Commercial (CC BY-NC 4.0) license, which permits others to distribute, remix, adapt, build upon this work non-commercially, and license their derivative works on different terms, provided the original work is properly cited, appropriate credit is given, any changes made indicated, and the use is non-commercial. See: http://creativecommons.org/licenses/by-nc/4.0/.

REFERENCES

1. Vos T, Abajobir A, Abate K, *et al*. Global, regional, and national incidence, prevalence, and years lived with disability for 328 diseases and injuries for 195 countries, 1990-2016: a systematic analysis for the Global burden of disease study 2016. *Lancet* 2017;390:1211–59.
2. Turkiewicz A, Petersson IF, Björk J, *et al*. Current and future impact of osteoarthritis on health care: a population-based study with projections to year 2032. *Osteoarthritis Cartilage* 2014;22:1826–32.
3. Cross M, Smith E, Hoy D, *et al*. The global burden of hip and knee osteoarthritis: estimates from the global burden of disease 2010 study. *Ann Rheum Dis* 2014;73:1323–30.
4. Roos EM, Arden NK. Strategies for the prevention of knee osteoarthritis. *Nat Rev Rheumatol* 2016;12:92–101.
5. Socialstyrelsen. Nationella riktlinjer för rörelseorganens sjukdomar 2012: Osteoporos, artros, inflammatoriskryggsjukdom och ankyloserande spondylit, psoriasisartrit och reumatoid artrit: Stöd för styrning och ledning [Swedish]. *Socialstyrelsen* 2012.
6. Altman R, Alarcón G, Appelrouth D, *et al*. The American College of Rheumatology criteria for the classification and reporting of osteoarthritis of the hip. *Arthritis Rheum* 1991;34:505–14.
7. Altman R, Asch E, Bloch D, *et al*. Development of criteria for the classification and reporting of osteoarthritis. Classification of osteoarthritis of the knee. Diagnostic and therapeutic criteria committee of the American Rheumatism Association. *Arthritis Rheum* 1986;29:1039–49.
8. Jordan KP, Tan V, Edwards JJ, *et al*. Influences on the decision to use an osteoarthritis diagnosis in primary care: a cohort study with linked survey and electronic health record data. *Osteoarthritis Cartilage* 2016;24:786–93.
9. Bedson J, Croft PR. The discordance between clinical and radiographic knee osteoarthritis: a systematic search and summary of the literature. *BMC Musculoskelet Disord* 2008;9:116.
10. Felson DT. Osteoarthritis as a disease of mechanics. *Osteoarthritis Cartilage* 2013;21:10–15.
11. Neogi T, Zhang Y. Epidemiology of osteoarthritis. *Rheum Dis Clin North Am* 2013;39:1–19.
12. WHO Health Statistics. Physical activity. Secondary physical activity. http://www.who.int/mediacentre/factsheets/fs385/en/ (accessed Feb 2017).
13. Nüesch E, Dieppe P, Reichenbach S, *et al*. All cause and disease specific mortality in patients with knee or hip osteoarthritis: population based cohort study. *BMJ* 2011;342:d1165.
14. Zhang W, Nuki G, Moskowitz RW, *et al*. OARSI recommendations for the management of hip and knee osteoarthritis: part III: Changes in evidence following systematic cumulative update of research published through January 2009. *Osteoarthritis Cartilage* 2010;18:476–99.
15. Thorstensson CA, Garellick G, Rystedt H, *et al*. Better management of patients with osteoarthritis: Development and nationwide implementation of an evidence-based supported osteoarthritis self-management programme. *Musculoskeletal Care* 2015;13:67–75.
16. Altman RD. Early management of osteoarthritis. *Am J Manag Care* 2010;16 Suppl Management:S41–7.
17. Svege I, Nordsletten L, Fernandes L, *et al*. Exercise therapy may postpone total hip replacement surgery in patients with hip osteoarthritis: a long-term follow-up of a randomised trial. *Ann Rheum Dis* 2015;74:164–9.
18. de Rooij M, van der Leeden M, Heymans MW, *et al*. Course and predictors of pain and physical functioning in patients with hip osteoarthritis: Systematic review and meta-analysis. *J Rehabil Med* 2016;48:245–52.
19. de Rooij M, van der Leeden M, Heymans MW, *et al*. Prognosis of pain and physical functioning in patients with knee osteoarthritis: A systematic review and meta-analysis. *Arthritis Care Res* 2016;68:481–92.
20. Peat G, McCarney R, Croft P. Knee pain and osteoarthritis in older adults: a review of community burden and current use of primary health care. *Ann Rheum Dis* 2001;60:91–7.
21. Rolfson O, Rothwell A, Sedrakyan A, *et al*. Use of patient-reported outcomes in the context of different levels of data. *J Bone Joint Surg Am* 2011;93 Suppl 3(Suppl 3):66–71.
22. Gademan MG, Hofstede SN, Vliet Vlieland TP, *et al*. Indication criteria for total hip or knee arthroplasty in osteoarthritis: a state-of-the-science overview. *BMC Musculoskelet Disord* 2016;17:463.
23. Wetterling K, Muller J, Bergsten C. *BOA-registret Årsrapport 2016*. Göteborg, 2017.
24. Thorstensson CA, Dahlberg L, Garellick G. *The BOA-register annual report 2014*. Gothenburg, 2015.
25. Kärrholm J, Lindahl H, Malchau H, *et al*. *The swedish hip arthroplasty register annual report 2016*. Gothenburg, 2017.
26. Robertsson O, W-Dahl A, Lidgren L, *et al*. *Å rsrapport 2017 Svenska Knäprotesregistret*. Lund, 2017.
27. Ludvigsson JF, Håberg SE, Knudsen GP, *et al*. Ethical aspects of registry-based research in the Nordic countries. *Clin Epidemiol* 2015;7:491–508.
28. Ludvigsson JF, Otterblad-Olausson P, Pettersson BU, *et al*. The swedish personal identity number: Possibilities and pitfalls in healthcare and medical research. *Eur J Epidemiol* 2009;24:659–67.
29. Ludvigsson JF, Almqvist C, Bonamy AK, *et al*. Registers of the Swedish total population and their use in medical research. *Eur J Epidemiol* 2016;31:125–36.
30. Ludvigsson JF, Andersson E, Ekbom A, *et al*. External review and validation of the Swedish national inpatient register. *BMC Public Health* 2011;11:450.
31. Lee KH, Haneuse S, Schrag D, *et al*. Bayesian semi-parametric analysis of semi-competing risks data: Investigating hospital readmission after a pancreatic cancer diagnosis. *J R Stat Soc Ser C Appl Stat* 2015;64:253–73.
32. Jackson CH. Multi-state models for panel data: the MSM package for *R*. *J Stat Softw* 2011;38:1–28.
33. Pohar M, Stare J. Making relative survival analysis relatively easy. *Comput Biol Med* 2007;37:1741–9.
34. Olsson SJ, Ekblom Ö, Andersson E, *et al*. Categorical answer modes provide superior validity to open answers when asking for level of physical activity: a cross-sectional study. *Scand J Public Health* 2016;44:70–6.
35. Charnley J. The long-term results of low-friction arthroplasty of the hip performed as a primary intervention. *J Bone Joint Surg Br* 1972;54:61–76.

For peer review only - http://bmjopen.bmj.com/site/about/guidelines.xhtml

BMJ Open: first published as 10.1136/bmjopen-2018-022812 on 3 October 2018. Downloaded from http://bmjopen.bmj.com/ on January 25, 2021 at i Jonkopings lan EiRA consortia. Protected by copyright.

Reporting checklist for cross sectional study.

Based on the STROBE cross sectional guidelines.

Instructions to authors

Complete this checklist by entering the page numbers from your manuscript where readers will find each of the items listed below.

Your article may not currently address all the items on the checklist. Please modify your text to include the missing information. If you are certain that an item does not apply, please write "n/a" and provide a short explanation.

Upload your completed checklist as an extra file when you submit to a journal.

In your methods section, say that you used the STROBE cross sectionalreporting guidelines, and cite them as:

von Elm E, Altman DG, Egger M, Pocock SJ, Gotzsche PC, Vandenbroucke JP. The Strengthening the Reporting of Observational Studies in Epidemiology (STROBE) Statement: guidelines for reporting observational studies.

		Reporting Item	Page Number
**Title and abstract**			
Title	#1a	Indicate the study's design with a commonly used term in the title or the abstract	page 1

For peer review only

Abstract	#1b	Provide in the abstract an informative and balanced summary of what was done and what was found	page 3
**Introduction**			
Background / rationale	#2	Explain the scientific background and rationale for the investigation being reported	page 5
Objectives	#3	State specific objectives, including any prespecified hypotheses	page 6
**Methods**			
Study design	#4	Present key elements of study design early in the paper	page 6
Setting	#5	Describe the setting, locations, and relevant dates, including periods of recruitment, exposure, follow-up, and data collection	page 6-7
Eligibility criteria	#6a	Give the eligibility criteria, and the sources and methods of selection of participants.	page 6-7
	#7	Clearly define all outcomes, exposures, predictors, potential confounders, and effect modifiers. Give diagnostic criteria, if applicable	page 9-10, and 14
Data sources / measurement	#8	For each variable of interest give sources of data and details of methods of assessment	page 9-10, and 14, and Supplemenary file 1-2

For peer review only

		(measurement). Describe comparability of assessment methods if there is more than one group. Give information separately for for exposed and unexposed groups if applicable.	
Bias	#9	Describe any efforts to address potential sources of bias	page 7
Study size	#10	Explain how the study size was arrived at	page 6-7
Quantitative variables	#11	Explain how quantitative variables were handled in the analyses. If applicable, describe which groupings were chosen, and why	page 14
Statistical methods	#12a	Describe all statistical methods, including those used to control for confounding	page 14
Statistical methods	#12b	Describe any methods used to examine subgroups and interactions	page 14
Statistical methods	#12c	Explain how missing data were addressed	n/a, there was only missing data for the variables that were adjusted for (to a small extend), as described in table 1 page 7

For peer review only

Statistical methods	#12d	If applicable, describe analytical methods taking account of sampling strategy	page 14
Statistical methods	#12e	Describe any sensitivity analyses	page 14
**Results**			
Participants	#13a	Report numbers of individuals at each stage of study—eg numbers potentially eligible, examined for eligibility, confirmed eligible, included in the study, completing follow-up, and analysed. Give information separately for for exposed and unexposed groups if applicable.	page 9 and 14
Participants	#13b	Give reasons for non-participation at each stage	page 9
Participants	#13c	Consider use of a flow diagram	page 9
Descriptive data	#14a	Give characteristics of study participants (eg demographic, clinical, social) and information on exposures and potential confounders. Give information separately for exposed and unexposed groups if applicable.	page 8 and 16
Descriptive data	#14b	Indicate number of participants with missing data for each variable of interest	page 8

For peer review only

Outcome data	#15	Report numbers of outcome events or summary measures. Give information separately for exposed and unexposed groups if applicable.	page 8, 11-12 and 16-19
Main results	#16a	Give unadjusted estimates and, if applicable, confounder-adjusted estimates and their precision (eg, 95% confidence interval). Make clear which confounders were adjusted for and why they were included	page 17-19
Main results	#16b	Report category boundaries when continuous variables were categorized	page 7, table 1 - age
Main results	#16c	If relevant, consider translating estimates of relative risk into absolute risk for a meaningful time period	n/a, not relevant
Other analyses	#17	Report other analyses done—e.g., analyses of subgroups and interactions, and sensitivity analyses	page 19
**Discussion**			
Key results	#18	Summarise key results with reference to study objectives	page 19-20
Limitations	#19	Discuss limitations of the study, taking into account sources of potential bias or	page 22-23

For peer review only

		imprecision. Discuss both direction and magnitude of any potential bias.	
Interpretation	#20	Give a cautious overall interpretation considering objectives, limitations, multiplicity of analyses, results from similar studies, and other relevant evidence.	page 21-22
Generalisability	#21	Discuss the generalisability (external validity) of the study results	page 23
**Other Information**			
Funding	#22	Give the source of funding and the role of the funders for the present study and, if applicable, for the original study on which the present article is based	page 25

Notes:

- 7: page 9-10, and 14
- 8: page 9-10, and 14, and Supplemenary file 1-2
- 12c: n/a, there was only missing data for the variables that were adjusted for (to a small extend), as described in table 1 page 7
- 13a: page 9 and 14
- 14a: page 8 and 16
- 15: page 8, 11-12 and 16-19

For peer review only

- 16b: page 7, table 1 - age

- 16c: n/a, not relevant The STROBE checklist is distributed under the terms of the Creative Commons Attribution License CC-BY. This checklist was completed on 25. January 2021 using https://www.goodreports.org/, a tool made by the EQUATOR Network in collaboration with Penelope.ai

For peer review only

BMJ Open

Health status of individuals referred to first-line intervention for hip and knee osteoarthritis compared with the general population: an observational register-based study

Journal:	*BMJ Open*
Manuscript ID	bmjopen-2021-049476.R1
Article Type:	Original research
Date Submitted by the Author:	25-May-2021
Complete List of Authors:	Gustafsson , Kristin; Linköping University, Unit of Physiotherapy, Department of Health, Medicine and Caring Sciences,; Ryhov County Hospital, Department of Physiotherapy, Rehabilitation Centre,<br>Kvist, Joanna; Linköping University, Unit of Physiotherapy, Department of Health, Medicine and Caring Sciences,<br>Eriksson , Marit ; Linköping University and Futurum - Academy for Health and Care, Department of Medical and Health Sciences<br>Dell'Isola, Andrea; Lund University, Department of Clinical Sciences Lund<br>Zhou, Caddie; Centre of Registries, Västra Götaland,<br>Dahlberg, L; Lund University, Department of Clinical Sciences Lund<br>Rolfson, O; University of Gothenburg, Department of Orthopaedics
**Primary Subject Heading**:	Epidemiology
Secondary Subject Heading:	Health services research
Keywords:	Hip < ORTHOPAEDIC & TRAUMA SURGERY, Knee < ORTHOPAEDIC & TRAUMA SURGERY, EPIDEMIOLOGY, Musculoskeletal disorders < ORTHOPAEDIC & TRAUMA SURGERY

SCHOLARONE™
Manuscripts

I, the Submitting Author has the right to grant and does grant on behalf of all authors of the Work (as defined in the below author licence), an exclusive licence and/or a non-exclusive licence for contributions from authors who are: i) UK Crown employees; ii) where BMJ has agreed a CC-BY licence shall apply, and/or iii) in accordance with the terms applicable for US Federal Government officers or employees acting as part of their official duties; on a worldwide, perpetual, irrevocable, royalty-free basis to BMJ Publishing Group Ltd ("BMJ") its licensees and where the relevant Journal is co-owned by BMJ to the co-owners of the Journal, to publish the Work in this journal and any other BMJ products and to exploit all rights, as set out in our licence.

The Submitting Author accepts and understands that any supply made under these terms is made by BMJ to the Submitting Author unless you are acting as an employee on behalf of your employer or a postgraduate student of an affiliated institution which is paying any applicable article publishing charge ("APC") for Open Access articles. Where the Submitting Author wishes to make the Work available on an Open Access basis (and intends to pay the relevant APC), the terms of reuse of such Open Access shall be governed by a Creative Commons licence – details of these licences and which Creative Commons licence will apply to this Work are set out in our licence referred to above.

Other than as permitted in any relevant BMJ Author's Self Archiving Policies, I confirm this Work has not been accepted for publication elsewhere, is not being considered for publication elsewhere and does not duplicate material already published. I confirm all authors consent to publication of this Work and authorise the granting of this licence.

For peer review only

Health status of individuals referred to first-line intervention for hip and knee osteoarthritis compared with the general population: an observational register-based study

Kristin Gustafsson,[1,2] Joanna Kvist,[1] Marit Eriksson,[3] Andrea Dell'Isola,[4] Caddie Zhou,[5] Leif E. Dahlberg,[4] Ola Rolfson[6]

[1]Unit of Physiotherapy, Department of Health, Medicine and Caring Sciences, Linköping University, Linköping, Sweden

[2]Department of Physiotherapy, Rehabilitation Centre, Ryhov County Hospital, Jönköping, Sweden

[3]Department of Medical and Health Sciences, Linköping University and Futurum - Academy for Health and Care, Region Jönköping County, Jönköping, Sweden

[4]Department of Clinical Sciences Lund, Orthopaedics, Faculty of Medicine, Lund University, Lund, Sweden

[5]Centre of Registries, Västra Götaland, Gothenburg, Sweden

[6]Department of Orthopaedics, Sahlgrenska Academy, University of Gothenburg, Gothenburg, Sweden

E-mail:

Kristin Gustafsson – kristin.gustafsson@liu.se, kristin.gustafsson@rjl.se

Joanna Kvist – joanna.kvist@liu.se

Marit Eriksson – marit.eriksson@rjl.se

Andrea Dell'Isola - andrea.dellisola@med.lu.se

For peer review only - http://bmjopen.bmj.com/site/about/guidelines.xhtml

Caddie Zhou - caddie.zhou@vgregion.se

Leif E Dahlberg – leif.dahlberg@med.lu.se

Ola Rolfson – ola.rolfson@vgregion.se

Correspondence to Kristin Gustafsson, Unit of Physiotherapy, Department of Health, Medicine and Caring Sciences, Linköping University, Linköping, Sweden. E-mail: kristin.gustafsson@liu.se; kristin.gustafsson@rjl.se

ORCID:

Kristin Gustafsson: 0000-0003-3403-229X

Joanna Kvist: 0000-0003-3527-5488

Marit Eriksson: 0000-0002-1766-5899

Andrea Dell'Isola: 0000-0002-0319-458X

Caddie Zhou: 0000-0002-1669-2318

Leif Dahlberg: 0000-0001-7838-9464

Ola Rolfson: 0000-0001-6534-1242

WORD COUNT
4 059 words

NUMBER OF FIGURES AND TABLES
Two figures and three tables.

For peer review only

ABSTRACT

Objectives To describe the prevalence of comorbidities in a population referred to standardized first-line intervention (patient education and exercise) for hip and knee osteoarthritis (OA), in comparison with the general population. Furthermore, we aimed to evaluate if eventual differences were associated with socioeconomic inequalities.

Design Register-based study.

Setting Primary health care, Sweden.

Participants Individuals with hip and/or knee OA included in the Better Management for Patients with Osteoarthritis Register between 2008 and 2016, and an age-, sex- and residence-matched reference cohort (1:3) from the general Swedish population.

Outcome measures Comorbidities were identified with the RxRisk Index, the Elixhauser Comorbidity Index and the Charlson Comorbidity Index, and presented with descriptive statistics as (1) individual diseases, (2) disease categories and (3) scores for each index. The prevalence of comorbidities in the two populations was tested using logistic regression, with separate analyses for age groups and the most affected joint. We then adjusted the analyses for socioeconomic status.

Results In this OA population, 85% had ≥one comorbidity compared with 78% of the reference cohort (odds ratio (OR) 1.62 (95% confidence interval [CI] 1.59 to 1.66). Cardiovascular/blood diseases were the most common comorbidities in both populations (OA, 59%; reference, 54%), with OR; 1.22 (95% CI 1.20 to 1.24) for the OA population. Younger individuals with OA were more comorbid than their matched references overall, and population differences decreased with age (e.g. ≥3 comorbidities, aged ≤45 years OR; 1.74 (95% CI 1.52 to 1.98, ≥81 years OR; 0.95 (95% CI 0.87 to 1.04). Individuals with knee OA were more comorbid than those with hip OA overall. Adjustment for socioeconomic status did not change the estimates.

Conclusion Comorbidities were more common among individuals with hip and knee OA than among matched references from the general population. The differences could not be explained by socioeconomic status.

Trial registration NCT03438630, posted 20 February 2018.

For peer review only - http://bmjopen.bmj.com/site/about/guidelines.xhtml

Keywords Hip, Knee, Osteoarthritis, Patient education, Exercise, Comorbidity, Registries

Study protocol doi:10.1136/bmjopen-2018-022812

ARTICLE SUMMERY

Strengths and limitations of this study

- This study describes comorbidities in a nationwide OA population in the 3 years before participation in a standardized first-line intervention for hip and knee OA, compared with matched individuals from the general population.
- The study captures data on comorbidities and socioeconomic status from objective data sources, not self-reported data, which gives a good opportunity to adjust for socioeconomic differences.
- Comorbidities are identified with diagnostic codes (ICD-10 codes) and prescription of drugs (ATC codes) using three comorbidity indices: the RxRisk Index, the Elixhauser Comorbidity Index and the Charlson Comorbidity Index, and are also described as individual diseases and as disease categories.
- To determine if socioeconomic inequalities could explain differences in comorbidity between the populations, the results were adjusted for highest educational level, country of birth and marital status.
- In this study we were only able to capture ICD-10 codes from health care delivered through inpatient care and specialized outpatient visits; the ATC codes included data on drugs prescribed in primary health care, in hospitals, by private care givers and occupational health care.

For peer review only - http://bmjopen.bmj.com/site/about/guidelines.xhtml

INTRODUCTION

Osteoarthritis (OA) is one of the most common musculoskeletal disorder in older adults.[1] Approximately 25% of the population more than 45 years old have OA, without accounting for the large numbers who never seek health care for their problem.[2] OA develops slowly over time, leading to pain, joint stiffness and functional limitations.[3]

Co-existence of comorbidities is common among individuals with OA. More than 65% have at least one other chronic condition, which increases with age.[4, 5] The most frequent pattern of OA comorbidities includes cardiovascular and/or metabolic diseases[6] as well as psychological comorbidities such as depressive symptoms.[7, 8] The higher frequency of specific comorbidities in people with OA can be partially explained by an increased vulnerability to develop other diseases because several risk factors, such as female sex, physical inactivity, obesity and lower socioeconomic status (SES), are common to many conditions.[4-6] Comorbidities increase the risk of hospitalization, polypharmacy, premature death and substantially extend the burden on the health care system.[9] In patients with OA, the presence of comorbidities also has impacts on disease severity with increased pain, inferior physical functioning,[10] and worsening of health-related quality of life[11] which may affect the treatment outcome. An increased number of comorbidities has shown to be associated with both a greater detoriation of OA in form of function loss and pain severity,[10] but temporal relationship and causality are yet to be determined.[12]

First-line intervention for hip and knee OA comprises patient education and exercise combined with weight loss when necessary,[13-15] and is recommended regardless of the severity of the disease.[16, 17] This treatment, when delivered in a real-world setting, has shown to alleviate OA symptoms, give patients a satisfactory quality of life, decrease willingness to undergo surgery, reduce use of OA medication, and reduce sick leave.[18]

For peer review only - http://bmjopen.bmj.com/site/about/guidelines.xhtml

However, those results include all individuals, regardless of the prevalence of comorbidities that may affect their outcome. To evaluate and improve treatment outcomes from first-line intervention for hip and knee OA, the magnitude of comorbidities should be described. The aim of the present study was to determine comorbidities, including distribution depending on age, of a population with hip and/or knee OA who were referred to first-line intervention in comparison with the general population. A secondary aim was to evaluate if differences in comorbidities were associated with socioeconomic inequalities.

METHODS

Study design and participants

This observational register-based study included all patients with hip and/or knee OA (n=72 069) in the Better Management of Patients with Osteoarthritis (BOA) national quality register between 2008 and 2016 (table 1). The BOA Register, register and evaluate patients in a standardized national first-line intervention programme for hip and knee OA according to international guidelines,[14, 15] and provided by health professionals in primary care in Sweden.[17] Patients included in the present study were registered before participation (index date) and included regardless of compliance with treatment. They had sought help for hip and/or knee pain in primary health care and had been referred to the first-line intervention programme after a confirmed clinical or radiographic diagnosis of OA following the Swedish National Board of Health and Welfare recommendations.[19-21] Patients with joint problems caused by another condition (e.g. sequelae of hip fractures, chronic widespread pain, inflammatory joint diseases or cancer), patients who have undergone total joint replacement within the previous 12 months or other surgery of the knee or hip joint within the previous 3 months do not meet the criteria for inclusion and are therefore excluded from registration in the BOA Register.[17] The standardized first-line intervention for OA, the development of the

For peer review only - http://bmjopen.bmj.com/site/about/guidelines.xhtml

BOA Register and the inclusion and exclusion criteria for registration of patients in the register have been described in detail previously.[17, 22]

Without involvement from the researchers, Statistics Sweden (a government agency) randomly selected a reference cohort (n=216 207) of individuals from the general Swedish population for comparison using the Swedish Total Population Register (TPR)[23] (table 1). Those individuals had never been included in the BOA Register. To minimize confounding due to differences in access to care, the populations were matched for year of birth, sex and place of residence (counties in Sweden, n=21), at the index date in the BOA Register. Three reference individuals were identified for each patient in the OA population. The study followed the recommendations according to Strengthening the Reporting of Observational Studies in Epidemiology (STROBE).[24]

For peer review only

Table 1 Characteristics of the OA population from the BOA Register and the reference cohort and differences in comorbidities between the populations identified by the comorbidity indices

	OA population (n=72 069)	Reference cohort (n=216 207)
**Age** (years) mean (SD)	66.4 (9.6)	66.4 (9.6)
**Women**, % (n)	69 (49 494)	69 (148 482)
**Most affect joint in the OA population**, % (n)		
Hip	32 (22 703)	
Knee	68 (49 366)	
**OA in multiple joints,** [a] % (n)	62 (44 384)	
Missing	*n=443*	
**Born outside Sweden**, % (n)	9 (6474)	13 (28 554)
Missing	*n=0*	*n=12*
**Married**, % (n)	59 (42 359)	54 (117 616)
Missing	*n=9*	*n=162*
**Educational level**, % (n)		
Low (≤9 years)	22.5 (16 276)	28.5 (61 212)
Medium (10-14 years)	60.5 (43 492)	56 (119 416)
High (≥ 15 years)	17 (12 111)	15.5 (33 260)
Missing	*n=190*	*n=2 319*
**Comorbidities indices**		
Missing	*n=0*	*n=0*
**RxRisk Index**[b] median (IQR)	3 (4)	2 (4)
Min-max score	0-18	0-18
0 (no comorbidities), % (CI)[c]	16 (15.7, 16.3)	23 (22.8, 23.2)
1-3	44 (43.6, 44.4)	40.5 (40.3, 40.7)
≥4	40 (39.6, 40.4)	36.5 (36.3, 36.7)
**Elixhauser Comorbidity Index**, median (IQR)	0 (1)	0 (1)
Min-max score	0-11	0-13
0 (no comorbidities), % (CI)[b]	69.5 (69.2, 69.8)	69 (68.8, 69.2)
1	16.5 (16.2, 16.8)	16 (15.8, 16.2)
≥2	14 (13.7, 14.3)	15 (14.8, 15.2)
**Charlson Comorbidity Index**, median (IQR)	0 (0)	0 (0)
Min-max score	0-11	0-15
0 (no comorbidities), % (CI)[b]	81 (80.7, 81.3)	78 (77.8, 78.2)
1	9.5 (9.3, 9.7)	10 (9.9, 10.1)
≥2	9.5 (9.3, 9.7)	12 (11.9, 12.1)
**Comorbidities (≥1)** grouped by age, % (CI)[b]		
<45 years (OA n=1583; ref n=4749)	65 (62.7, 67.3)	51 (49.6, 52.4)
46–65 years (OA n=29 225; ref n=87 675)	79 (78.5, 79.5)	69 (68.7, 69.3)
66–80 years (OA n=36 993; ref n=110 979)	90 (89.7, 90.3)	84.5 (84.3, 84.7)
>81 years (OA n=4268; ref n=12 804)	96 (95.4, 96.6)	94 (93.6, 94.4)

OA, osteoarthritis; SD, standard deviation; n, numbers; IQR, interquartile range; CI, confidence interval.

For peer review only - http://bmjopen.bmj.com/site/about/guidelines.xhtml

[a]Osteoarthritis in multiple joints, identified as B or C Charley Class, a self-reported classification of musculusceletal impairment. Class A indicates unilateral hip or knee OA, Class B; bilateral hip or knee OA and class C; multiple joint OA or some other condition affecting the patient's ability to walk.

[b]The conditions "inflammation/pain" and "pain" are excluded from the score count.

[c]Percentage with 95% confidence interval (CI) for group comparison of proportions.

Data source

By using the 10-digit personal identity number (PIN), a unique number assigned to all Swedish residents at birth or immigration,[25] data on comorbidities from the index date and 3 years before that date, were obtained from two nationwide individual-based registers at the National Board of Health and Welfare: (1) the Swedish National Inpatient Register, which has an overall positive predictive value for diagnoses of 85%–95%; and (2) the Swedish Prescribed Drug Register, with data on expenditure of prescribed drugs in the entire Swedish population.[26, 27] Socioeconomic individual-level data for the same year as the index date were obtained from the TPR and the Longitudinal Integration Database for Health Insurance and Labour Market Studies (LISA) at Statistics Sweden.[23, 28] Only the PIN and data on the most affected joint (hip or knee) were extracted from the BOA Register. Details of the different registers and the linkage of data between them, together with the creation and matching of a reference cohort from the general Swedish population, as well as a flowchart of the study participants from the BOA Register and their matched references have been described previously.[22, 29]

Identification of comorbidities

When describing the OA population and the reference cohort, we use the term comorbidities throughout the present study, defined as "presence of an additional disease in relation to an index disease in one individual", even though a more correct definition regarding the reference cohort would be morbidity or multimorbidities.[30] Comorbidities

For peer review only - http://bmjopen.bmj.com/site/about/guidelines.xhtml

were identified using three different comorbidity indices: (1) the RxRisk Index,[31] (2) the Charlson Comorbidity Index[32] and the Elixhauser Comorbidity Index,[33] and are presented as (1) individual diseases, (2) disease categories and (3) scores for each of the indices. Together, the three indices identified 66 individual diseases, which were grouped into 11 different disease categories (table 2).[34, 35] When the scores for the indices were calculated, the unweighted versions of all three indices were used; for example, a score of 2 indicates that the patient had two comorbidities.[31, 36, 37]

The RxRisk Index uses data on prescriptions of drugs to identify the presence of 43 individual diseases (table 2) based on the Anatomical Therapeutic Chemical (ATC) classification system of the drugs, defined according to the World Health Organization.[31] In this study, data from the Swedish Prescribed Drug Register were used, and the specific ATC codes for each of the 43 comorbidities are presented in Supplementary file 1. Scores for the index were categorized as 0, 1–3, or ≥4 comorbidities.

To calculate the Charlson Comorbidity Index[32] and the Elixhauser Comorbidity Index,[33] we used data on International Classification of Diseases, Tenth Revision (ICD-10) codes from the Swedish National Inpatient Register. The Charlson Comorbidity Index identifies 17 comorbidities and the Elixhauser Comorbidity Index identifies 30 comorbidities (table 2, supplementary file 2). Scores for both indices were categorized as 0, 1 or ≥2 comorbidities.

For peer review only - http://bmjopen.bmj.com/site/about/guidelines.xhtml

Table 2 Comorbidities of patients with osteoarthritis (OA) from the BOA Register, compared with the general population

			**RxRisk Index**		**Elixhauser Comorbidity Index**		**Charlson Comorbidity Index**	
	OA	Ref	OA	Ref	OA	Ref	OA	Ref
**Cancer**	**6%**	**6%**						
Lymphoma					0.5%	0.5%		
Malignancies			0.5%	0.5%			5.5%	6%
Metastatic cancer					0.5%	1%	0.5%	1%
Solid tumour without metastasis					5%	5.5%		
**Cardiovascular/blood**	**59%**	**54%**						
Anticoagulation agents/coagulopathy			10%	9%	0.5%	0.5%		
Antiplatelet agents			19%	19%				
Arrhythmias			2%	2%	6%	6%		
Cerebrovascular disease							2.5%	3.5%
Congestive heart failure			21.5%	21%	1.5%	2.5%	1.5%	2.5%
Hyperlipidaemia			28%	26.5%				
Hypertension			31%	27%	15%	14.5%		
Ischaemic heart disease: angina			7%	6.5%				
Ischaemic heart disease: hypertension			34%	32%				
Myocardial infarction							2.5%	3%
Peripheral vascular disease					1%	1.5%	1%	1.5%
Pulmonary circulation disorders					0.5%	0.5%		
Pulmonary hypertension			0%	0%				
Valvular disease					1.5%	1.5%		
**Endocrine**	**19%**	**19%**						
Diabetes (uncomplicated)			9%	10%	4.5%	5%	4.5%	5%
Diabetes (complicated)					1.5%	2%	1.5%	2%
Hyperthyroidism			0%	0%				
Hypothyroidism			10.5%	9.5%	2%	2%		
Pancreatic insufficiency			0.5%	0.5%				
**Gastrointestinal**	**35%**	**28%**						
Gastroesophageal reflux disease			32.5%	25%				
Irritable bowel syndrome			1.5%	1%				
Liver disease (mild)					0.5%	0.5%	0.5%	0.5%
Liver disease (severe) or failure			4%	4.5%			0%	0%
Peptic ulcer disease					0.5%	0.5%	0.5%	0.5%
**Musculoskeletal/pain related**	***70%***	***45%***						
**- Excl. *pain* and *inflammation/pain***	**11%**	**10%**						
Gout			2.5%	2.5%				
Migraine			2.5%	2%				
Osteoporosis/Paget's			4.5%	4.5%				
Pain			*31.5%*	*23%*				
Inflammation/pain			*58.5%*	*31.5%*				
Rheumatic disease							1.5%	2%
Rheumatoid arthritis /collage vascular diseases					2%	2.5%		

For peer review only - http://bmjopen.bmj.com/site/about/guidelines.xhtml

			RxRisk Index		Elixhauser Comorbidity Index		Charlson Comorbidity Index	
	OA	Ref	OA	Ref	OA	Ref	OA	Ref
**Neurologic**	**7%**	**8%**						
Dementia			0%	1%			0%	1%
Epilepsy			4%	4.5%				
Paralysis					0%	0.5%		
Hemiplegia or paraplegia							0%	0.5%
Parkinson's disease			2.5%	2%				
Other neurologic disorders					1%	2%		
**Nutritional/obesity**	**3%**	**3%**						
Blood loss anaemia					0%	0%		
Deficiency anaemia					0.5%	0.5%		
Fluid and electrolyte disorders					0.5%	1%		
Hyperkalaemia			0%	0%				
Malnutrition			0%	0%				
Obesity					2%	1%		
Weight loss					0%	0%		
**Psychological/behavioural**	**23%**	**24%**						
Alcohol abuse/dependency			0.5%	0.5%	0.5%	1%		
Anxiety			8.5%	10%				
Bipolar disorder			0.5%	0.5%				
Depression			17.5%	17.5%	2%	2.5%		
Drug abuse					0%	0.5%		
Psychotic illness/psychoses			0.5%	1.5%	0%	0.5%		
Smoking cessation			1.5%	1.5%				
**Renal/urologic**	**9%**	**7%**						
Benign prostatic hyperplasia			4.5%	3.5%				
Incontinence			4.5%	3%				
Renal disease/failure			0.5%	0.5%	0.5%	1%	0.5%	1%
**Respiratory**	**12%**	**11%**						
Chronic pulmonary disease					3%	3.5%	3%	3.5%
Chronic airways disease			11%	9.5%				
**Miscellaneous**	**37%**	**31%**						
Allergies			24%	19.5%				
Glaucoma			4%	4%				
HIV/AIDS			0%	0%	0%	0%	0%	0%
Psoriasis			1.5%	1.5%				
Steroid-responsive conditions			16.5%	13.5%				
Transplant			0%	0%				

Comorbidities presented as disease categories and as individual comorbidities, identified by the RxRisk Index, the Elixhauser Comorbidity Index and the Charlson Comorbidity Index, for the OA population (n= 72 069) compared with their references from the general population (n=216 207). All numbers are reported as percentages (%). The numbers marked in bold are the percentage of each population that has been identified with at least one of the individual diseases included in that disease

For peer review only - http://bmjopen.bmj.com/site/about/guidelines.xhtml

category. The other numbers are the percentage of each population that has been identified with each individual disease by each of the indices.

For peer review only

For peer review only - http://bmjopen.bmj.com/site/about/guidelines.xhtml

Indicators of socioeconomic status

Highest educational level achieved, country of birth and marital status were used as proxies for SES. Education was divided into three categories: low (≤primary school, 0–9 years), medium (secondary school plus up to <3 years postsecondary education, 10–14 years) and high (postsecondary education ≥3 years, ≥15 years). Country of birth was categorized as Sweden, the Nordic countries, Europe or others, and marital status as married (including registered partner) or not married.[29]

Statistical analysis

Comorbidities in the OA population compared with the reference cohort are described using frequencies, proportions) or medians and interquartile ranges (IQRs) depending on the nature of the data. The OA population was also analysed separately grouped by age (<45, 46–65, 66–80 and >81 years) and by most affected joint (hip or knee) and compared with their respective matched references.

Two conditions that were identified by the RxRisk Index, inflammation/pain (treated with anti-inflammatory medication) and pain (treated with narcotics), were excluded in all calculations of the RxRisk Index, and from the analyses of the disease category, musculoskeletal and pain-related diseases, because they were considered as index diseases for this OA population.

Logistic regression models were used to evaluate the eventual effect of SES on the prevalence of comorbidities. Both crude and adjusted analyses were performed for socioeconomic indicators (educational level, country of birth and marital status), calculating the odds ratio (OR) with the 95% confidence interval (CI). For those who were included in the BOA Register up to 2015 and their matched references, we also had access to data on

For peer review only - http://bmjopen.bmj.com/site/about/guidelines.xhtml

other socioeconomic indicators (income, employment and family type). Secondary analyses were therefore performed to evaluate if those indicators affected the results, but because they did not, we chose only to use those indicators for SES that we had access to for the total study population (presented in Supplementary file 3). .

Nearly two-thirds of the OA population but only one-third of their references had prescriptions for anti-inflammatory medications (ATC codes M01A–M01H), drugs that increase the risk of developing gastrointestinal comorbidities,[38] and thereby often are prescribed preventively in combination with gastro-protective drugs (ATC codes A02BA01–A02BX05). Therefore, we also chose to do separate analyses of those who were not using anti-inflammatory drugs to try to establish the real prevalence of gastroesophageal diseases.

The merging of data from the National Board of Health and Welfare and the creation of the database were performed using SAS 9.4 TS Level 1MS. All statistical analyses were performed with IBM SPSS Statistics for Windows, v25.0 (IBM Statistics, Armonk, NY).

Patient and public involvement

Patients were not involved in the development of the research question or the design of this study.

Ethical approval and trial registration

The study was approved by the Regional Ethical Review Board in Gothenburg, Sweden (entry number 1059-16), and the study was registered at clinicaltrials.gov, with trial registration number NCT03438630. The study followed the legal and ethical frameworks of informed consent in register-based research, as described by Swedish law and ethical boards.[39] All patients in the BOA Register received oral and written information about their registration, including information that data may be used in research. Regarding the reference

For peer review only - http://bmjopen.bmj.com/site/about/guidelines.xhtml

cohort, informed consent was waived according to standard practice in Sweden, since no individuals in this population-based cohort could be identified. Statistics Sweden replaces each PIN with a serial number to anonymize data before returning it to the researchers.

RESULTS

Study participants

The characteristics of the participants in the study are described in table 1. The mean age was 66.4 years (SD, 9.6 years) and 69% were women. In the OA population, 32% were registered with OA most affecting the hip joint and 68% most affecting the knee joint.

Prevalence of comorbidities

A higher proportion of individuals in the OA population (85%) had comorbidities than in the reference cohort; 78% of the references were identified with at least one comorbidity. Of the 66 comorbidities that could be traced, 23 had a low prevalence with ≤1% in each of the populations (table 2). Cardiovascular/blood diseases were the most common comorbidities in the disease category, with 59% of this OA population and 54% of their references identified with at least one of those, followed by gastrointestinal diseases (35% of OA and 28% of the references).

In the OA population, 32.5% were identified (by the RxRisk Index) as having medication prescribed for gastroesophageal reflux disease compared with 25% of the reference cohort. Separate analyses excluding those without concomitant treatments with anti-inflammatory medications showed that the prevalence of gastroesophageal reflux disease evened out between the two populations, but was still higher in the OA population (24%) than among the references (19%).

For peer review only - http://bmjopen.bmj.com/site/about/guidelines.xhtml

A fourth of both populations had psychological/behavioural comorbidities; depression (17.5% in both populations) and anxiety (8.5% in the OA population and 10% in the reference cohort) were the most common with a similar representation in both populations (table 2). Also the prevalence of endocrine diseases such as diabetes was equally distributed in the two populations (19%).

When scores were calculated for each of the three indices, the OA population was identified with more comorbidities by the RxRisk Index, compared with their references; with the Elixhauser Comorbidity Index, the prevalence of comorbidities was similar between the two populations; and with the Charlson Comorbidity Index, the OA population showed a slightly lower prevalence of comorbidities compared with the reference cohort (table 1).

Associations between the populations and comorbidities

The OA population had a higher risk of having at least one other comorbidity (OR, 1.62; 95% CI 1.59 to 1.66), but the odds then decreased for additional comorbidities, with an OR of 1.36 (95% CI 1.34 to 1.39) for having ≥2 and 1.23 (95% CI 1.21 to 1.26) for having ≥3 comorbidities. Adjustment for SES led to a slightly higher OR overall than the crude value (table 3).

Being part of this OA population was also associated with a higher odds of having comorbidities in several of the disease categories (cardiovascular/blood, endocrine, gastrointestinal, renal/urologic, respiratory and miscellaneous), but also statistically significant lower ORs for cancer, neurologic, nutritional/obesity and psychological/behavioural diseases. The results when adjusted for SES were similar to the crude ORs (figure 1, table 3).

For peer review only - http://bmjopen.bmj.com/site/about/guidelines.xhtml

For peer review only

Table 3 Odds ratio (OR) of having comorbidities for the OA population in the BOA Register compared with the reference cohort

	Total study population		Hip OA		Knee OA	
	**Crude OR** (95% CI)	**Adjusted OR** (95% CI)[a]	**Crude OR** (95% CI)	**Adjusted OR** (95% CI)[a]	**Crude** (95% CI)	**Adjusted OR** (95% CI)[a]
**Individual comorbidities**[b]						
≥1	1.62 (1.59, 1.66)*	1.67 (1.63, 1.71)*	1.48 (1.42, 1.54)*	1.53 (1.46, 1.59)*	1.69 (1.65, 1.74)*	1.73 (1.69, 1.78)*
≥2	1.36 (1.34, 1.39)*	1.41 (1.38, 1.44)*	1.26 (1.22, 1.30)*	1.31 (1.27, 1.35)*	1.41 (1.38, 1.45)*	1.46 (1.43, 1.49)*
≥3	1.23 (1.21, 1.26)*	1.28 (1.26, 1.31)*	1.14 (1.11, 1.17)*	1.19 (1.16, 1.23)*	1.28 (1.25, 1.31)*	1.33 (1.30, 1.36)*
**Disease categories**						
**Cancer**	0.89 (0.86, 0.92)*	0.89 (0.85, 0.92)*	0.90 (0.85, 0.96)*	0.89 (0.84, 0.95)*	0.88 (0.84, 0.92)*	0.88 (0.84, 0.92)*
**Cardiovascular/ blood**	1.22 (1.20, 1.24)*	1.26 (1.24, 1.28)*	1.15 (1.11, 1.18)*	1.19 (1.15, 1.23)*	1.26 (1.23, 1.29)*	1.29 (1.27, 1.32)*
**Endocrine**	1.04 (1.02, 1.06)*	1.09 (1.06, 1.11)*	0.97 (0.93, 1.01)	1.02 (0.98, 1.06)	1.08 (1.05, 1.10)*	1.12 (1.09, 1.15)*
**Gastrointestinal**	1.38 (1.35, 1.40)*	1.43 (1.41, 1.46)*	1.31 (1.27, 1.35)*	1.37 (1.33, 1.42)*	1.41 (1.38, 1.44)*	1.46 (1.43, 1.50)*
**Musculoskeletal/ pain-related**[c]	1.03 (1.00, 1.06)	1.04 (1.01, 1.07)*	1.02 (0.97, 1.07)	1.02 (0.97, 1.08)	1.04 (1.00, 1.07)	1.04 (1.01, 1.08)
**Neurologic**	0.79 (0.76, 0.82)*	0.82 (0.79, 0.84)*	0.74 (0.69, 0.78)*	0.76 (0.71, 0.80)*	0.82 (0.78, 0.85)*	0.84 (0.81, 0.88)*
**Nutritional/obesity**	0.92 (0.88, 0.97)*	0.97 (0.92, 1.02)	0.75 (0.68, 0.83)*	0.79 (0.72, 0.87)*	1.01 (0.95, 1.07)	1.06 (0.99, 1.12)
**Psychological/ behavioural**	0.96 (0.94, 0.98)*	0.99 (0.97, 1.01)	0.94 (0.90, 0.97)*	0.97 (0.93, 1.00)	0.96 (0.94, 0.99)*	1.00 (0.97, 1.02)
**Renal/urologic**	1.30 (1.26, 1.34)*	1.32 (1.28, 1.36)*	1.24 (1.18, 1.31)*	1.27 (1.20, 1.34)*	1.33 (1.29, 1.38)*	1.34 (1.30, 1.40)*
**Respiratory**	1.16 (1.13, 1.19)*	1.18 (1.14, 1.21)*	1.11 (1.06, 1.16)*	1.13 (1.08, 1.18)*	1.18 (1.15, 1.22)*	1.20 (1.16, 1.24)*
**Miscellaneous**	1.29 (1.27, 1.31)*	1.29 (1.27, 1.31)*	1.23 (1.19, 1.27)*	1.22 (1.19, 1.26)*	1.32 (1.29, 1.35)*	1.32 (1.29, 1.35)*

OA, osteoarthritis; OR, odds ratio; CI, confidence interval.

[a]Adjusted for socioeconomic status (educational level, country of birth and marital status).

[b]Individual comorbidities identified by summing the RxRisk Index, the Elixhauser Comorbidity Index and the Charlson Comorbidity Index.

For peer review only - http://bmjopen.bmj.com/site/about/guidelines.xhtml

[c]The conditions inflammation/pain and pain identified by the Rx Risk Index were excluded from the analyses.

*Statistically significant results.

Separate analyses of subgroups

Grouping the populations by age, the OA population had a higher presence of comorbidities (≥1) in the younger age groups compared with their matched references, whereas in the oldest age group, the prevalence were similar in the two populations (table 1). The OA population also showed an overall higher odds of having one or more comorbidities for all age groups, the younger, the higher ORs. There were no differences in OR for ≥1, ≥2 or ≥3 comorbidities in the youngest age group (aged <45 years). In contrast, the ORs for ≥2 or ≥3 comorbidities were continuously lower than having only ≥1 comorbidity in the older age groups (aged 46–>81 years) (figure 2).

When grouping the OA population by most affected joint (hip/knee) and then comparing them with their respective matched references, those registered with knee OA had more comorbidities than those registered with hip OA (table 3). Also in the separate analyses of the populations in subgroups, adjustment for SES had only a minor effect on the estimates (figure 2, table 3).

DISCUSSION

This study shows that individuals referred to a standardized national first-line intervention for OA in Sweden had an overall higher prevalence of comorbidities than their matched references from the general population. The study also shows that there was a higher prevalence of comorbidities among younger individuals in the OA population compared with their references, whereas the prevalence was similar for older individuals, which could indicate that it is probably important to initiate first-line intervention early to reduce the risk

For peer review only - http://bmjopen.bmj.com/site/about/guidelines.xhtml

of developing related disease.[17] Patients in the OA population registered with knee OA had more comorbidities than those registered with hip OA. This may be partly explained by the fact that obesity is more common among those registered with knee OA compared with hip OA in the BOA Register,[18] which is also a risk factor for the development of several other diseases.[5, 6]

Previously, we have shown an overall higher SES in this OA population from the BOA Register compared with that of the general Swedish population.[29] From the results in the present study, we can now report that the inequalities in SES were not associated with differences in comorbidities, but must be related to other factors. It is well known that there is a connection between SES and several common chronic diseases, with poorer health among more socioeconomically disadvantaged individuals,[40, 41] and that OA is in general more common among individuals with lower SES.[42, 43] The reasons why this OA population had a higher SES, but had more comorbidities than the general population, are probably highly dependent on the association that exists between OA and other diseases.[4] In addition, it could also be the effect of individuals' lifestyle and health care seeking behaviours; individuals with lower SES tends to seek health care less often, which could result in both late diagnosis and delayed treatment.[44] It has also been shown previously that more disadvantaged individuals with OA have poorer access to both nonsurgical health care services, such as self-management education, physiotherapy and medication, and to joint replacement surgery.[45, 46] In the present study, the OA population showed a higher prevalence of comorbidities compared with the reference cohort, and most comorbidities for both populations were detected from data on prescriptions of drugs and by the RxRisk Index, whereas data from ICD-10 codes for inpatient care showed slightly more comorbidities among the reference cohort. It could be argued that the BOA population probably to a higher extent includes individuals who take responsibility for their health, who seek help for their

For peer review only - http://bmjopen.bmj.com/site/about/guidelines.xhtml

OA problems and at the right level of care, and who take the medications that are prescribed for them, and that they are thus also more represented and appear in registers that include prescription of drugs.

The differences in comorbidities between the populations in the present study were not of the same magnitude as in a recent review by Swain et al.[4] They reported that 67% of individuals with OA were identified with ≥1 other chronic condition, which was 20% higher than among non-OA individuals. Pihl et al.[47] identified self-reported comorbidities among approximate 50% of a similar Danish OA population, before participation in a first-line intervention programme for hip and knee OA, and that the presence of comorbidities were not associated with outcomes after the intervention. However, differences in prevalence of comorbidities between studies may be due to several factors, making comparison challenging, such as different study designs, the sample characteristics, methods of identifying comorbidities and the number of comorbidities analysed. Knowledge of how OA and comorbidities associate is still largely lacking and further research is needed.[4] Instead of comparing the overall prevalence of comorbidities in this OA population with results from other studies, it may be more interesting to compare prevalence divided into different groups of diseases. In the present study, we identified that cardiovascular/blood diseases was the most common disease category in both populations, with a higher prevalence in the OA population compared with the reference cohort. These results are supported in a previous review by Hall et al.,[48] who established that individuals with OA had a significantly higher prevalence of cardiovascular diseases, especially heart failure and ischaemic heart disease, compared with matched controls. The prevalence of diabetes[49] and depression[7] has also been shown to be higher in OA populations compared with general populations. Those results were not supported by this study; we instead identified similar prevalence in both populations. Our separate analyses excluding individuals with an anti-inflammatory prescription showed

For peer review only

For peer review only - http://bmjopen.bmj.com/site/about/guidelines.xhtml

that the difference in the prevalence of gastroesophageal reflux disease was reduced but remained higher in this OA population. This may be due to greater use of over-the-counter anti-inflammatory medication, which can be assumed to be higher among people with OA. The odds of having cancer or neurologic diseases was lower in the OA population than the reference cohort, but those results are probably the effect of the criteria for registration in the BOA Register, which excludes individuals with OA who also have other severe diseases.[17]

In the present study, we used three comorbidity indices to make it possible to identify several diseases, and from different data sources. The Charlson Comorbidity Index and the Elixhauser Comorbidity Index were developed to capture comorbidities in inpatient care using diagnostic ICD-10 codes,[36] and are commonly used in orthopaedics research,[34] whereas the RxRisk Index identifies comorbidities from prescription of drugs by ATC codes and can be used in predominately outpatient settings,[31] such as this specific OA population. All three indices have been used previously to study comorbidities in OA populations.[34, 35] The possibility to use comorbidity indices and summarize them into scores is necessary when handling large administrative datasets, but there is no gold standard with regard to which index should be used primarily. Which method that is best suited to describe comorbidities depends on the circumstances, and some comorbidities are better detected using medication-based data and others with diagnosis-based data.[34, 35] For example; the diagnose diabetes, can by the Charlson Comorbidity Index and the Elixhauser comorbidity index also include individuals who are treated without diabetes medication, which is not the case with the RxRisk index. With the Charlson Comorbidity Index, complications in patients with diabetes should be chronical, but not necessarily so according to Elixhauser comorbidity index.[36] Therefore, even if the same comorbidities are identified by different indices, they should not be compared between the different indices, only between different populations. In the present study with an aim to describe the magnitude of different comorbidities, we chose to combine

For peer review only - http://bmjopen.bmj.com/site/about/guidelines.xhtml

the three indices to capture the overall burden of the comorbidities, instead of only focusing on separate scores for each index.

Limitations and strengths

There are some limitations in the present study that should be highlighted. To seek health care for a condition like OA may increase the probability to also be registered for other diagnoses, and/or receiving prescriptions of drugs for other conditions. Obesity increases the risk of developing several chronic conditions including OA[50] and may be a confounding factor of the results. Since we only had access to data on body mass index for the OA population, but not for the reference cohort, we could not adjust for obesity. We were only able to capture ICD-10 codes to calculate the Charlson Comorbidity Index and the Elixhauser Comorbidity Index from health care delivered through inpatient care and specialized outpatient visits. Data on primary health care and information on health care provided by professionals other than physicians are not available on a national level in Sweden. However, both the Charlson Comorbidity Index and the Elixhauser Comorbidity Index were developed primarily to capture comorbidities in inpatient care.[36]

Another limitation to bear in mind when interpreting the results from the present study is that the criteria for registration in the BOA Register exclude individuals who have other severe diseases.[17] This may contribute to underestimating comorbidities, because we can assume that this OA population probably has less comorbidities than general OA populations who are at the same stage of their disease, when seeking health care and structured OA treatments, such as the OA population in the present study. The contradictive results that the Charlson Comorbidity Index displayed a slightly higher prevalence of comorbidities in the reference cohort could also be an effect of this exclusion of indvidiuals with severer diseases in the BOA Register, since the diseases detected by Charlson consistently are of a somewhat

For peer review only - http://bmjopen.bmj.com/site/about/guidelines.xhtml

more serious nature. In addition, there could be individuals with OA in the reference cohort, which may affect the results; OA is common in those age groups, it is difficult to define the onset of the disease,[51] many individuals with OA symptoms do not seek health care[52] or are not referred to first-line intervention for OA. For example, it has been estimated that during 2014, the BOA Register reached 17% of all individuals who sought outpatient care with the diagnosis of hip or knee OA.[53]

Despite these circumstances, we could identify a higher prevalence of several diseases in the OA population compared with the age-, sex- and residence-matched reference cohort. The results from the present study are applicable on OA individuals referred to first-line intervention, but are also in line with several previous studies,[4, 5, 48] describing comorbidities among individuals with OA compared with general populations.

Even though more recent guidelines on OA treatment include recommendations for individuals with comorbidities,[15] they are often excluded from exercise treatment,[54] with the risk of being left without any treatment. However, OA is not a single-joint disease and to improve treatment outcomes from first-line intervention for hip and knee OA, it is important to not only focus on specific OA treatments but also on increased overall health.

CONCLUSION

Comorbidities were more common among individuals referred to first-line intervention for hip and knee OA than among a matched reference cohort from the general population, especially among younger individuals and those with OA most affecting their knees. Based on the results from this study, socioeconomic status does not seem to affect the differences in comorbidities detected between this OA population and the reference cohort.

For peer review only - http://bmjopen.bmj.com/site/about/guidelines.xhtml

ACKNOWLEDGEMENTS

The authors would like to acknowledge all participating patients, physiotherapists reporting data to the BOA Register, and others involved in BOA.

Competing interests

LED is the co-founder and Chief Medical Officer of Joint Academy, a company that provides digital first-line intervention for patients with hip and knee OA. The other authors declare that they have no conflicts of interest.

Author contributions

KG, JK, ME, and OR designed the study. KG and OR contributed to acquisition of data. CZ were responsible for merging of data from the BOA Register with data from the National Board of Health and Welfare and Statistics Sweden and for the creation of the research database. KG had full access to all data that was analysed in the study and takes, together with CZ, responsibility for the accuracy of the data analysis. All authors (KG, JK, ME, ADI, CZ, LED and OR) contributed in interpretation of data. KG wrote the first draft of the paper, which was critically revised by the other authors. All authors interpreted the findings and approved the final version of the manuscript before submission.

Funding statement

The study was financially supported by AFA Insurance, Sweden, Futurum–Academy for Health and Care, Region Jönköping County, Sweden and the Medical Research Council of Southeast Sweden. The funders had no influence on the design or the conduct of the study, the analysis or interpretation of the data or the writing of the manuscript.

For peer review only - http://bmjopen.bmj.com/site/about/guidelines.xhtml

Data sharing statement

The dataset supporting the conclusions of this article is governed by Västra Götalandsregionen. The authors are not allowed to share the data. The data are available from Registercentrum, Västra Götalandsregionen (contact; boa@registercentrum.se) for researchers who meet the criteria for access to confidential data according to Swedish law.

REFERENCES

1 Vos T, Abajobir A, Abate K, et al. Global, regional, and national incidence, prevalence, and years lived with disability for 328 diseases and injuries for 195 countries, 1990-2016: a systematic analysis for the Global Burden of Disease Study 2016. *Lancet* 2017;390(10100):1211-59.

2 Turkiewicz A, Petersson IF, Bjork J, et al. Current and future impact of osteoarthritis on health care: a population-based study with projections to year 2032. *Osteoarthritis Cartilage* 2014;22(11):1826-32.

3 Hunter DJ, Bierma-Zeinstra S. Osteoarthritis. *Lancet* 2019;393(10182):1745-59.

4 Swain S, Sarmanova A, Coupland C, et al. Comorbidities in Osteoarthritis: A Systematic Review and Meta - Analysis of Observational Studies. *Arthritis Care & Research* 2020;72(7):991-1000.

5 Kadam UT, Jordan K, Croft PR. Clinical comorbidity in patients with osteoarthritis: a case-control study of general practice consulters in England and Wales. *Ann Rheum Dis* 2004;63(4):408-14.

6 Violan C, Foguet-Boreu Q, Flores-Mateo G, et al. Prevalence, determinants and patterns of multimorbidity in primary care: a systematic review of observational studies. *PLoS One* 2014;9(7):e102149.

7 Veronese N, Stubbs B, Solmi M, et al. Association between lower limb osteoarthritis and incidence of depressive symptoms: data from the osteoarthritis initiative. *Age Ageing* 2017;46(3):470-76.

For peer review only - http://bmjopen.bmj.com/site/about/guidelines.xhtml

8 Wilkie R, Kaur K, Hayward RA. The impact of socioeconomic status on the link between osteoarthritis and the onset of common comorbidities. *Clin Exp Rheumatol* 2019;37(1):44-48.

9 Smith SM, Soubhi H, Fortin M, et al. Managing patients with multimorbidity: systematic review of interventions in primary care and community settings. *Bmj* 2012;345:e5205.

10 Calders P, Van Ginckel A. Presence of comorbidities and prognosis of clinical symptoms in knee and/or hip osteoarthritis: A systematic review and meta-analysis. *Semin Arthritis Rheum* 2018;47(6):805-13.

11 Geryk LL, Carpenter DM, Blalock SJ, et al. The impact of co-morbidity on health-related quality of life in rheumatoid arthritis and osteoarthritis patients. *Clin Exp Rheumatol* 2015;33(3):366-74.

12 Swain S, Coupland C, Mallen C, et al. Temporal relationship between osteoarthritis and comorbidities: a combined case control and cohort study in the UK primary care setting. *Rheumatology (Oxford)* 2021

13 Verhagen AP, Ferreira M, Reijneveld-van de Vendel EAE, et al. Do we need another trial on exercise in patients with knee osteoarthritis?: No new trials on exercise in knee OA. *Osteoarthritis Cartilage* 2019;27(9):1266-69.

14 Kolasinski SL, Neogi T, Hochberg MC, et al. 2019 American College of Rheumatology/Arthritis Foundation Guideline for the Management of Osteoarthritis of the Hand, Hip, and Knee. *Arthritis Rheumatol* 2020;72(2):220-33.

15 Bannuru RR, Osani MC, Vaysbrot EE, et al. OARSI guidelines for the non-surgical management of knee, hip, and polyarticular osteoarthritis. *Osteoarthritis Cartilage* 2019;27(11):1578-89.

16 Zhang W, Nuki G, Moskowitz RW, et al. OARSI recommendations for the management of hip and knee osteoarthritis: part III: Changes in evidence following systematic cumulative update of research published through January 2009. *Osteoarthritis Cartilage* 2010;18(4):476-99.

For peer review only

For peer review only - http://bmjopen.bmj.com/site/about/guidelines.xhtml

17 Thorstensson CA, Garellick G, Rystedt H, et al. Better Management of Patients with Osteoarthritis: Development and Nationwide Implementation of an Evidence-Based Supported Osteoarthritis Self-Management Programme. *Musculoskeletal Care* 2015;13(2):67-75.

18 Jonsson T, Eek F, Dell'Isola A, et al. The Better Management of Patients with Osteoarthritis Program: Outcomes after evidence-based education and exercise delivered nationwide in Sweden. *PLoS One* 2019;14(9):e0222657.

19 Altman R, Alarcon G, Appelrouth D, et al. The American College of Rheumatology criteria for the classification and reporting of osteoarthritis of the hip. *Arthritis Rheum* 1991;34(5):505-14.

20 Altman R, Asch E, Bloch D, et al. Development of criteria for the classification and reporting of osteoarthritis. Classification of osteoarthritis of the knee. Diagnostic and Therapeutic Criteria Committee of the American Rheumatism Association. *Arthritis Rheum* 1986;29(8):1039-49.

21 Socialstyrelsen. Nationella riktlinjer för rörelseorganens sjukdomar 2012: Osteoporos, artros, inflammatoriskryggsjukdom och ankyloserande spondylit, psoriasisartrit och reumatoid artrit: Stöd för styrning och ledning [Swedish]. Stockholm; Socialstyrelsen; 2012.

22 Gustafsson K, Rolfson O, Eriksson M, et al. Study protocol for an observational register-based study on health and risk factors in patients with hip and knee osteoarthritis. *BMJ Open* 2018;8(10):e022812.

23 Ludvigsson JF, Almqvist C, Bonamy AK, et al. Registers of the Swedish total population and their use in medical research. *Eur J Epidemiol* 2016;31(2):125-36.

24 von Elm E, Altman DG, Egger M, et al. The Strengthening the Reporting of Observational Studies in Epidemiology (STROBE) Statement: guidelines for reporting observational studies. *Int J Surg* 2014;12(12):1495-9.

25 Ludvigsson JF, Otterblad-Olausson P, Pettersson BU, et al. The Swedish personal identity number: possibilities and pitfalls in healthcare and medical research. *Eur J Epidemiol* 2009;24(11):659-67.

For peer review only - http://bmjopen.bmj.com/site/about/guidelines.xhtml

26 Ludvigsson JF, Andersson E, Ekbom A, et al. External review and validation of the Swedish national inpatient register. *BMC Public Health* 2011;11:450.

27 Wallerstedt SM, Wettermark B, Hoffmann M. The First Decade with the Swedish Prescribed Drug Register - A Systematic Review of the Output in the Scientific Literature. *Basic Clin Pharmacol Toxicol* 2016;119(5):464-69.

28 Statistics Sweden. Longitudinal integration database for health insurance and labour market studies (LISA). 2018 [cited 2019 17 September]. Available from: https://www.scb.se/vara-tjanster/bestalla-mikrodata/vilka-mikrodata-finns/longitudinella-register/longitudinell-integrationsdatabas-for-sjukforsakrings--och-arbetsmarknadsstudier-lisa/ accessed 13 January 2021.

29 Gustafsson K, Kvist J, Eriksson M, et al. Socioeconomic status of patients in a Swedish national self-management program for osteoarthritis compared with the general population-a descriptive observational study. *BMC Musculoskelet Disord* 2020;21(1):10.

30 Valderas JM, Starfield B, Sibbald B, et al. Defining comorbidity: implications for understanding health and health services. *Ann Fam Med* 2009;7(4):357-63.

31 Pratt NL, Kerr M, Barratt JD, et al. The validity of the Rx-Risk Comorbidity Index using medicines mapped to the Anatomical Therapeutic Chemical (ATC) Classification System. *BMJ Open* 2018;8(4):e021122.

32 Charlson ME, Pompei P, Ales KL, et al. A new method of classifying prognostic comorbidity in longitudinal studies: development and validation. *J Chronic Dis* 1987;40(5):373-83.

33 Elixhauser A, Steiner C, Harris DR, et al. Comorbidity measures for use with administrative data. *Med Care* 1998;36(1):8-27.

34 Inacio MC, Pratt NL, Roughead EE, et al. Comparing co-morbidities in total joint arthroplasty patients using the RxRisk-V, Elixhauser, and Charlson Measures: a cross-sectional evaluation. *BMC Musculoskelet Disord* 2015;16:385.

For peer review only - http://bmjopen.bmj.com/site/about/guidelines.xhtml

35 Dominick KL, Dudley TK, Coffman CJ, et al. Comparison of three comorbidity measures for predicting health service use in patients with osteoarthritis. *Arthritis Rheum* 2005;53(5):666-72.

36 Quan H, Sundararajan V, Halfon P, et al. Coding algorithms for defining comorbidities in ICD-9-CM and ICD-10 administrative data. *Med Care* 2005;43(11):1130-9.

37 Molto A, Dougados M. Comorbidity indices. *Clin Exp Rheumatol* 2014;32(5 Suppl 85):S-131-4.

38 Lanza FL, Chan FKL, Quigley EMM, et al. Guidelines for Prevention of NSAID-Related Ulcer Complications. *Official journal of the American College of Gastroenterology | ACG* 2009;104(3):728-38.

39 Ludvigsson JF, Haberg SE, Knudsen GP, et al. Ethical aspects of registry-based research in the Nordic countries. *Clin Epidemiol* 2015;7:491-508.

40 Adler NE, Ostrove JM. Socioeconomic status and health: what we know and what we don't. *Ann N Y Acad Sci* 1999;896:3-15.

41 Braveman P, Gottlieb L. The social determinants of health: it's time to consider the causes of the causes. *Public Health Rep* 2014;129 (Suppl 2):19-31.

42 Reyes C, Garcia-Gil M, Elorza JM, et al. Socio-economic status and the risk of developing hand, hip or knee osteoarthritis: a region-wide ecological study. *Osteoarthritis Cartilage* 2015;23(8):1323-9.

43 Callahan LF, Shreffler J, Siaton BC, et al. Limited educational attainment and radiographic and symptomatic knee osteoarthritis: a cross-sectional analysis using data from the Johnston County (North Carolina) Osteoarthritis Project. *Arthritis Res Ther* 2010;12(2):R46.

44 Agerholm J, Bruce D, Ponce de Leon A, et al. Socioeconomic differences in healthcare utilization, with and without adjustment for need: an example from Stockholm, Sweden. *Scand J Public Health* 2013;41(3):318-25.

For peer review only - http://bmjopen.bmj.com/site/about/guidelines.xhtml

45 Ackerman IN, Busija L. Access to self-management education, conservative treatment and surgery for arthritis according to socioeconomic status. *Best Pract Res Clin Rheumatol* 2012;26(5):561-83.

46 Wetterholm M, Turkiewicz A, Stigmar K, et al. The rate of joint replacement in osteoarthritis depends on the patient's socioeconomic status. *Acta Orthop* 2016;87(3):245-51.

47 Pihl K, Roos EM, Taylor RS, et al. Associations between comorbidities and immediate and one-year outcomes following supervised exercise therapy and patient education - A cohort study of 24,513 individuals with knee or hip osteoarthritis. *Osteoarthritis Cartilage* 2021;29(1):39-49.

48 Hall AJ, Stubbs B, Mamas MA, et al. Association between osteoarthritis and cardiovascular disease: Systematic review and meta-analysis. *Eur J Prev Cardiol* 2016;23(9):938-46.

49 Louati K, Vidal C, Berenbaum F, et al. Association between diabetes mellitus and osteoarthritis: systematic literature review and meta-analysis. *RMD Open* 2015;1(1):e000077.

50 Patterson RE, Frank LL, Kristal AR, et al. A comprehensive examination of health conditions associated with obesity in older adults. *Am J Prev Med* 2004;27(5):385-90.

51 Woolf AD, Pfleger B. Burden of major musculoskeletal conditions. *Bull World Health Organ* 2003;81(9):646-56.

52 Thorstensson CA, Gooberman-Hill R, Adamson J, et al. Help-seeking behaviour among people living with chronic hip or knee pain in the community. *BMC Musculoskelet Disord* 2009;10:153.

53 Thorstensson CA, Dahlberg L, Garellick G. The BOA Register Annual Report 2014. Göteborg; 2015.

54 de Rooij M, van der Leeden M, Cheung J, et al. Efficacy of Tailored Exercise Therapy on Physical Functioning in Patients With Knee Osteoarthritis and Comorbidity: A Randomized Controlled Trial. *Arthritis Care Res (Hoboken)* 2017;69(6):807-16.

For peer review only - http://bmjopen.bmj.com/site/about/guidelines.xhtml

Figure 1 Forest plot with odds ratio (OR) and 95% confidence interval for having comorbidities for the OA population compared with the reference cohort, grouped in disease categories. Reported in crude OR, and in OR adjusted for socioeconomic status (educational level, country of birth and marital status). *The conditions inflammation/pain and pain, identified by the RxRisk Index, were excluded from the analyses.

Figure 2 Forest plot with odds ratio (OR) and 95% confidence interval for having ≥1 comorbidities for the OA population compared with the reference cohort, grouped by age. Reported in crude OR, and in OR adjusted for socioeconomic status (educational level, country of birth and marital status).

For peer review only

For peer review only - http://bmjopen.bmj.com/site/about/guidelines.xhtml

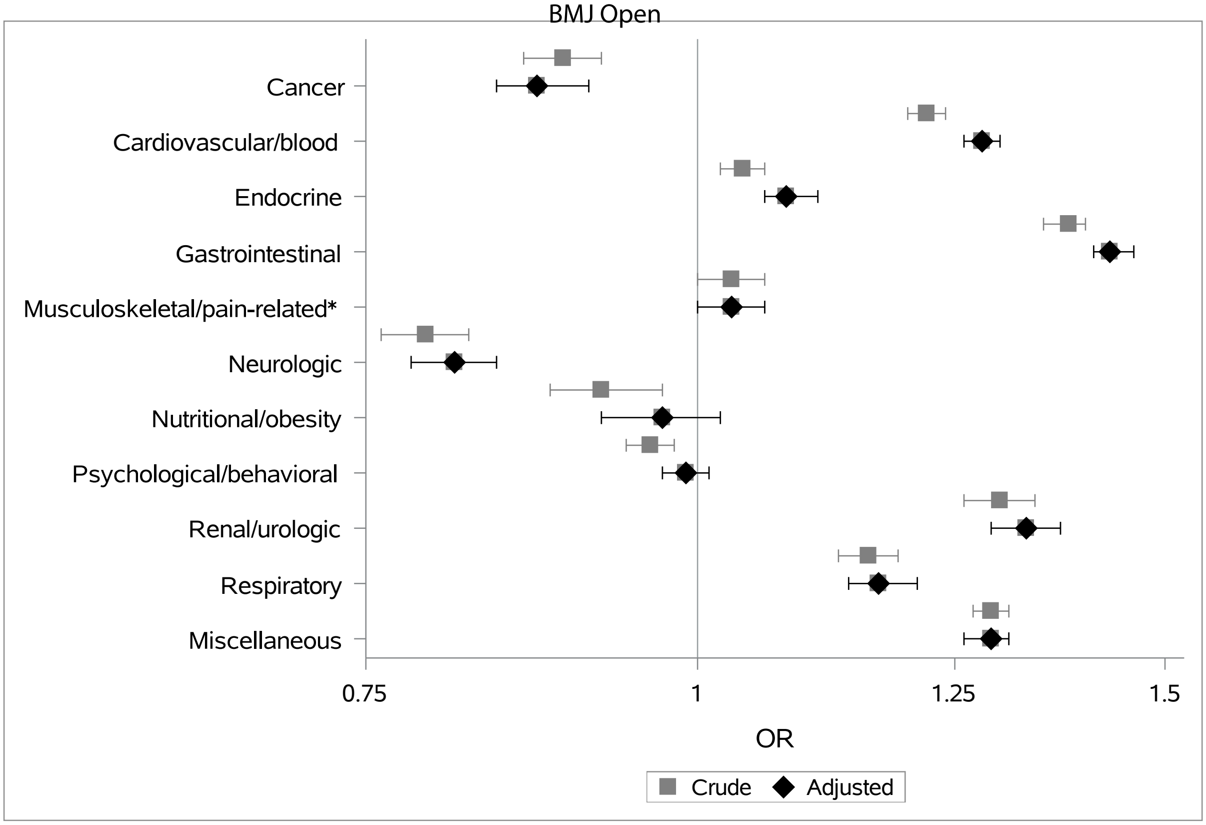
Cancer
Cardiovascular/blood
Endocrine
Gastrointestinal
Musculoskeletal/pain-related*
Neurologic
Nutritional/obesity
Psychological/behavioral
Renal/urologic
Respiratory
Miscellaneous
0.75
1
1.25
1.5
OR
Crude
Adjusted

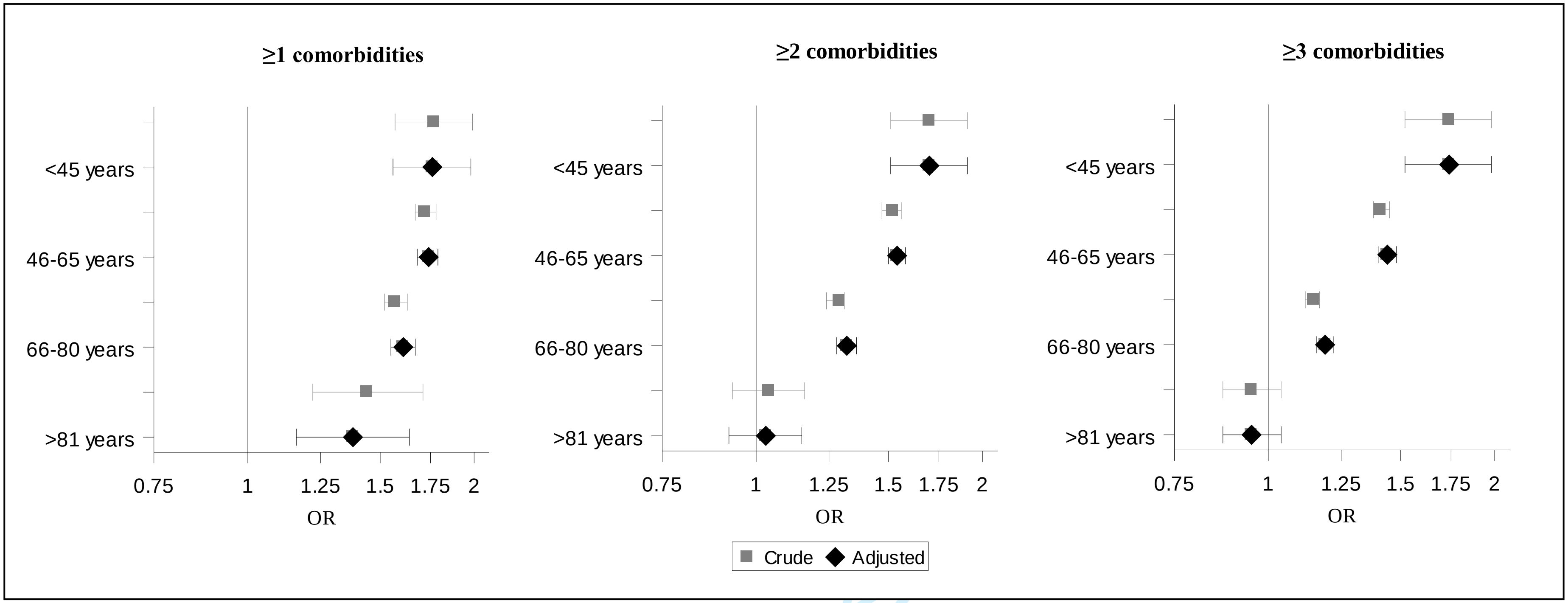

≥1 comorbidities
≥2 comorbidities
≥3 comorbidities
<45 years
46-65 years
66-80 years
>81 years
0.75
1
1.25
1.5
1.75
2
OR
Crude
Adjusted

Supplementary file 1. ATC codes used to identify comorbidities with the RxRisk Index[1]

Comorbidities	ATC codes
Alcohol dependency	N07BB01–N07BB99
Allergies	R01AC01–R01AD60, R06AD02–R06AX27, R06AB04
Anticoagulants	B01AA03–B01AB06, B01AE07, B01AF01, B01AF02, B01AX05
Antiplatelets	B01AC04–B01AC30
Anxiety	N05BA01–N05BA12, N05BE01
Arrhythmia	C01AA05, C01BA01–C01BD01, C07AA07
Benign prostatic hyperplasia	G04CA01–G04CA99, G04CB01, G04CB02*
Bipolar disorder	N05AN01
Chronic airways disease	R03AC02–R03DC03, R03DX05
Congestive heart failure	C03DA02–C03DA99, C07AB02, C07AB07, C07AG02, C07AB12, C03DA04 (C03CA01– C03CC01 and C09AA01–C09AX99, C09CA01– C09CX99)†
Dementia	N06DA02–N06DA04, N06DX01
Depression	N06AA01–N06AG02, N06AX03–N06AX11, N06AX13–N06AX18, N06AX21–N06AX26
Diabetes	A10AA01-A10BX99
Epilepsy	N03AA01–N03AX99
Glaucoma	S01EA01–S01EB03, S01EC03–S01EX99
Gastroesophageal reflux disease	A02BA01–A02BX05
Gout	M04AA01–M04AC01
HIV	J05AE01–J05AE10, J05AF12–J05AG05, J05AR01–J05AR99, J05AX07–J05AX09, J05AX12, J05AF01–J05AF07, J05AF09
Hyperkalaemia	V03AE01
Hyperlipidaemia	C10AA01–C10BX09
Hypertension	C03AA01–C03BA11, C03DB01, C03DB99, C03EA01, C09BA02–C09BA09, C09DA02– C09DA08, C02AB01–C02AC05, C02DB02– C02DB99 (C03CA01–C03CCO1 or C09CA01– C09CX99)§
Hyperthyroidism	H03BA02, H03BB01
Hypothyroidism	H03AA01–H03AA02
Irritable bowel syndrome	A07EC01–A07EC04, A07EA01–A07EA02, A07EA06, L04AA33
Ischaemic heart disease: angina	C01DA02–C01DA14, C01DX16, C08EX02
Ischaemic heart disease: hypertension	C07AA01–C07AA06, C07AA08–C07AB01, C07AB02, C07AG01, C08CA01–C08DB01, C09DB01–C09DB04, C09DX01, C09BB02– C09BB10, C07AB03, C09DX03
Incontinence	G04BD01–G04BD99
Inflammation/pain	M01AB01–M01AH06
Liver failure	A06AD11, A07AA11
Malignancies	L01AA01–L01XX41
Malnutrition	B05BA01–B05BA10
Migraine	N02CA01–N02CX01
Osteoporosis/Paget's	M05BA01–M05BB05, M05BX03, M05BX04, G03XC01, H05AA02
Pain	N02AA01–N02AX02, N02AX06, N02AX52, N02BE51

For peer review only

Pancreatic insufficiency	A09AA02
Parkinson's disease	N04AA01–N04BX02
Psoriasis	D05AA01–D05AA99, D05BB01, D05BB02, D05AX02, D05AC01–D05AC51, D05AX52
Psychotic illness	N05AA01–N05AB02, N05AB06–N05AL07, N05AX07–N05AX13
Pulmonary hypertension	C02KX01–C02KX05
Renal disease	B03XA01–B03XA03, A11CC01–A11CC04, V03AE02, V03AE03, V03AE05
Smoking cessation	N07BA01–N07BA03, N06AX12
Steroid-responsive disease	H02AB01–H02AB10
Transplant	L04AA06, L04AA10, L04AA18, L04AD01, L04AD02

1. Pratt NL, Kerr M, Barratt JD, et al. The validity of the Rx-Risk Comorbidity Index using medicines mapped to the Anatomical Therapeutic Chemical (ATC) Classification System. *BMJ Open* 2018;8(4):e021122.

ATC code: Anatomical Therapeutic Chemical code
*Benign prostatic hyperplasia medicines – must be male.
†Must have at least two medicines prescribed with one of those medicines having an ATC code from C03CA01–C03CC01 and the other having an ATC code from either C09AA01–C09AX99 or C09CA01–C09CX99, at some point within the period of three years prior to baseline
§Can have medicine dispensed with an ATC code C03CA01–C03CC01 or C09AA01–C09AX99, but not both, as this would indicate chronic heart failure.

For peer review only

Supplementary file 2. ICD-10 codes used to identify comorbidities with the Charlson Comorbidity Index and the Elixhauser Comorbidity Index[1]

Comorbidities	ICD-10	
	Charlson Comorbidity Index	Elixhauser Comorbidity Index
AIDS/HIV	B20.x–B22.x, B24.x	B20.x–B22.x, B24.x
Alcohol abuse	-	F10, E52, G62.1, I42.6, K29.2, K70.0, K70.3, K70.9, T51.x, Z50.2, Z71.4, Z72.1
Cardiac arrhythmia	-	I44.1–I44.3, I45.6, I45.9, I47.x–I49.x, R00.0, R00.1, R00.8, T82.1, Z45.0, Z95.0
Blood loss anaemia	-	D50.0
Cerebrovascular disease	G45.x, G46.x, H34.0, I60.x-I69.x	-
Chronic pulmonary disease	I27.8, I27.9, J40.x–J47.x, J60.x–J67.x, J68.4, J70.1, J70.3	I27.8, I27.9, J40.x–J47.x, J60.x–J67.x, J68.4, J70.1, J70.3
Coagulopathy	-	D65–D68.x, D69.1, D69.3–D69.6
Congestive heart failure	I09.9, I11.0, I13.0, I13.2, I25.5, I42.0, I42.5–I42.9, I43.x, I50.x, P29.0	I09.9, I11.0, I13.0, I13.2, I25.5, I42.0, I42.5–I42.9, I43.x, I50.x, P29.0
Deficiency anaemia	-	D50.8, D50.9, D51.x–D53.x
Dementia	F00.x–F03.x, F05.1, G30.x, G31.1	-
Depression	-	F20.4, F31.3–F31.5, F32.x, F33.x, F34.1, F41.2, F43.2
Diabetes (uncomplicated)	E10.0, E10.1, E10.6, E10.8, E10.9, E11.0, E11.1, E11.6, E11.8, E11.9, E12.0, E12.1, E12.6, E12.8, E12.9, E13.0, E13.1, E13.6, E13.8, E13.9, E14.0, E14.1, E14.6, E14.8, E14.9	E10.0, E10.1, E10.9, E11.0, E11.1, E11.9, E12.0, E12.1, E12.9, E13.0, E13.1, E13.9, E14.0, E14.1, E14.9
Diabetes (complicated)	E10.2–E10.5, E10.7, E11.2–E11.5, E11.7, E12.2–E12.5, E12.7, E13.2–E13.5, E13.7, E14.2–E14.5, E14.7	E10.2–E10.8, E11.2–E11.8, E12.2–E12.8, E13.2–E13.8, E14.2–E14.8
Drug abuse	-	F11.x–F16.x, F18.x, F19.x, Z71.5, Z72.2
Fluid and electrolyte disorders	-	E22.2, E86.x, E87.x
Hemiplegia or paraplegia	G04.1, G11.4, G80.1, G80.2, G81.x, G82.x, G83.0–G83.4, G83.9	-
Hypertension[a]	-	I10.x, I11.x–I13.x, I15.x
Hypothyroidism	-	E00.x–E03.x, E89.0
Liver disease	B18.x, K70.0–K70.3, K70.9, K71.3–K71.5, K71.7, K73.x, K74.x, K76.0, K76.2–K76.4, K76.8, K76.9, Z94.4	B18.x, I85.x, I86.4, I98.2, K 70.x, K71.1, K71.3–K71.5, K71.7, K72.x–K74.x, K76.0, K76.2–K76.9, Z94.4
Liver failure	I85.0, I85.9, I86.4, I98.2, K70.4, K71.1, K72.1, K72.9, K76.5, K76.6, K76.7	-
Lymphoma	-	C81.x–C85.x, C88.x, C96.x, C90.0, C90.2
Malignancies	C00.x–C26.x, C30.x–C34.x, C37.x–C41.x, C43.x, C45.x–C58.x, C60.x–C76.x, C81.x–C85.x, C88.x, C90.x–C97.x	-
Metastatic cancer	C77.x–C80.x	C77.x–C80.x
Myocardial infarction	I21.x, I22.x, I25.2	-
Obesity	-	E66.x
Other neurologic disorders	-	G10.x–G13.x, G20.x–G22.x, G25.4, G25.5, G31.2, G31.8, G31.9, G32.x, G35.x–G37.x, G40.x, G41.x, G93.1, G93.4, R47.0, R56.x
Paralysis	-	G04.1, G11.4, G80.1, G80.2, G81.x, G82.x, G83.0–G83.4, G83.9
Peptic ulcer disease	K25.x–K28.x	K25.7, K25.9, K26.7, K26.9, K27.7, K27.9, K28.7, K28.9
Peripheral vascular disease	I70.x, I71.x, I73.1, I73.8, I73.9, I77.1, I79.0, I79.2, K55.1, K55.8, K55.9, Z95.8, Z95.9	I70.x, I71.x, I73.1, I73.8, I73.9, I77.1, I79.0, I79.2, K55.1, K55.8, K55.9, Z95.8, Z95.9
Pulmonary circulation disorders	-	I26.x, I27.x, I28.0, I28.8, I28.9
Psychoses	-	F20.x, F22.x–F25.x, F28.x, F29.x, F30.2, F31.2, F31.5

For peer review only

Renal disease	I12.0, I13.1, N03.2–N03.7, N05.2–N05.7, N18.x, N19.x, N25.0, Z49.0–Z49.2, Z94.0, Z99.2	I12.0, I13.1, N18.x, N19.x, N25.0, Z49.0–Z49.2, Z94.0, Z99.2
Rheumatoid arthritis/collage vascular diseases		L94.0, L94.1, L94.3, M05.x, M06.x, M08.x, M12.0, M12.3, M30.x, M31.0–M31.3, M32.x–M35.x, M45.x, M46.1, M46.8, M46.9
Rheumatic disease	M05.x, M06.x, M31.5, M32.x–M34.x, M35.1, M35.3, M36.0	
Solid tumour without metastasis		C00.x–C26.x, C30.x–C34.x, C37.x–C41.x, C43.x, C45.x–C58.x, C60.x–C76.x, C97.x
Valvular disease		A52.0, I05.x–I08.x, I09.1, I09.8, I34.x–I39.x, Q23.0–Q23.3, Z95.2–Z95.4
Weight loss	-	E40.x–E46.x, R63.4, R64

1. Quan H, Sundararajan V, Halfon P, et al. Coding algorithms for defining comorbidities in ICD-9-CM and ICD-10 administrative data. *Med Care* 2005;43(11):1130-9.
[a]Complicated and uncomplicated combined

For peer review only

Supplementary file 3. Secondary analyses, presenting odds ratio (OR) of having comorbidities for the OA population in the BOA Register with index date between 2008 and 2016, compared with the reference cohort.

	**Total study population**			**Hip OA**			**Knee OA**		
	**Crude OR** (95% CI) **≤2015**	**Adjusted OR** (95% CI)[a] **≤2015**	**Adjusted OR** (95% CI)[b] **≤2015**	**Crude OR** (95% CI) **≤2015**	**Adjusted OR** (95% CI)[a] **≤2015**	**Adjusted OR** (95% CI)[b] **≤2015**	**Crude OR** (95% CI) **≤2015**	**Adjusted OR** (95% CI)[a] **≤2015**	**Adjusted OR** (95% CI)[b] **≤2015**
**Individual comorbidities**[b]									
**≥1**	1.63 (1.58, 1.67)*	1.68 (1.63, 1.72)*	1.70 (1.66, 1.75)*	1.47 (1.40, 1.53)*	1.52 (1.45, 1.59)*	1.54 (1.47, 1.61)*	1.71 (1.65, 1.76)*	1.75 (1.70, 1.81)*	1.78 (1.73, 1.84)*
**≥2**	1.36 (1.33, 1.39)*	1.41 (1.39, 1.45)*	1.44 (1.41, 1.47)*	1.24 (1.19, 1.29)*	1.29 (1.25, 1.34)*	1.32 (1.27, 1.37)*	1.42 (1.39, 1.46)*	1.47 (1.44, 1.51)*	1.50 (1.46, 1.54)*
**≥3**	1.23 (1.21, 1.26)*	1.28 (1.26, 1.31)*	1.31 (1.28, 1.33)*	1.12 (1.08, 1.16)*	1.17 (1.13, 1.21)*	1.19 (1.14, 1.23)*	1.28 (1.26, 1.32)*	1.34 (1.31, 1.37)*	1.36 (1.33, 1.40)*
**Disease categories**									
**Cancer**	0.88 (0.84, 0.92)*	0.87 (0.84, 0.91)*	0.87 (0.84, 0.91)*	0.88 (0.82, 0.95)*	0.87 (0.81, 0.94)*	0.87 (0.81, 0.94)*	0.88 (0.83, 0.92)*	0.88 (0.83, 0.92)*	0.87 (0.83, 0.92)*
**Cardiovascular/ blood**	1.23 (1.20, 1.25)*	1.27 (1.25, 1.30)*	1.29 (1.26, 1.32)*	1.14 (1.10, 1.18)*	1.19 (1.14, 1.23)*	1.20 (1.16, 1.25)*	1.27 (1.24, 1.30)*	1.31 (1.28, 1.34)*	1.33 (1.30, 1.37)*
**Endocrine**	1.04 (1.02, 1.07)*	1.09 (1.06, 1.12)*	1.09 (1.07, 1.12)*	0.96 (0.92, 1.01)	1.02 (0.97, 1.07)	1.02 (0.98, 1.07)	1.08 (1.05, 1.11)*	1.13 (1.10, 1.16)*	1.13 (1.10, 1.16)*
**Gastrointestinal**	1.39 (1.36, 1.42)*	1.45 (1.42, 1.48)*	1.46 (1.43, 1.49)*	1.29 (1.24, 1.34)*	1.35 (1.30, 1.41)*	1.36 (1.31, 1.41)*	1.43 (1.40, 1.47)*	1.49 (1.45, 1.53)*	1.50 (1.46, 1.54)*
**Musculoskeletal/ pain-related**[c]	1.03 (1.00, 1.07)*	1.04 (1.01, 1.08)*	1.04 (1.01, 1.08)*	1.04 (0.98, 1.10)	1.05 (0.99, 1.11)	1.05 (0.99, 1.11)	1.03 (0.99, 1.07)	1.04 (1.00, 1.08)	1.04 (1.00, 1.08)
**Neurologic**	0.78 (0.75, 0.81)*	0.81 (0.77, 0.84)*	0.81 (0.78, 0.84)*	0.71 (0.66, 0.76)*	0.73 (0.68, 0.79)*	0.73 (0.68, 0.79)*	0.81 (0.78, 0.85)*	0.84 (0.80, 0.88)*	0.84 (0.80, 0.88)*

For peer review only

**Nutritional/obesity**	0.94 (0.89, 1.00)*	0.99 (0.94, 1.05)	1.00 (0.94, 1.06)	0.75 (0.67, 0.84)*	0.79 (0.71, 0.88)*	0.80 (0.71, 0.89)*	1.03 (0.97, 1.10)	1.09 (1.02, 1.16)*	1.10 (1.02, 1.17)*
**Psychological/ behavioural**	0.96 (0.94, 0.98)*	0.99 (0.97, 1.02)	0.99 (0.97, 1.02)	0.92 (0.89, 0.96)*	0.95 (0.92, 0.99)*	0.96 (0.92, 1.00)*	0.97 (0.95, 1.00)*	1.01 (0.98, 1.04)	1.01 (0.98, 1.04)
**Renal/urologic**	1.32 (1.28, 1.37)*	1.34 (1.30, 1.39)*	1.35 (1.30, 1.40)*	1.23 (1.16, 1.31)*	1.26 (1.18, 1.34)*	1.27 (1.19, 1.35)*	1.37 (1.31, 1.43)*	1.39 (1.33, 1.44)*	1.39 (1.33, 1.45)*
**Respiratory**	1.16 (1.12, 1.19)*	1.18 (1.14, 1.21)*	1.18 (1.14, 1.21)*	1.09 (1.03, 1.15)*	1.11 (1.05, 1.18)*	1.12 (1.06, 1.18)*	1.19 (1.15, 1.23)*	1.20 (1.16, 1.25)*	1.20 (1.16, 1.25)*
**Miscellaneous**	1.30 (1.28, 1.33)*	1.30 (1.28, 1.33)*	1.30 (1.28, 1.33)*	1.23 (1.19, 1.28)*	1.23 (1.19, 1.28)*	1.23 (1.19, 1.28)*	1.34 (1.31, 1.37)*	1.34 (1.30, 1.37)*	1.34 (1.31, 1.37)*

[a]Adjusted for socioeconomic status (educational level, country of birth and marital status).

[b]Adjusted for socioeconomic status (educational level, country of birth, marital status, income and employment, and family type).

*Statistically significant results.

For peer review only

Reporting checklist for cross sectional study.

Based on the STROBE cross sectional guidelines.

Instructions to authors

Complete this checklist by entering the page numbers from your manuscript where readers will find each of the items listed below.

Your article may not currently address all the items on the checklist. Please modify your text to include the missing information. If you are certain that an item does not apply, please write "n/a" and provide a short explanation.

Upload your completed checklist as an extra file when you submit to a journal.

In your methods section, say that you used the STROBE cross sectionalreporting guidelines, and cite them as:

von Elm E, Altman DG, Egger M, Pocock SJ, Gotzsche PC, Vandenbroucke JP. The Strengthening the Reporting of Observational Studies in Epidemiology (STROBE) Statement: guidelines for reporting observational studies.

		Reporting Item	Page Number
**Title and abstract**			
Title	#1a	Indicate the study's design with a commonly used term in the title or the abstract	page 1

For peer review only

Abstract	#1b	Provide in the abstract an informative and balanced summary of what was done and what was found	page 3
**Introduction**			
Background / rationale	#2	Explain the scientific background and rationale for the investigation being reported	page 5
Objectives	#3	State specific objectives, including any prespecified hypotheses	page 6
**Methods**			
Study design	#4	Present key elements of study design early in the paper	page 6
Setting	#5	Describe the setting, locations, and relevant dates, including periods of recruitment, exposure, follow-up, and data collection	page 6-7
Eligibility criteria	#6a	Give the eligibility criteria, and the sources and methods of selection of participants.	page 6-7
	#7	Clearly define all outcomes, exposures, predictors, potential confounders, and effect modifiers. Give diagnostic criteria, if applicable	page 9-10, and 14
Data sources / measurement	#8	For each variable of interest give sources of data and details of methods of assessment	page 9-10, and 14, and Supplemenary file 1-2

For peer review only

		(measurement). Describe comparability of assessment methods if there is more than one group. Give information separately for for exposed and unexposed groups if applicable.	
Bias	#9	Describe any efforts to address potential sources of bias	page 7
Study size	#10	Explain how the study size was arrived at	page 6-7
Quantitative variables	#11	Explain how quantitative variables were handled in the analyses. If applicable, describe which groupings were chosen, and why	page 14
Statistical methods	#12a	Describe all statistical methods, including those used to control for confounding	page 14
Statistical methods	#12b	Describe any methods used to examine subgroups and interactions	page 14
Statistical methods	#12c	Explain how missing data were addressed	n/a, there was only missing data for the variables that were adjusted for (to a small extend), as described in table 1 page 7

For peer review only

Statistical methods	#12d	If applicable, describe analytical methods taking account of sampling strategy	page 14
Statistical methods	#12e	Describe any sensitivity analyses	page 14
**Results**			
Participants	#13a	Report numbers of individuals at each stage of study—eg numbers potentially eligible, examined for eligibility, confirmed eligible, included in the study, completing follow-up, and analysed. Give information separately for for exposed and unexposed groups if applicable.	page 9 and 14
Participants	#13b	Give reasons for non-participation at each stage	page 9
Participants	#13c	Consider use of a flow diagram	page 9
Descriptive data	#14a	Give characteristics of study participants (eg demographic, clinical, social) and information on exposures and potential confounders. Give information separately for exposed and unexposed groups if applicable.	page 8 and 16
Descriptive data	#14b	Indicate number of participants with missing data for each variable of interest	page 8

For peer review only

Outcome data	#15	Report numbers of outcome events or summary measures. Give information separately for exposed and unexposed groups if applicable.	page 8, 11-12 and 16-19
Main results	#16a	Give unadjusted estimates and, if applicable, confounder-adjusted estimates and their precision (eg, 95% confidence interval). Make clear which confounders were adjusted for and why they were included	page 17-19
Main results	#16b	Report category boundaries when continuous variables were categorized	page 7, table 1 - age
Main results	#16c	If relevant, consider translating estimates of relative risk into absolute risk for a meaningful time period	n/a, not relevant
Other analyses	#17	Report other analyses done—e.g., analyses of subgroups and interactions, and sensitivity analyses	page 19

Discussion

Key results	#18	Summarise key results with reference to study objectives	page 19-20
Limitations	#19	Discuss limitations of the study, taking into account sources of potential bias or	page 22-23

For peer review only

		imprecision. Discuss both direction and magnitude of any potential bias.	
Interpretation	#20	Give a cautious overall interpretation considering objectives, limitations, multiplicity of analyses, results from similar studies, and other relevant evidence.	page 21-22
Generalisability	#21	Discuss the generalisability (external validity) of the study results	page 23
**Other Information**			
Funding	#22	Give the source of funding and the role of the funders for the present study and, if applicable, for the original study on which the present article is based	page 25

Notes:

- 7: page 9-10, and 14
- 8: page 9-10, and 14, and Supplemenary file 1-2
- 12c: n/a, there was only missing data for the variables that were adjusted for (to a small extend), as described in table 1 page 7
- 13a: page 9 and 14
- 14a: page 8 and 16
- 15: page 8, 11-12 and 16-19

For peer review only

- 16b: page 7, table 1 - age
- 16c: n/a, not relevant The STROBE checklist is distributed under the terms of the Creative Commons Attribution License CC-BY. This checklist was completed on 25. January 2021 using https://www.goodreports.org/, a tool made by the EQUATOR Network in collaboration with Penelope.ai

For peer review only
